# Supplementary material for: Partitioning of fatty acids between membrane and storage lipids controls ER membrane expansion
Source: EMBO J. 2025 Jan 3;44(3):781–800. doi: 10.1038/s44318-024-00355-3 (PMC11790888; doi:10.1038/s44318-024-00355-3)
Supplement: Supplementary file 1 — Appendix [file 44318_2024_355_MOESM1_ESM.pdf]

Appendix for

Partitioning of fatty acids between membrane and storage lipids controls ER membrane expansion

Pawel K. Lysyganicz, Antonio D. Barbosa, Shoily Khondker, Nicolas A. Stewart, George M. Carman, Phillip J. Stansfeld, Marcus K. Dymond, Symeon Siniossoglou

Table of Contents:

Appendix Figure S1 page 2
Appendix Figure S2 page 3
Appendix Figure S3 page 4
Appendix Figure S4 page 5
Appendix Figure S5 page 6
Appendix Figure S6 page 7
Appendix Figure S7 page 8
Appendix Figure S8 page 9
Appendix Figure S9 page 10
Appendix Figure S10 page 11
Appendix Figure S11 page 12
Appendix Table S1 page 13
Appendix Table S2 page 15
Appendix Table S3 page 16

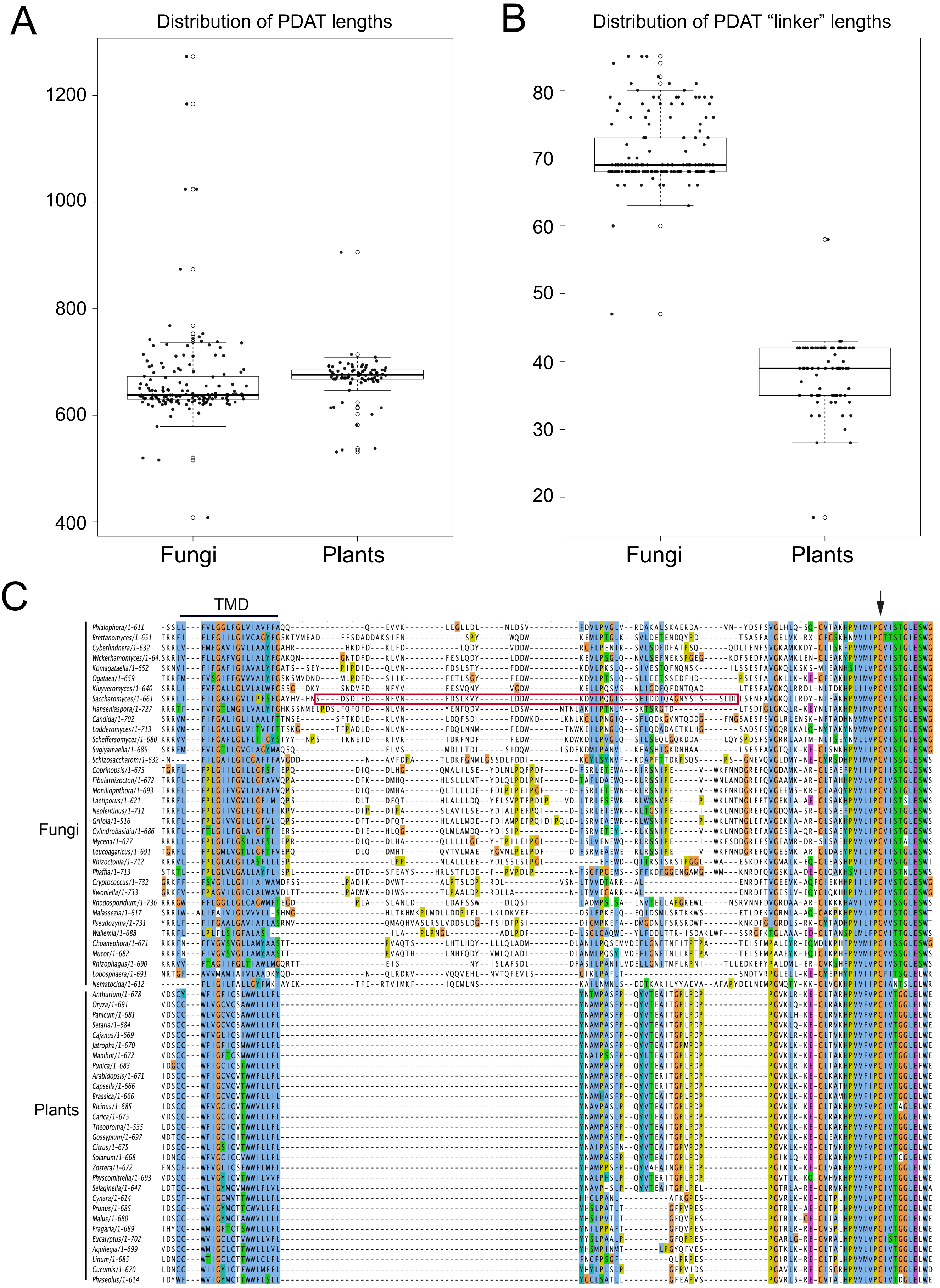

Appendix Figure S1: Comparison of “linker” segments between fungal and plant PDATs. Distribution of (A) full-length protein sequences and (B) “linker” sequence lengths between fungal and plant PDATs. Linker sequence lengths are calculated from the end of the respective transmembrane domains to a conserved glycine residue found in all PDATs and, close to start of the PDAT domain. (C) Multiple sequence alignment (Clustal Omega) of the linker sequence from fungal or plant PDATs; the transmembrane domain (TMD) and the conserved glycine residue are indicated; the red box highlights residues Ser104 - Asp152, which are removed in Lro1*. Sequences are from the fungal and plant PDAT lists published in Barbosa *et al*, (2019).

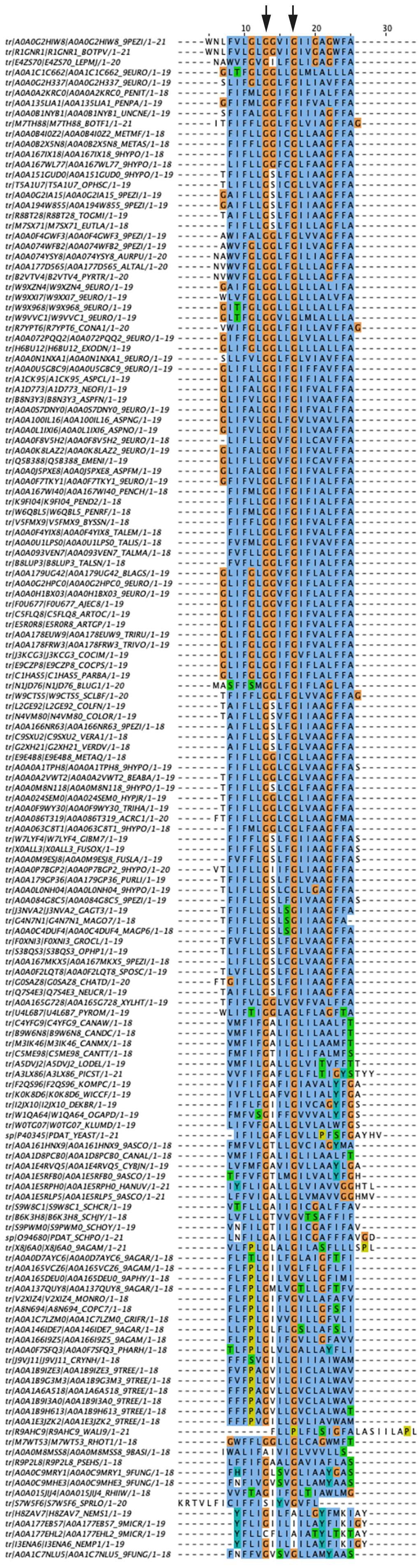

Appendix Figure S2: Multiple sequence alignment (Clustal Omega) of the predicted transmembrane domains of the fungal members of the PDAT family (Barbosa *et al*., 2019). Arrows indicate the position of the conserved GxxxG motif.

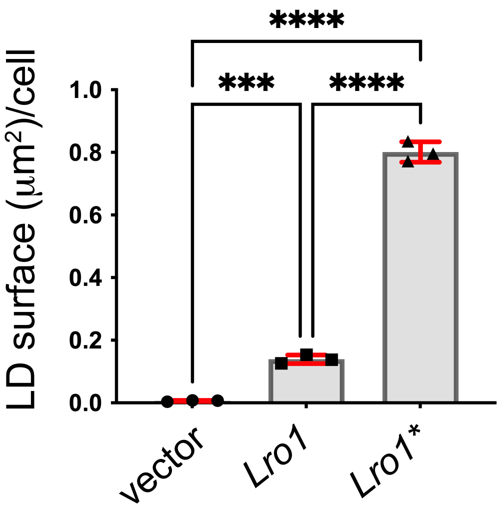

Appendix Figure S3: Quantification of the BODIPY 493/503 labelling of 4Δ cells carrying the denoted plasmids; data are means ± SD from three experiments with at least 75 cells measured per strain per experiment. Statistical analysis was performed by one way ANOVA with Šidák correction; *** $p<0.001$, ****$p<0.0001$ (vector vs Lro1 $p=0.0006$; vector vs Lro1* $p<0.0001$; Lro1 vs Lro1* $p<0.0001$).

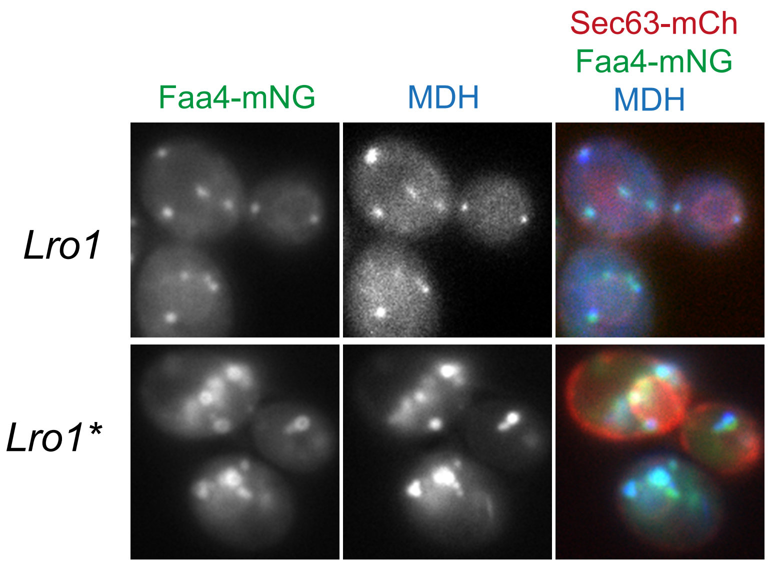

Appendix Figure S4: Cells co-expressing a chromosomally integrated Faa4-mNG, Sec63-mCh and Lro1 or Lro1* from a centromeric vector, were stained with monodansylpentane (MDH) to visualize LDs.

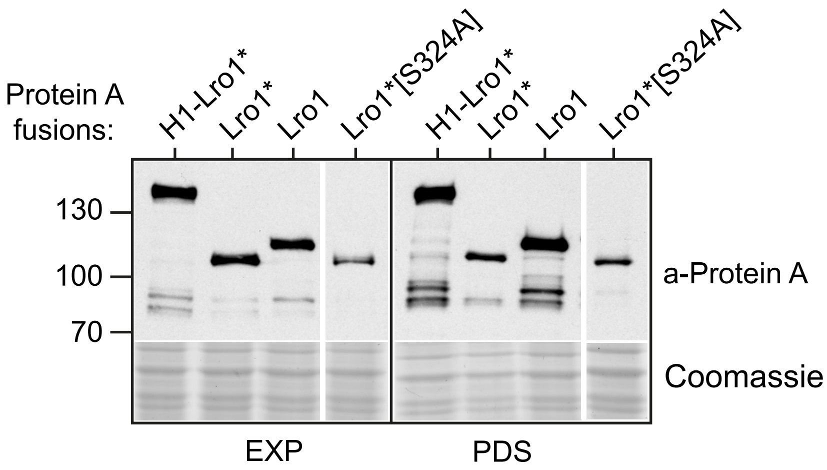

Appendix Figure S5: Western blots of protein extracts from 4Δ cells expressing the indicated Lro1-Protein A fusions from centromeric vectors cultured in the exponential (EXP) or post-diauxic shift (PDS) phases. Lower panels show the corresponding commassie stained gels.

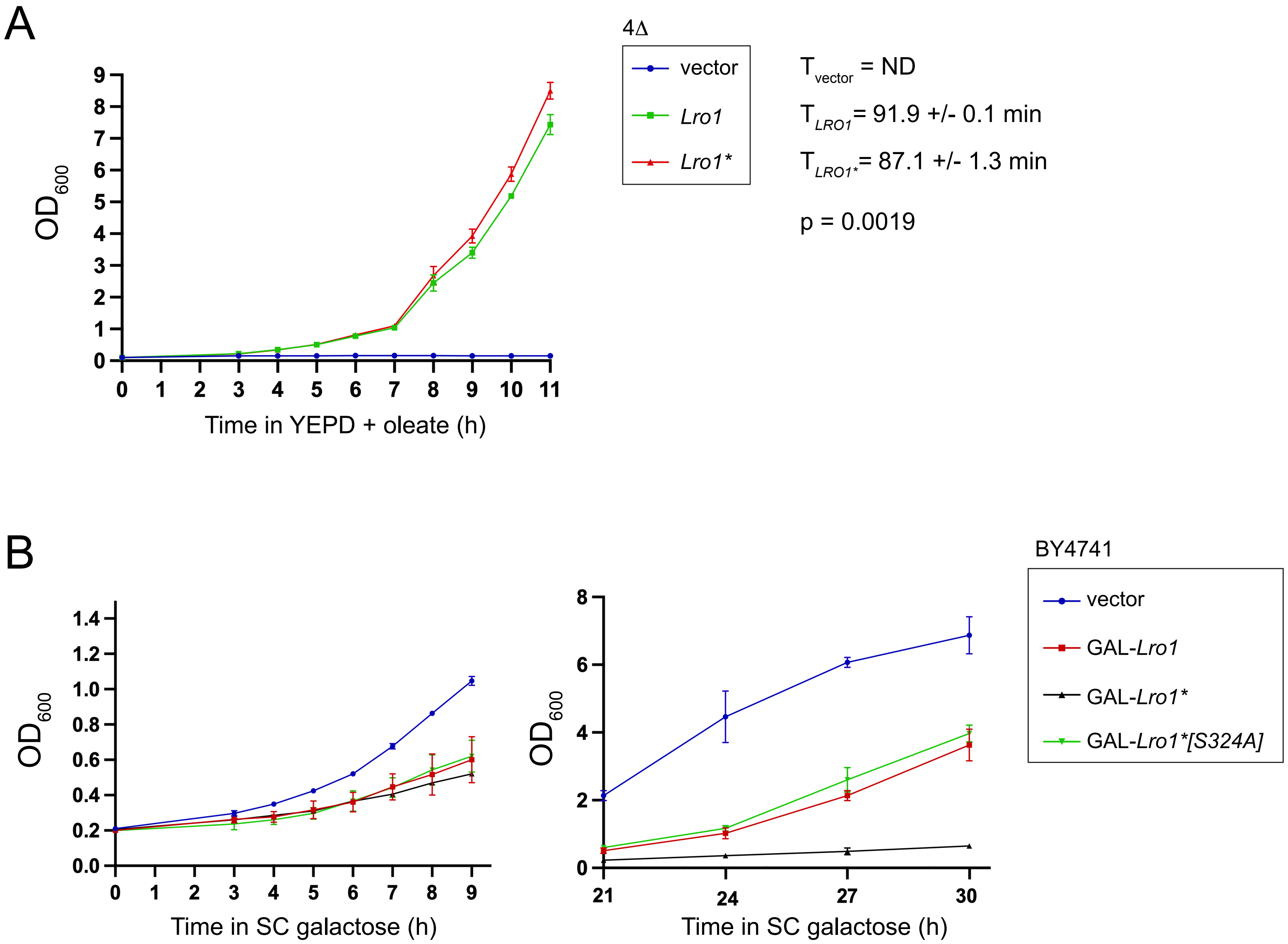

Appendix Figure S6: (A) Exponentially growing 4Δ cells carrying an empty vector or the indicated *LRO1* constructs were inoculated into YEPD medium containing 1mM oleate and the optical densities were measured every hour. Values are means from three different transformants per strain; the generation time (T) for each strain is also shown. (B) Exponentially growing BY4741 cells in raffinose-containing media, carrying the indicated constructs, were transferred into galactose-containing media and their growth was monitored for 9 hours (left panel). Cells were then diluted to an optical density of 0.05 and their growth was monitored for a further 21 hours (right panel). Values are means from three different transformants per strain, except for GAL-*Lro1** which are means of two.

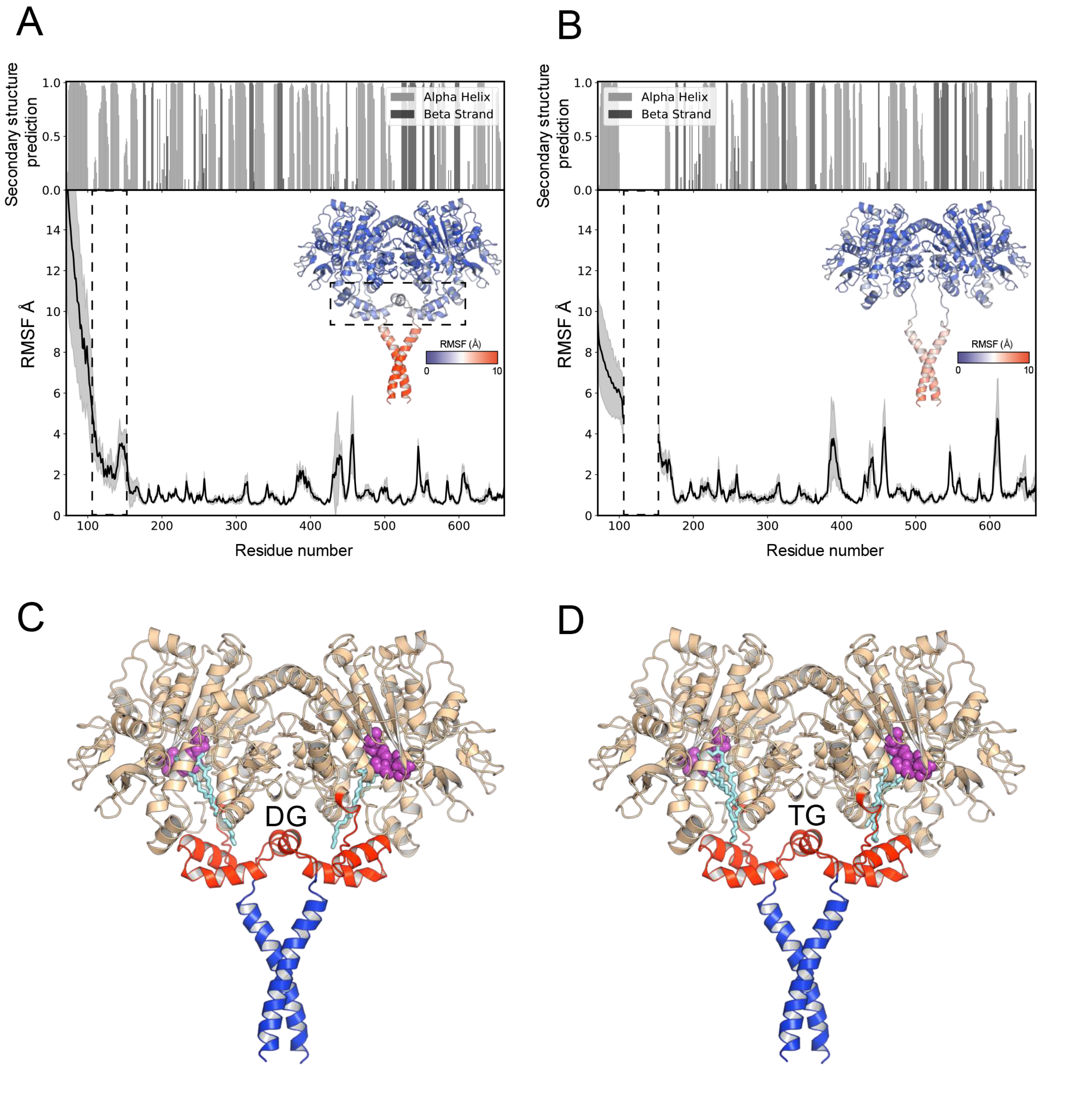

Appendix Figure S7: Ca Root Mean Square Fluctuations and secondary structure retention for the residues in the atomistic MD simulations of (A) wild-type Lro1 and (B) Lro1*. Here the simulations are first aligned based on the soluble domain from residues 153 to 661 before fluctuation analysis. The data shows that this core soluble domain of Lro1 and Lro1* is similarly dynamic in both structures. Despite the low confidence from AlphaFold, the segment Ser104 to Asp152 retains its secondary structure in the simulations and is also relatively stable. For both systems, the transmembrane (TM) helices show the greatest fluctuations, whilst retaining their secondary structure. This is not unexpected as the TM domain is largely uncoupled from the soluble domain in the Lro1 dimer. Chai-1 modelling of (C) diacylglycerol (DG) and (D) triacylglycerol (TG) binding predicts both substrate and product to the active site residues shown in purple van der Waal spheres.

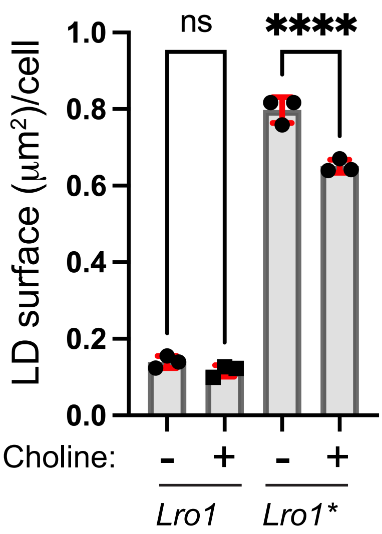

Appendix Figure S8: Quantification of the BODIPY 493/503 labelling of BY4741 expressing Lro1 or Lro1*, in the presence or absence of 1mM choline for 1 hour. Data are means ± SD from three experiments with at least 150 cells measured per strain per experiment. Statistical analysis was performed by one way ANOVA with Šidák correction; ****p<0.0001 (- choline vs + choline for Lro1* p<0.0001; ns, not significant).

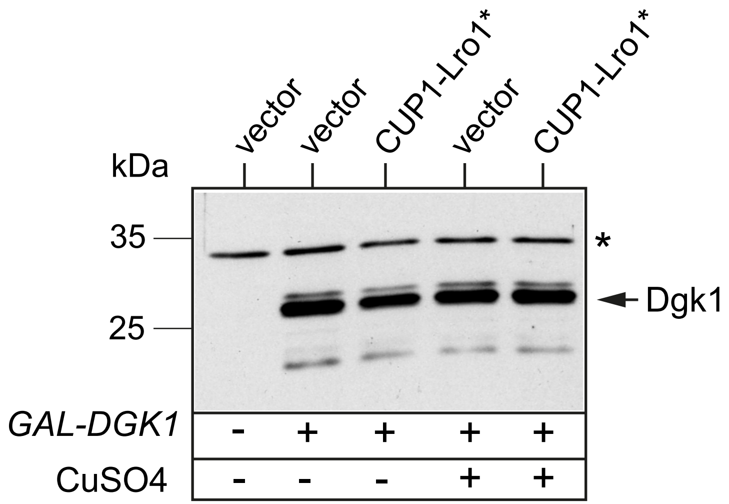

Appendix Figure S9: Wild-type cells carrying the denoted plasmids, were grown and imaged as described in Fig. 6B; at the end-point of the assay (2 hours copper), cells were lysed and the extracts were analyzed by western blot using anti-Dgk1 antibodies. The star indicates a non-specific band. Note that the antibody does not detect the endogenous Dgk1 protein.

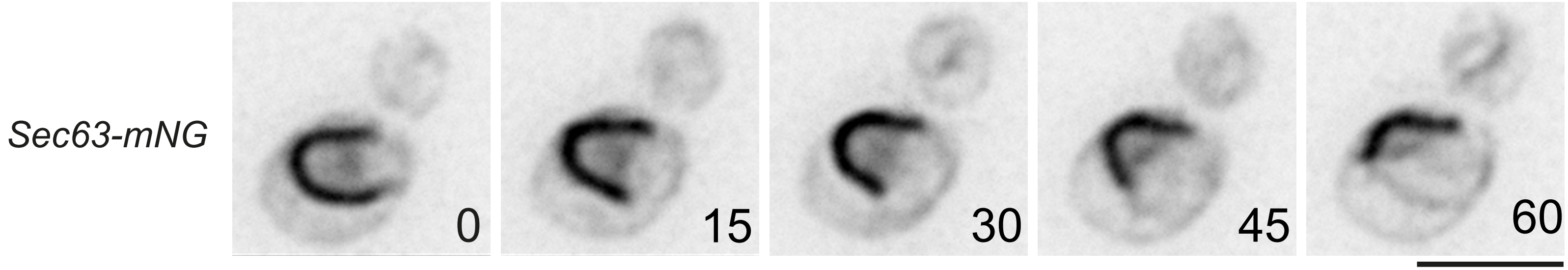

Appendix Figure S10: Time lapse imaging of cells grown as detailed in Fig. 6B, to express sequentially Gal-Dgk1 and then Cup1-Lro1*. The sequence starts after cells were growing for 50 min in glucose/copper sulphate-containing media, corresponding to time point 0 of the time-lapse. Individual frames and the time points (in min) when they were captured are shown. Scale bar, 5μm.

LRO1/vector

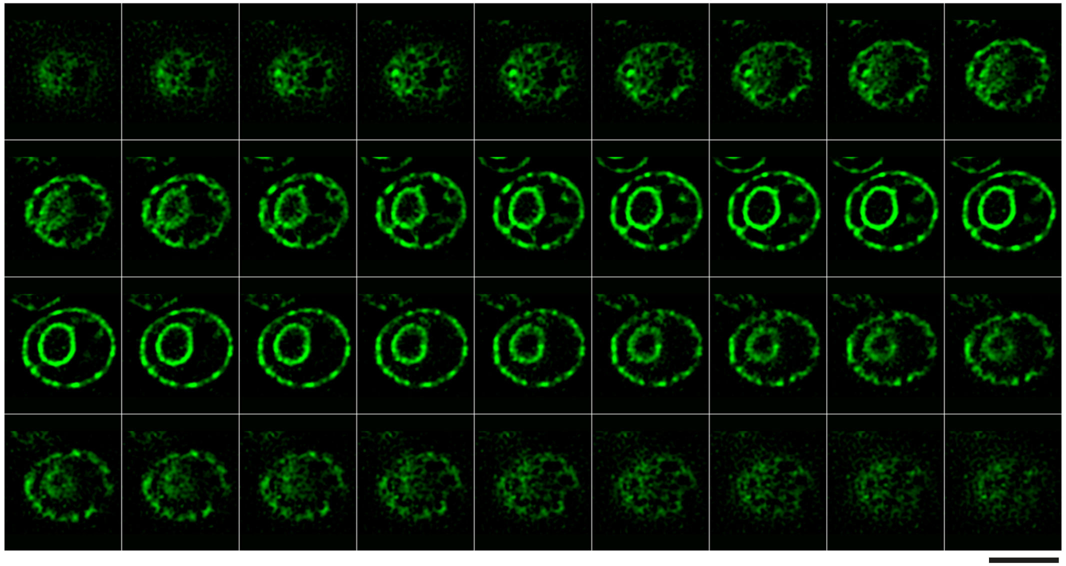

LRO1/PAH1-7A*

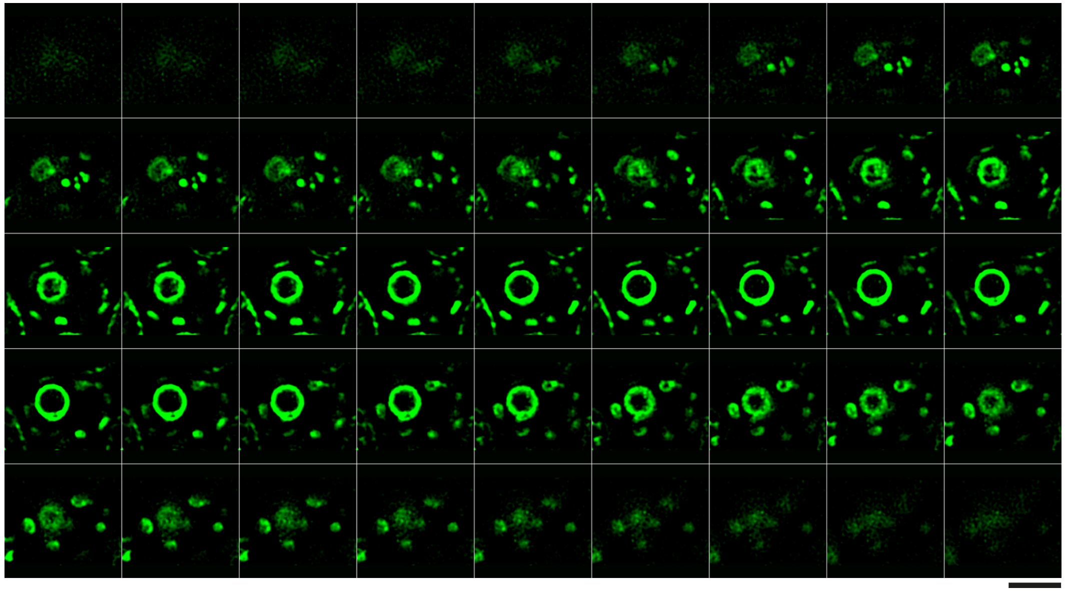

Appendix Figure S11: Sequential optical sections obtained by super resolution microscopy as described in Methods; cells shown are the same as the those in Fig. 7F. Scale bar, 3μm.

**Plasmid description**	**Source/reference**	**Name**
LRO1 under control of *LRO1* promoter in *CEN/URA3* vector	Barbosa et al, 2019	YCplac33-*LRO1*
*LRO1** under control of *LRO1* promoter in *CEN/URA3* vector	This paper	YCplac33-*LRO1**
*HEH1[163-454]-LRO1** under control of *NOP1* promoter in *CEN/URA3* vector	This paper	YCplac33-*H1-LRO1**
LRO1[S324A]* under control of *LRO1* promoter in *CEN/URA3* vector	This paper	YCplac33-*LRO1*[S324A]*
LRO1-mCherry under control of *LRO1* promoter in *CEN/URA3* vector	Barbosa et al, 2019	YCplac33-*LRO1-mCh*
LRO1-mCherry* under control of *LRO1* promoter in *CEN/URA3* vector	This paper	YCplac33-*LRO1*-mCh*
HEH1[163-454]-LRO1-mCherry* under control of *NOP1* promoter in *CEN/URA3* vector	This paper	YCplac33-*H1-LRO1*-mCh*
LRO1[S324A]-mCherry* under control of *LRO1* promoter in *CEN/URA3* vector	This paper	YCplac33-*LRO1*[S324A]-mCh*
LRO1[GS string]-mCherry under control of *LRO1* promoter in *CEN/URA3* vector	This paper	YCplac33-*LRO1*[GS string]-mCh*
LRO1-Protein A under control of *LRO1* promoter in *CEN/URA3* vector	This paper	YCplac33-*LRO1-PtA*
LRO1-Protein A* under control of *LRO1* promoter in *CEN/URA3* vector	This paper	YCplac33-*LRO1*-PtA*
HEH1[163-454]-LRO1-Protein A* under control of *NOP1* promoter in *CEN/URA3* vector	This paper	YCplac33-*H1-LRO1*-PtA*
LRO1[S324A]-Protein A* under control of *LRO1* promoter in *CEN/URA3* vector	This paper	YCplac33-*LRO1*[S324A]-PtA*
LRO1 under control of *GAL1/10* promoter in *2μ/LEU2* vector	Barbosa et al, 2019	YEplac181-*GAL1/10-LRO1*
LRO1[S324A] under control of *GAL1/10* promoter in *2μ/LEU2* vector	Barbosa et al, 2019	YEplac181-*GAL1/10-LRO1[S324A]*
*LRO1** under control of *GAL1/10* promoter in *2μ/LEU2* vector	This paper	YEplac181-*GAL1/10-LRO1**
LRO1[S324A]* under control of *GAL1/10* promoter in *2μ/LEU2* vector	This paper	YEplac181-*GAL1/10-LRO1*[S324A]*
*LRO1** under control of *CUP1* promoter in *2μ/URA3* vector	This paper	YEplac195-*CUP1-LRO1**
*LRO1** under control of *CUP1* promoter in *CEN/URA3 vector*	This paper	YCplac33-*CUP1-LRO1**
LRO1[S324A]* under control of *CUP1* promoter in *CEN/URA3 vector*	This paper	YCplac33-*CUP1-LRO1*[S324A]*
*LRO1** under control of *CUP1* promoter in *CEN/LEU2 vector*	This paper	YCplac111-*CUP1-LRO1**
PAH1-7A under control of *GAL1/10* promoter in *2μ/LEU2* vector	O'Hara et al, 2006	YEplac181-*GAL1/10-PAH1-7A*
DGK1 under control of *GAL1/10* promoter in *2μ/LEU2* vector	Han et al, 2008	YEplac181-*GAL1/10-DGK1*
DGK1 under control of *GAL1/10* promoter in *2μ/URA3* vector	Karanasios et al, 2010	YEplac195-*GAL1/10-DGK1*
PAH1-GFP under control of *PAH1* promoter in *CEN/LEU2 vector*	Karanasios et al, 2010	YCplac111-*PAH1-GFP*

PSR1[1-28]-mCherry under control of *PSR1* promoter in integrative/*HIS3* vector	This study	pRS403-*PSR1[1-28]-mCh*
LRO1-mNeonGreen* under control of *LRO1* promoter in *CEN/LEU2 vector*	This study	YCplac111-*LRO1*-mNG*
dgk1::HphNT1 cassette cloned in CEN/URA3 vector	This study	YCplac33-*dgk1::HphNT1*

Appendix Table S1
Plasmids used in this study.

Oligonucleotide name	Olinonucleotide sequence
LROL153FOR	TTTGGATCCCTCAGTGAAAATTTTGCCGT
STRINGFOR	TTTGGATCCGTCAATGCTGGCGGC
STRINGREV	TTTGGATCCATCAAAATCACCGGA
CUP1LROFOR	TTTTGTCGACATGGGCACACTGTTTCGAAGA
SEC63NGFOR	CCATGAATATGAAAGTTCGTGATTCTC
SEC63NGREV	TAAATGGCGACGAACAAGATGCTAT
RTN1MCHFOR	AGAGTCTAAGGCTTACACATCTTC
RTN1MCHREV	TGAGCACTTTTAACTGCAATGGTT
FAA4FW	AGAAGAGATATTCTAGCGGCTGTCAAGCCAGATGTGGAAAGAGTTTATAAAGAAAACACTGGTGACGGTGCTGGTTTA
FAA4S2	AAACGTAGTGTTTATGAAGGGCAGGGGGGAAAGTAAAAAACTATGTCTTCCTTTAATCGATGAATTCGAGCTCG
NSR1F2	ATTGGGCAGATCAAGAAATACCGCTTCTTTCGCTGGTTCAAAGAAAACATTTGAT CGG ATC CCC GGG TTA ATT AA
NSR1R1	AGTAATTAACGTAAAAAGAGAAAAAATTGAAATTGAAATTCATTTCATTTTCTCA GAATTCGAGCTCGTTTAAAC
VPH1R1	TTTATTATTTAATGAAGTACTTAAATGTTTCGCTTTTTTTAAAAGTCCTCAAAAT GAATTCGAGCTCGTTTAAAC
VPH1F2	TGAGTATAAAGACATGGAAGTCGCTGTTGCTAGTGCAAGCTCTTCCGCTTCAAGC CGGATCCCCGGGTTAATTAA
ATG1S1	CTA CCC CAT ATT TTC AAA TCT CTT TTA CAA CAC CAG ACG AGA AAT TAA GAA AAT GCG TAC GCT GCA GGT CGA C
ATG1S2	GAA AAT ATA GCA GGT CAT TTG TAC TTA ATA AGA AAA CCA TAT TAT GCA TCA CTT AAT CGA TGA ATT CGA GCT CG
SUR4S2	GTTAACATTTAATTTTTTTCTTTTTCATTCGCTGTCAAAAATTCTCGCTTCCTATTTAATCGATGAATTCGAGCTCG
SUR4S3	TCCGGTTCTTCTACTGGTGTCAAGACCTCTAACACCAAGGTCTCTTCCAGGAAAGCTCGTACGCTGCAGGTCGAC
ATG15S1	AGAACTGATCTAGGCATTACAATTAAAGGAAACAAGGGAAATATTCTATTGAATGCGTACGCTGCAGGTCGAC
ATG15S2	AAGGGCGCATAGGCCCTAAAACAACACTAGGGTCATAATAGATGTATGGGTCTTAATCGATGAATTCGAGCTCG
ALE1S1	CGCATACGCCAAGACAAACCGTGGTGATTTAATTCTGCTGCTGATCGCTTCCAACATGCGTACGCTGCAGGTCGAC
ALE1S2	GGAAAATAAGACAACAAGACTGTGACTTCCACACGCATCTGTCGTTTTTGGCCAT ATCGATGAATTCGAGCTCG
FORNGREEN	TTTGGATCCGTCTCTAAGGGTGAAGAAGA
REVNGREEN	TTTGGATCCCTTGTACAATTCGTCCATAC

Appendix Table S2
Oligonucleotide sequences used in this study.

Genotype	Source/reference	Strain
MATa his3Δ1 leu2Δ0 met15Δ0 ura3Δ0	Open Biosystems	BY4741
MATα his3Δ1 leu2Δ0 lys2Δ0 ura3Δ0	Open Biosystems	BY4742
MATα his3Δ1 leu2Δ0 lys2Δ0 ura3Δ0 met15Δ0 are1::KanMX are2::KanMX trp1::URA lro1::TRP dga1::Lox-HIS-Lox	Jacquier et al., 2011	RSY3077 (a.k.a. 4Δ)
BY4741 *FAA4-mNG::spHIS5*	This paper	SS3361
BY4741 *lro1Δ::KanMX*	Barbosa et al., 2019	SS3006
BY4741 *lro1Δ::KanMX HIS3::pRS403-NOP1-RFP*	This paper	SS3007
BY4741 *lro1Δ::KanMX ale1Δ::HphNT1*	This paper	SS3387
BY4741 *lro1Δ::HIS3 SEC63-mNG::KanMX*	This paper	SS3430
BY4741 *SEC63-mNG::KanMX*	This paper	SS3357
BY4741 *SEC63-mNG::KanMX RTN1-mCh::HIS3*	This paper	SS3359
BY4741 *SEC63-mNG::KanMX VPH1-mCh:: HIS3MX6*	This paper	SS3448
BY4741 *atg1Δ::HphNT1*	This paper	SS2708
BY4741 *atg1Δ::HphNT1 HIS3::pRS403-SEC63-mCh*	This paper	SS3251
BY4741 *HIS3::pRS403-SEC63-mCh*	This paper	SS3230
BY4741 *SEC63-mNG::KanMX HIS3::pRS403-PSR1[1-28]-mCh*	This paper	SS3434
BY4742 *pah1Δ::HIS3 pURA-PAH1*	This paper	SS2039
BY4742 *pah1Δ::HIS3 pURA-PAH1 SUR4-GFP::HphNT1*	This paper	SS3261
BY4742 *pah1Δ::HIS3 pURA-PAH1 NSR1-GFP::HphNT1*	This paper	SS3446
BY4741 *lro1Δ::KanMX NSR1-GFP:: HIS3MX6*	This paper	SS3101
BY4742 *gpt2Δ::KanMX*	Open Biosystems	SS1236
BY4742 *sct1Δ::KanMX*	Open Biosystems	SS1237
BY4742 *slc1Δ::KanMX*	Open Biosystems	SS1239
BY4742 *tgl3Δ::KanMX tgl4Δ::KanMX tgl5Δ::KanMX*	Sepp Kohlwein	SS2866
BY4741 *atg15Δ::HphNT1*	This paper	SS3367
BY4741 *ale1Δ::HphNT1*	This paper	SS3376
BY4741 *dgk1Δ::HphNT1*	This paper	SS3044

Appendix Table S3
Yeast strains used in this study.
