# Supplementary figures and images for: Partitioning of fatty acids between membrane and storage lipids controls ER membrane expansion
Source: EMBO J. 2025 Jan 3;44(3):781–800. doi: 10.1038/s44318-024-00355-3 (PMC11790888; doi:10.1038/s44318-024-00355-3)

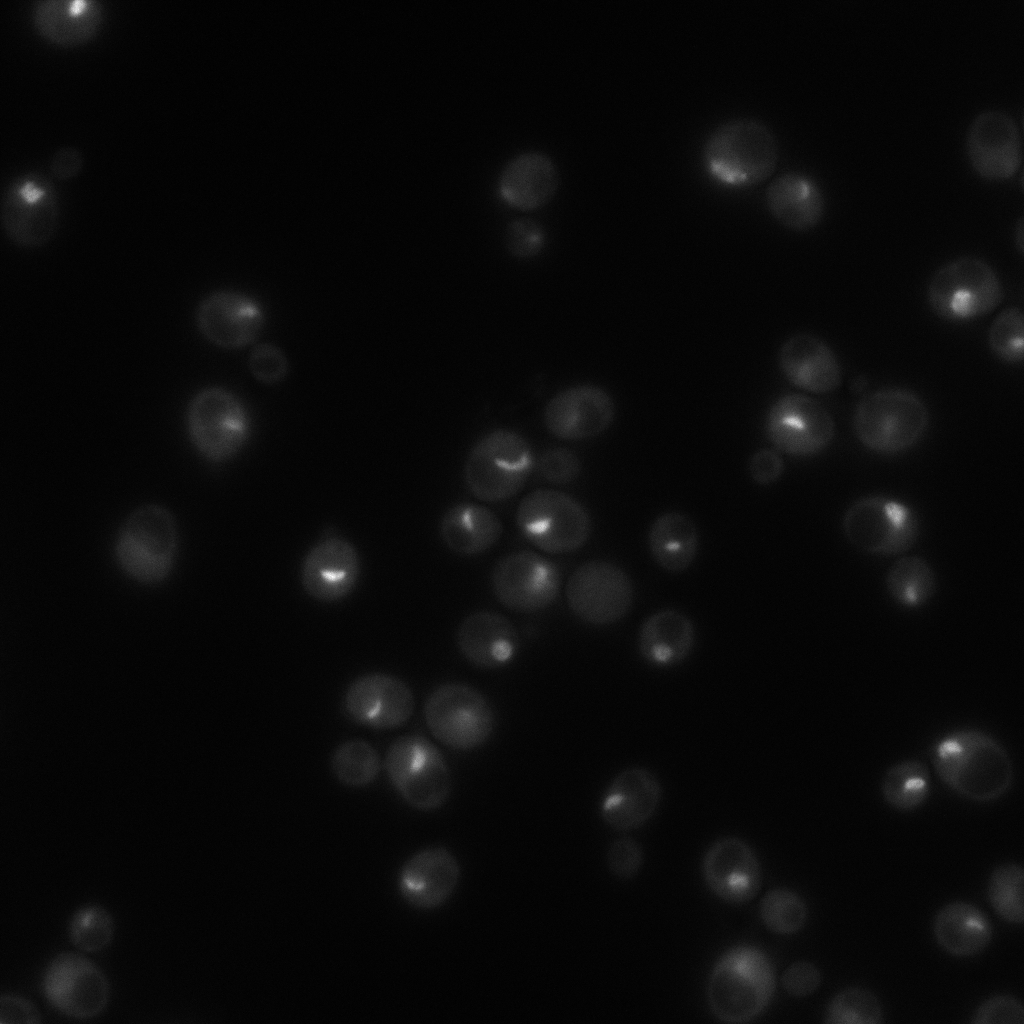

Supplement: Supplementary file 3 — Source data Fig. 2 [file 44318_2024_355_MOESM3_ESM.zip › Figure 2/2F/C1-Snap-1739 (Nsr1-GFP EXP).tif]

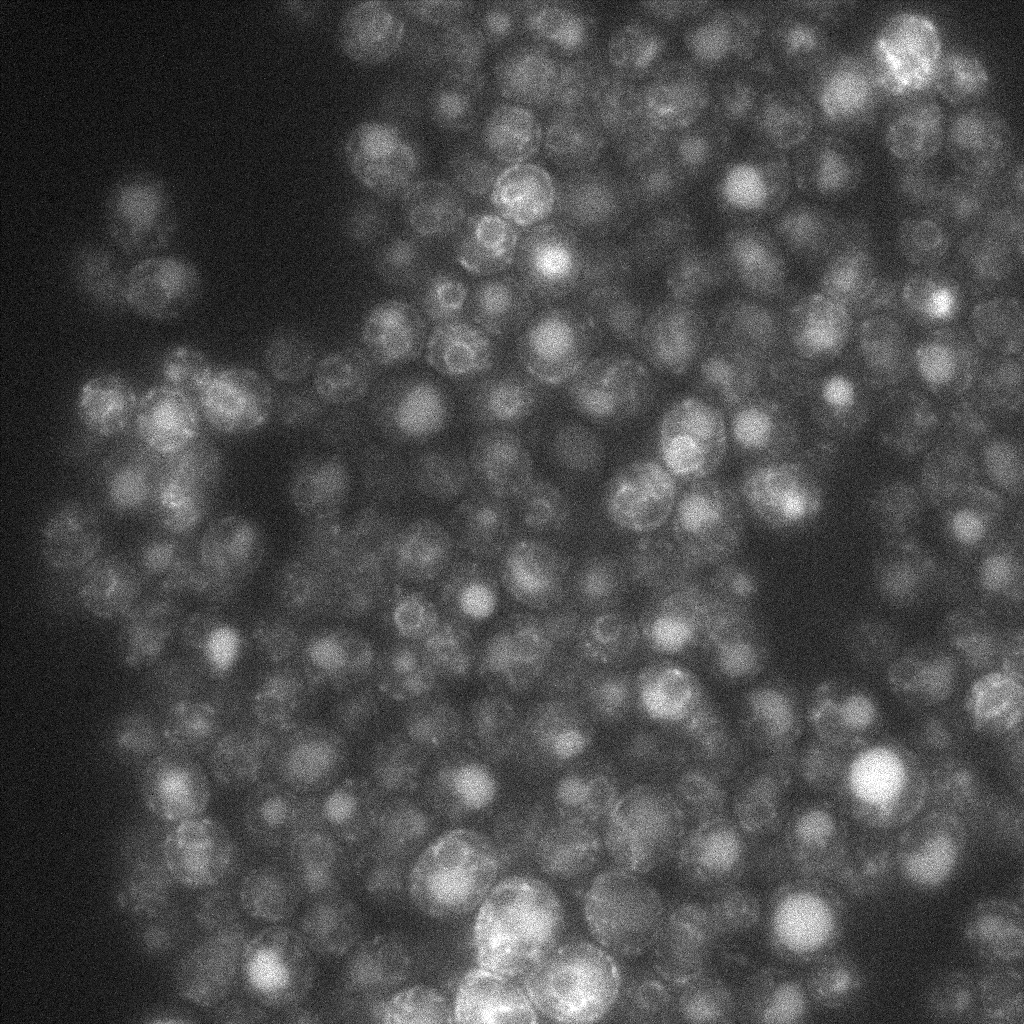

Supplement: Supplementary file 3 — Source data Fig. 2 [file 44318_2024_355_MOESM3_ESM.zip › Figure 2/2F/C2-Snap-1625 (Lro1*-mCh EXP).tif]

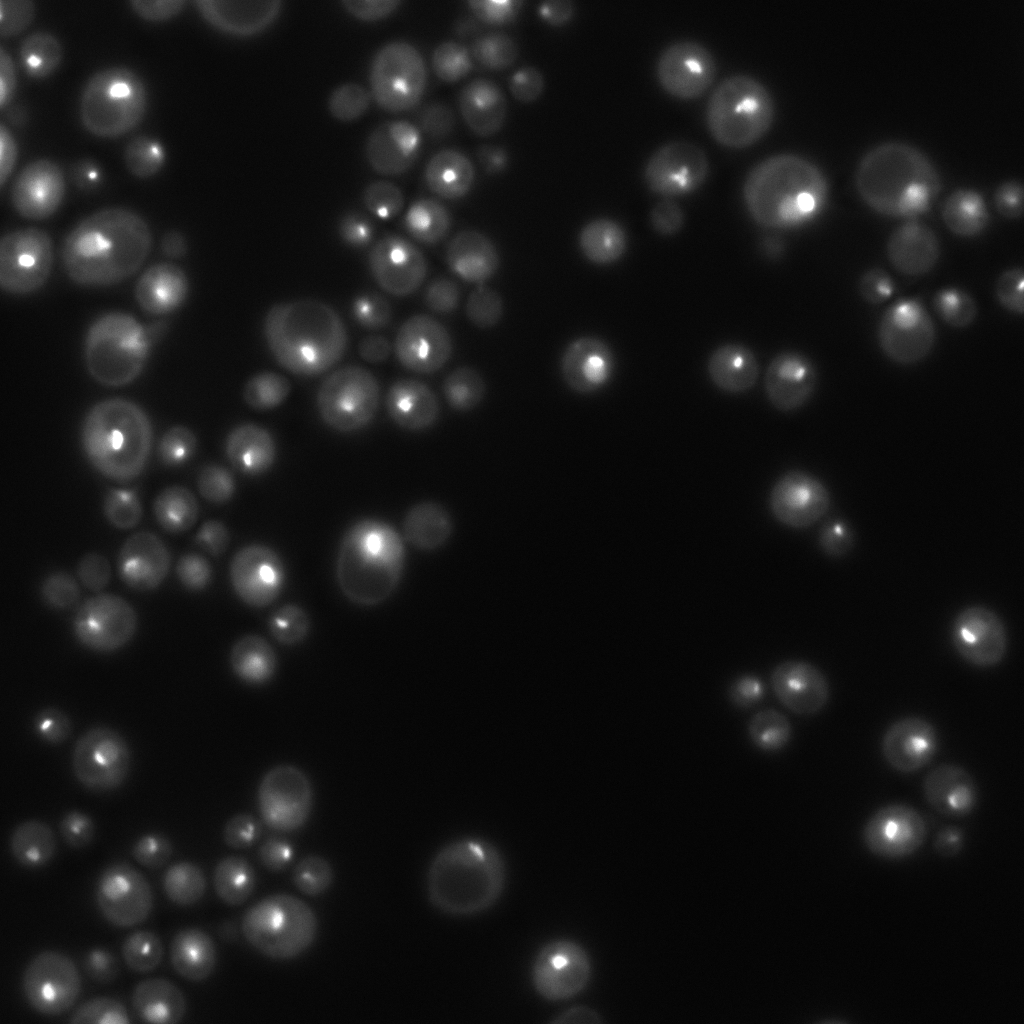

Supplement: Supplementary file 3 — Source data Fig. 2 [file 44318_2024_355_MOESM3_ESM.zip › Figure 2/2F/C1-Snap-68 (Nsr1-GFP PDS).tif]

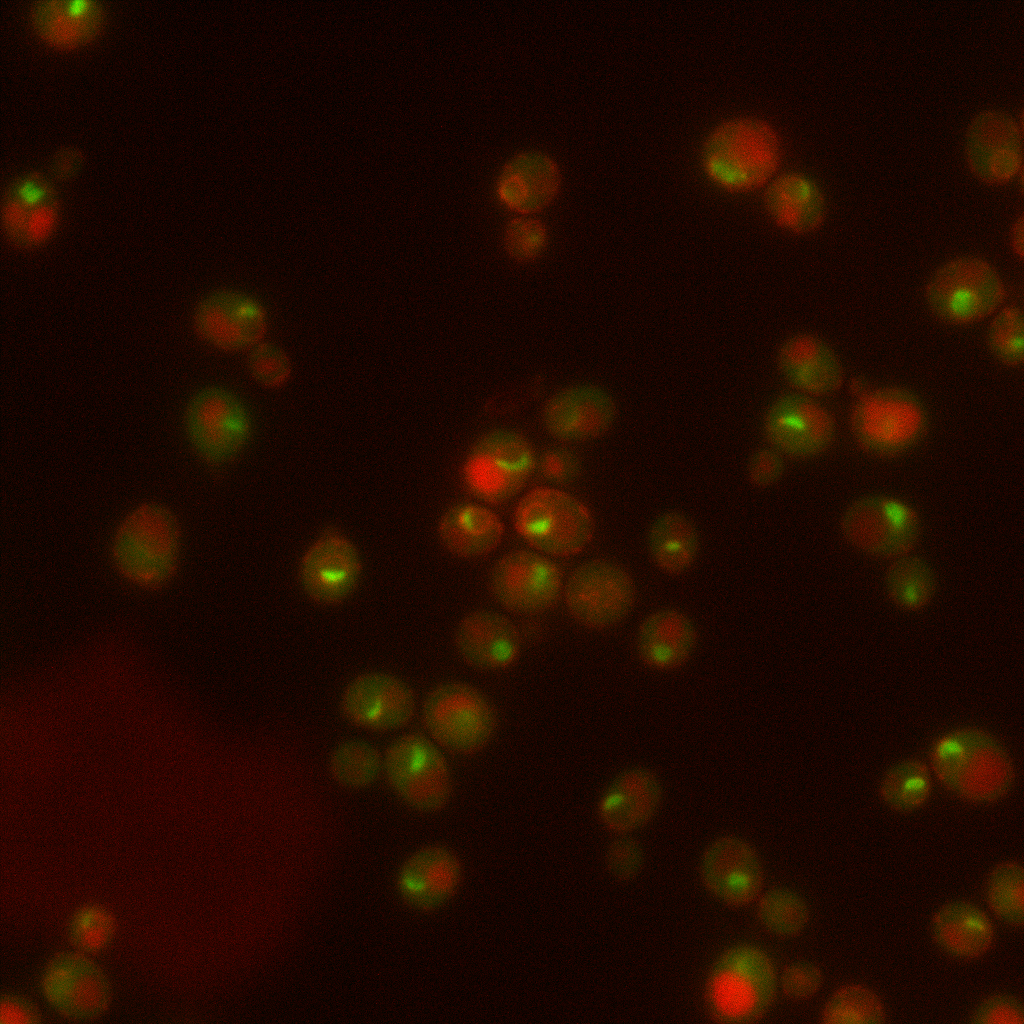

Supplement: Supplementary file 3 — Source data Fig. 2 [file 44318_2024_355_MOESM3_ESM.zip › Figure 2/2F/Merge (Lro1-mCh EXP).tif]

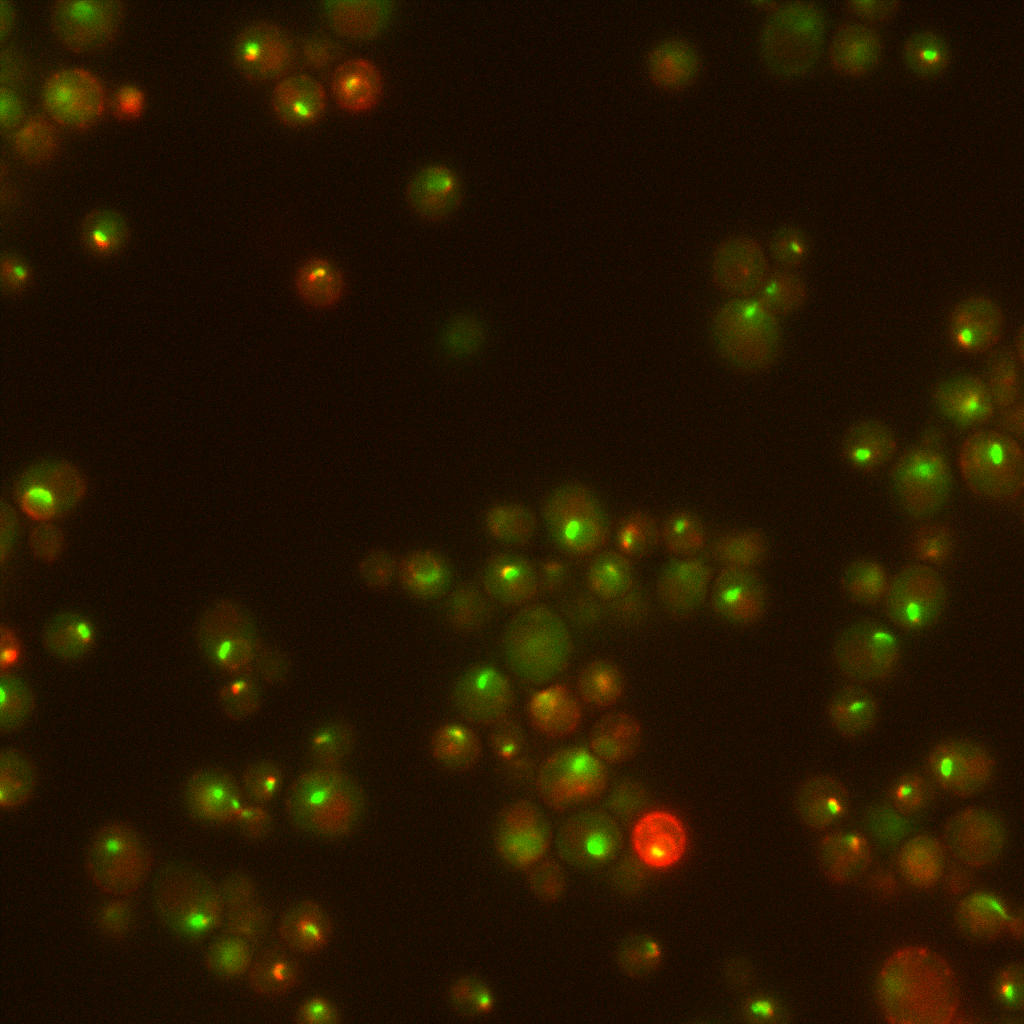

Supplement: Supplementary file 3 — Source data Fig. 2 [file 44318_2024_355_MOESM3_ESM.zip › Figure 2/2F/Merge (Lro1-mCh PDS).tif]

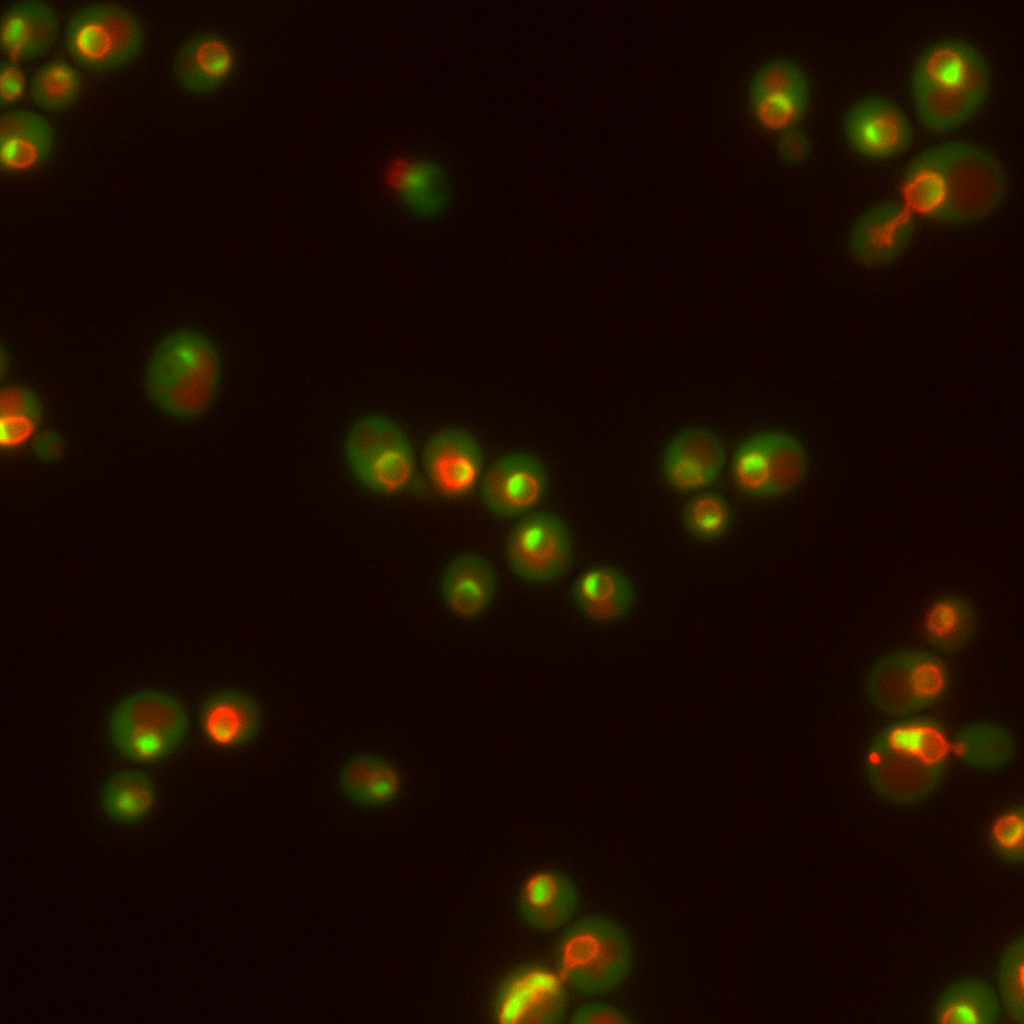

Supplement: Supplementary file 3 — Source data Fig. 2 [file 44318_2024_355_MOESM3_ESM.zip › Figure 2/2F/Merge (H1-Lro1*-mCh EXP).tif]

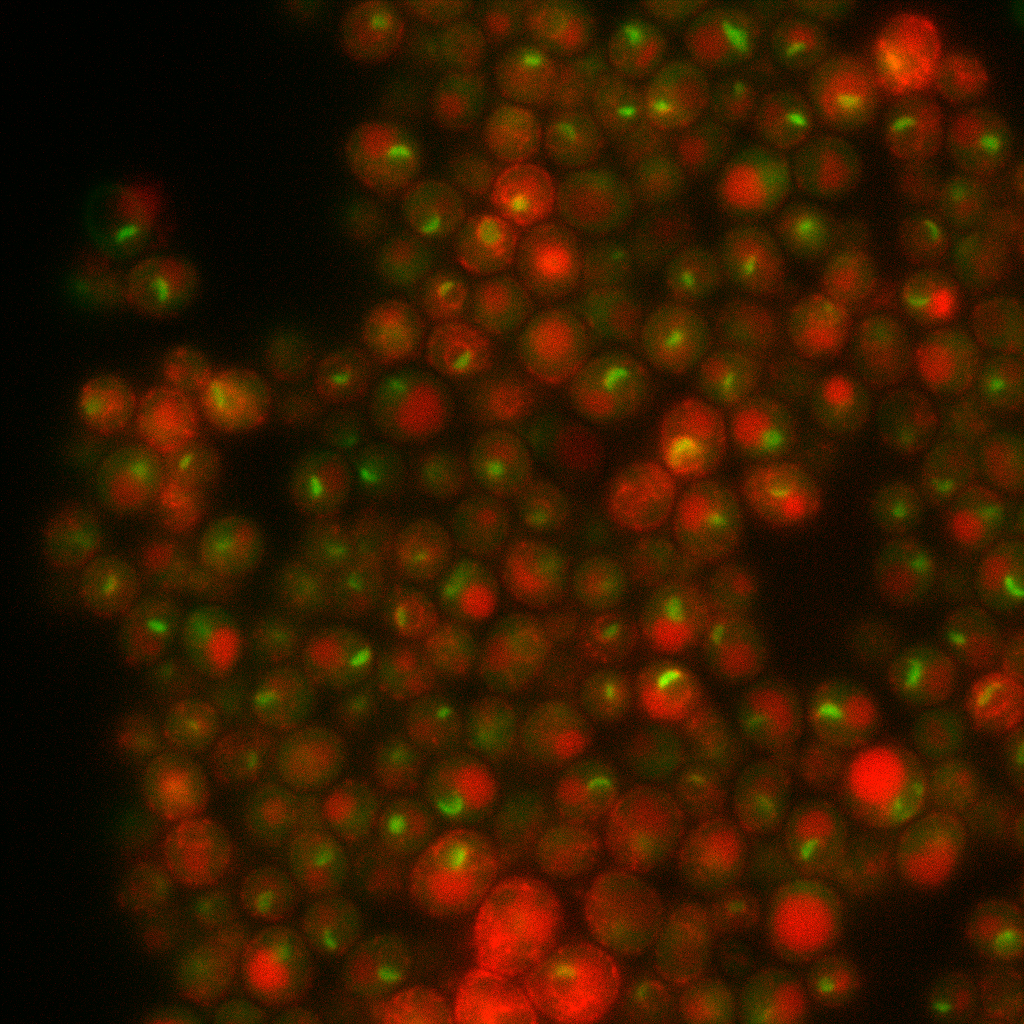

Supplement: Supplementary file 3 — Source data Fig. 2 [file 44318_2024_355_MOESM3_ESM.zip › Figure 2/2F/Merge (Lro1*-mCh EXP).tif]

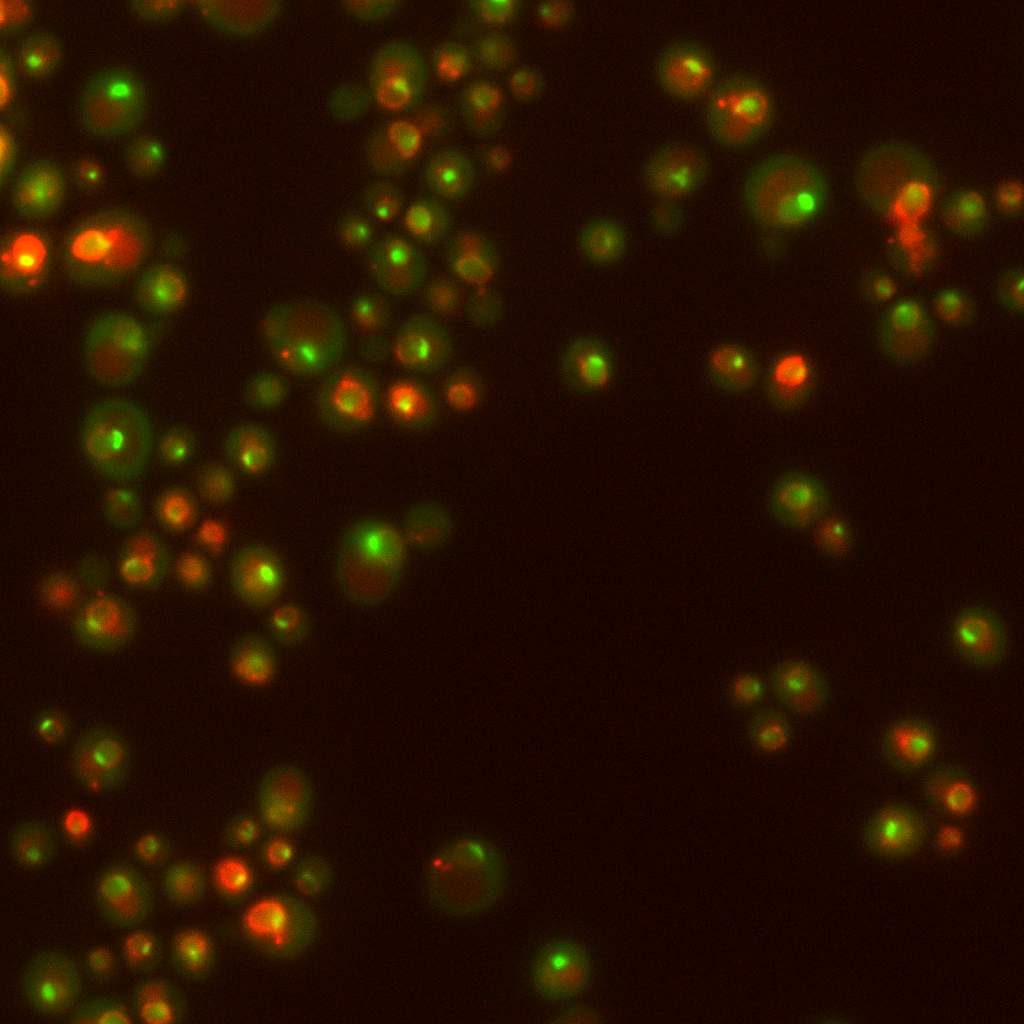

Supplement: Supplementary file 3 — Source data Fig. 2 [file 44318_2024_355_MOESM3_ESM.zip › Figure 2/2F/Merge (H1-Lro1*-mCh PDS).tif]

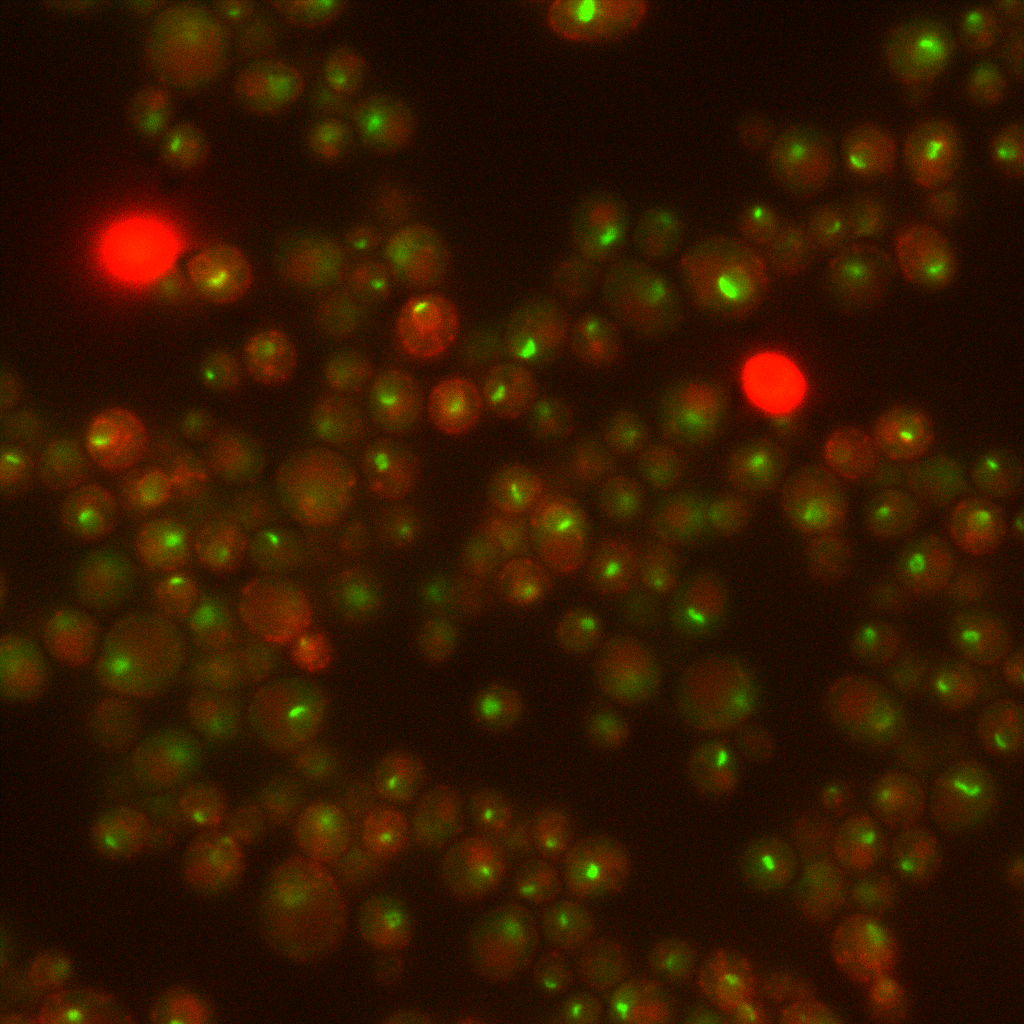

Supplement: Supplementary file 3 — Source data Fig. 2 [file 44318_2024_355_MOESM3_ESM.zip › Figure 2/2F/Merge (Lro1*-mCh PDS).tif]

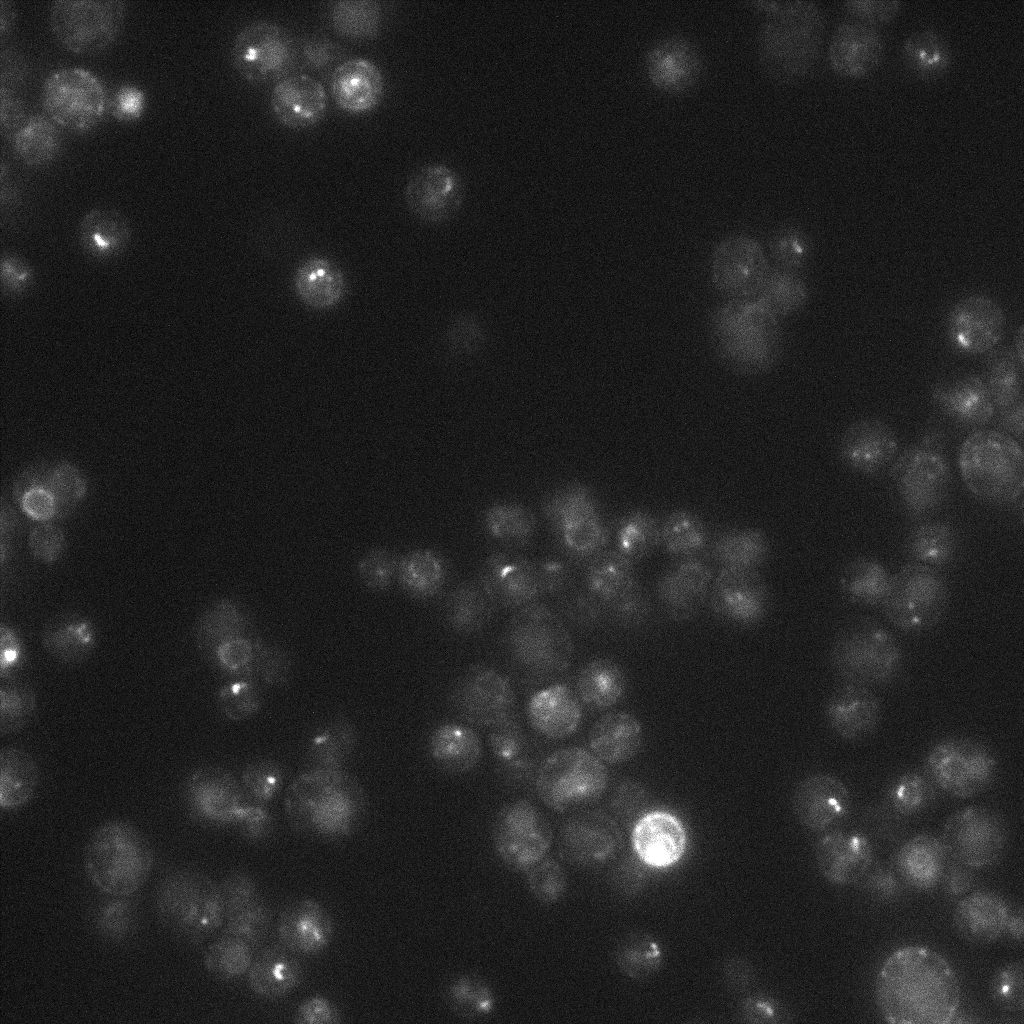

Supplement: Supplementary file 3 — Source data Fig. 2 [file 44318_2024_355_MOESM3_ESM.zip › Figure 2/2F/C2-Snap-1650 (Lro1-mCh PDS).tif]

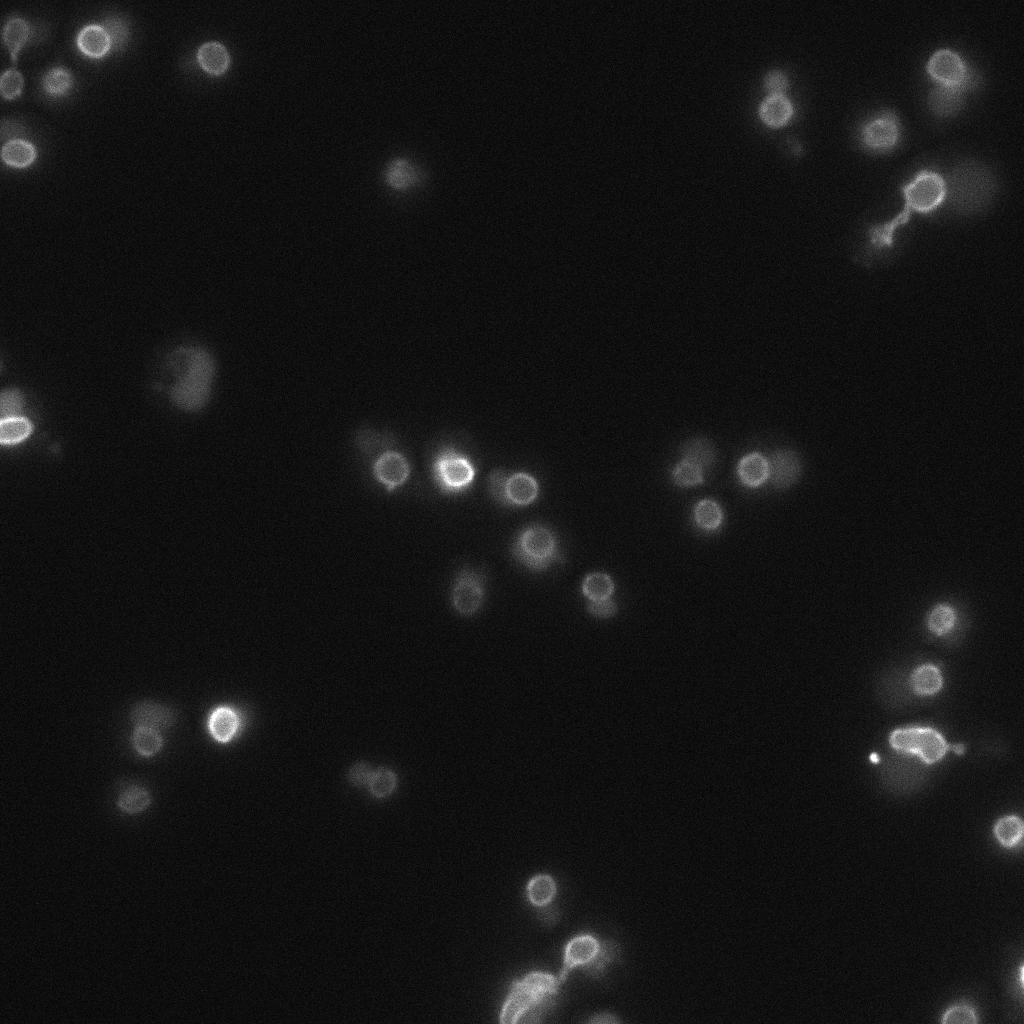

Supplement: Supplementary file 3 — Source data Fig. 2 [file 44318_2024_355_MOESM3_ESM.zip › Figure 2/2F/C2-Snap-52 (H1-Lro1*-mCh EXP).tif]

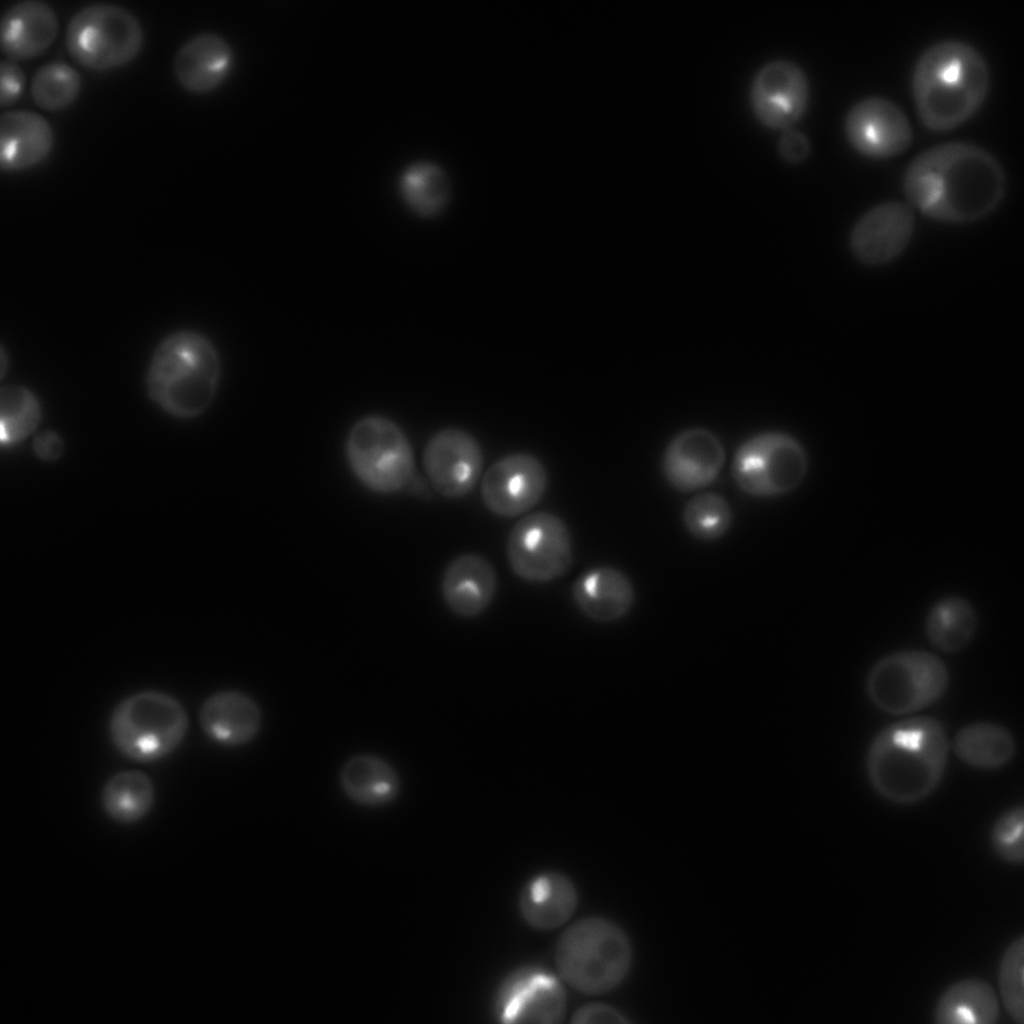

Supplement: Supplementary file 3 — Source data Fig. 2 [file 44318_2024_355_MOESM3_ESM.zip › Figure 2/2F/C1-Snap-52 (Nsr1-GFP EXP).tif]

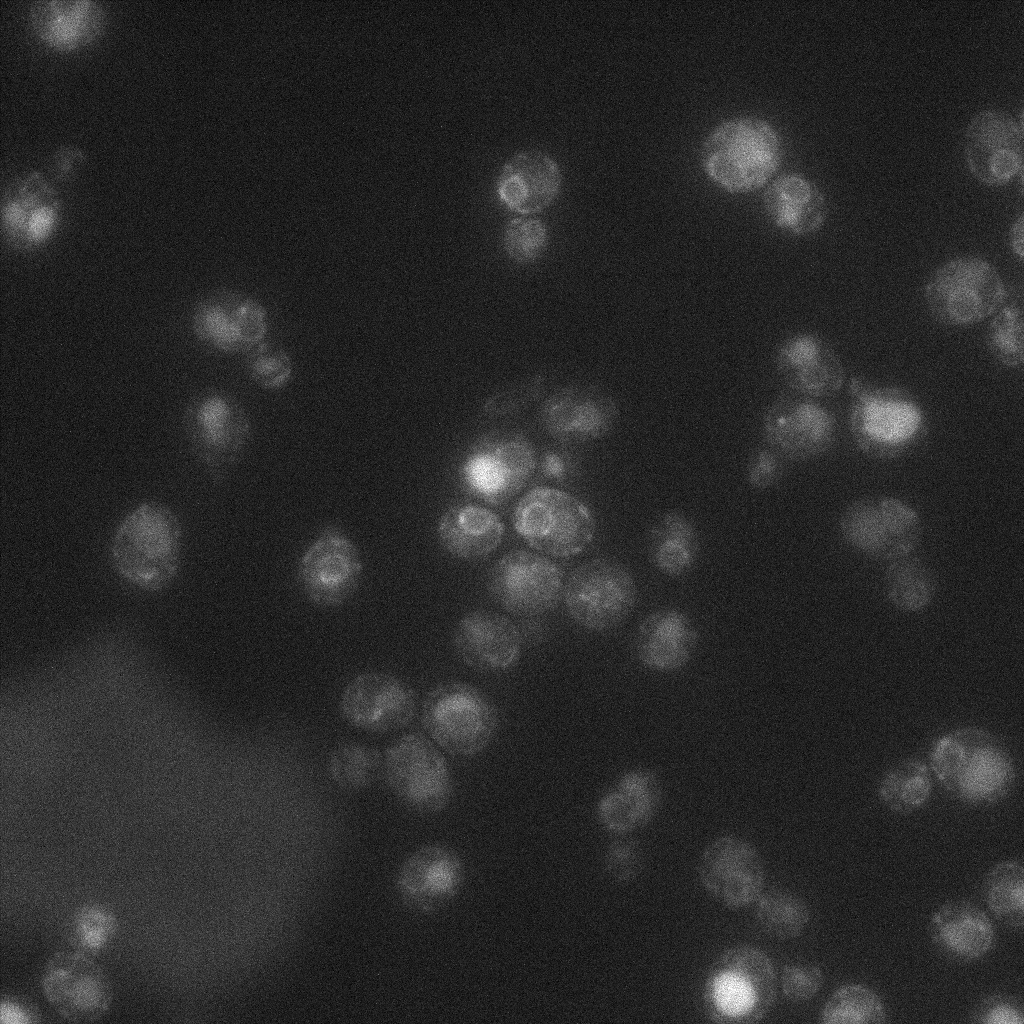

Supplement: Supplementary file 3 — Source data Fig. 2 [file 44318_2024_355_MOESM3_ESM.zip › Figure 2/2F/C2-Snap-1739 (Lro1-mCh EXP).tif]

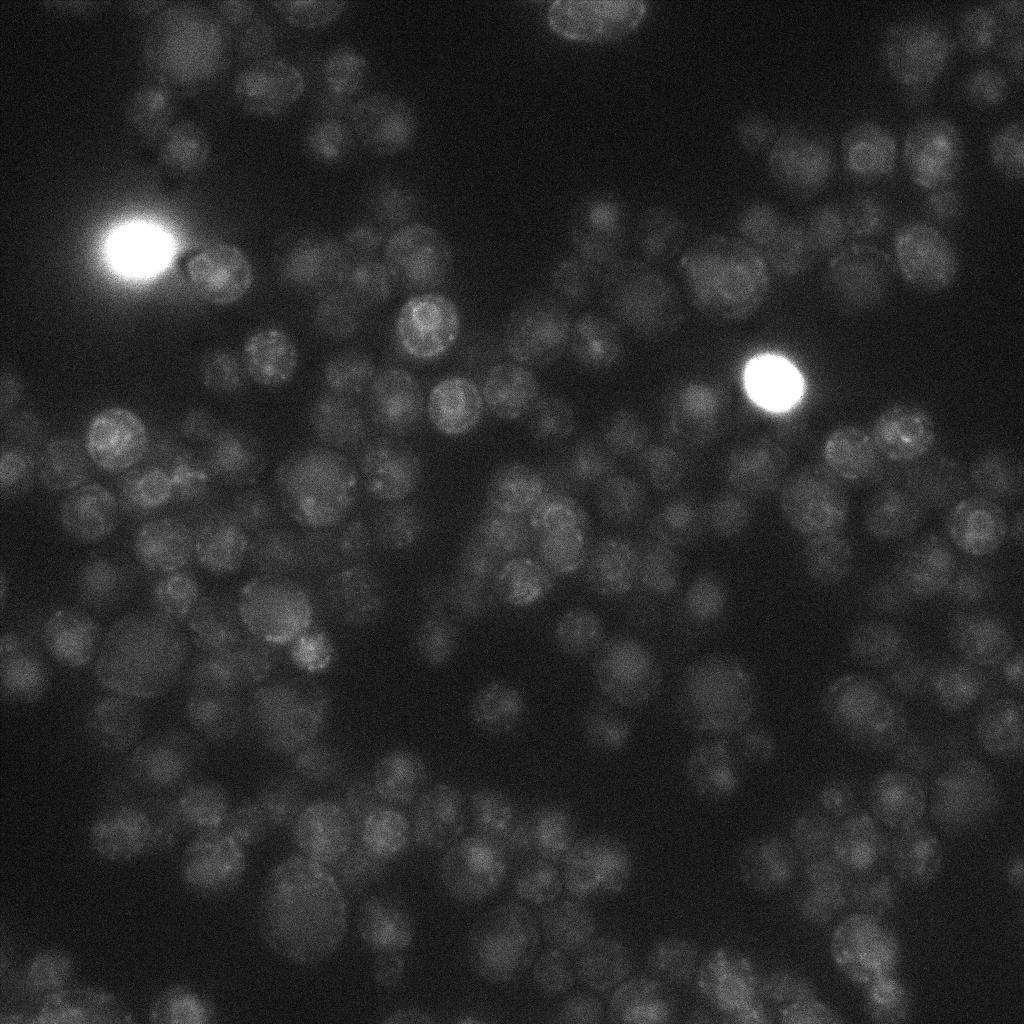

Supplement: Supplementary file 3 — Source data Fig. 2 [file 44318_2024_355_MOESM3_ESM.zip › Figure 2/2F/C2-Snap-1773 (Lro1*-mCh PDS).tif]

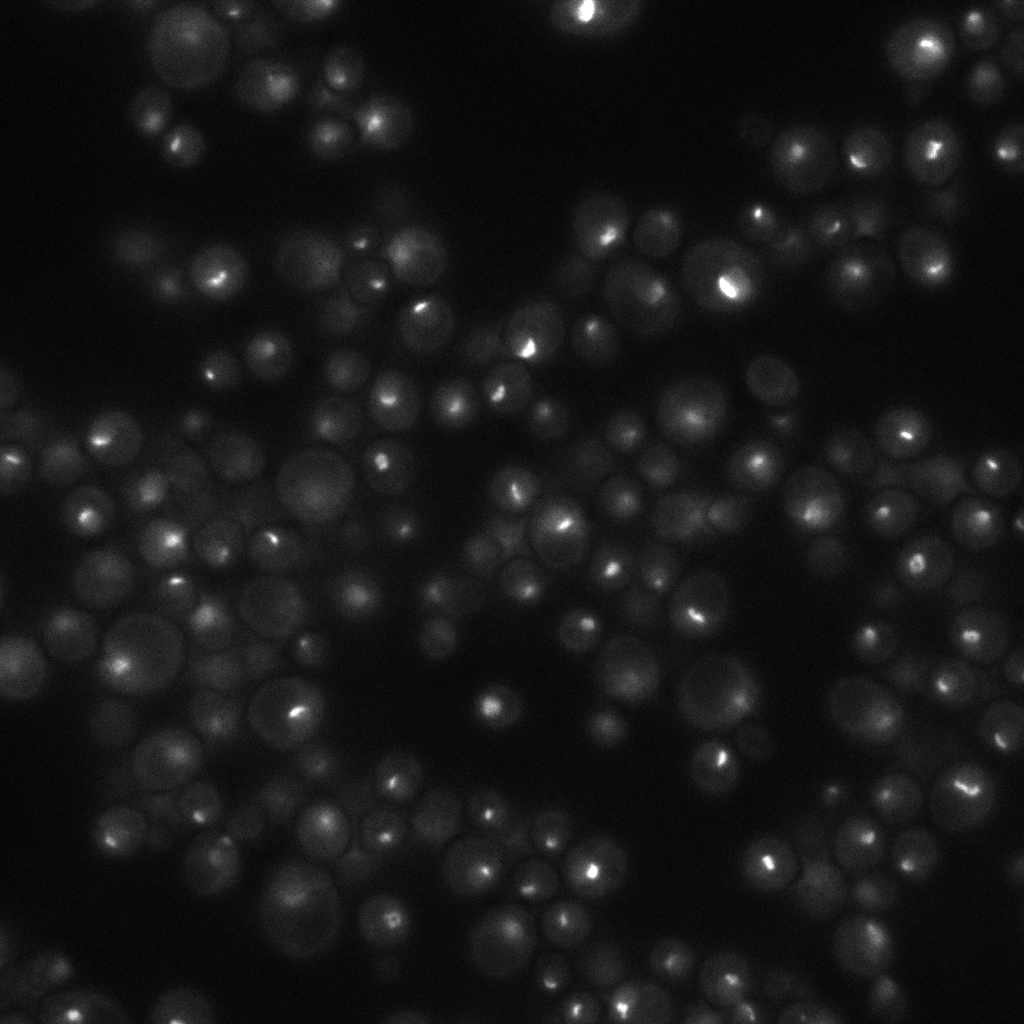

Supplement: Supplementary file 3 — Source data Fig. 2 [file 44318_2024_355_MOESM3_ESM.zip › Figure 2/2F/C1-Snap-1773 (Nsr1-GFP PDS).tif]

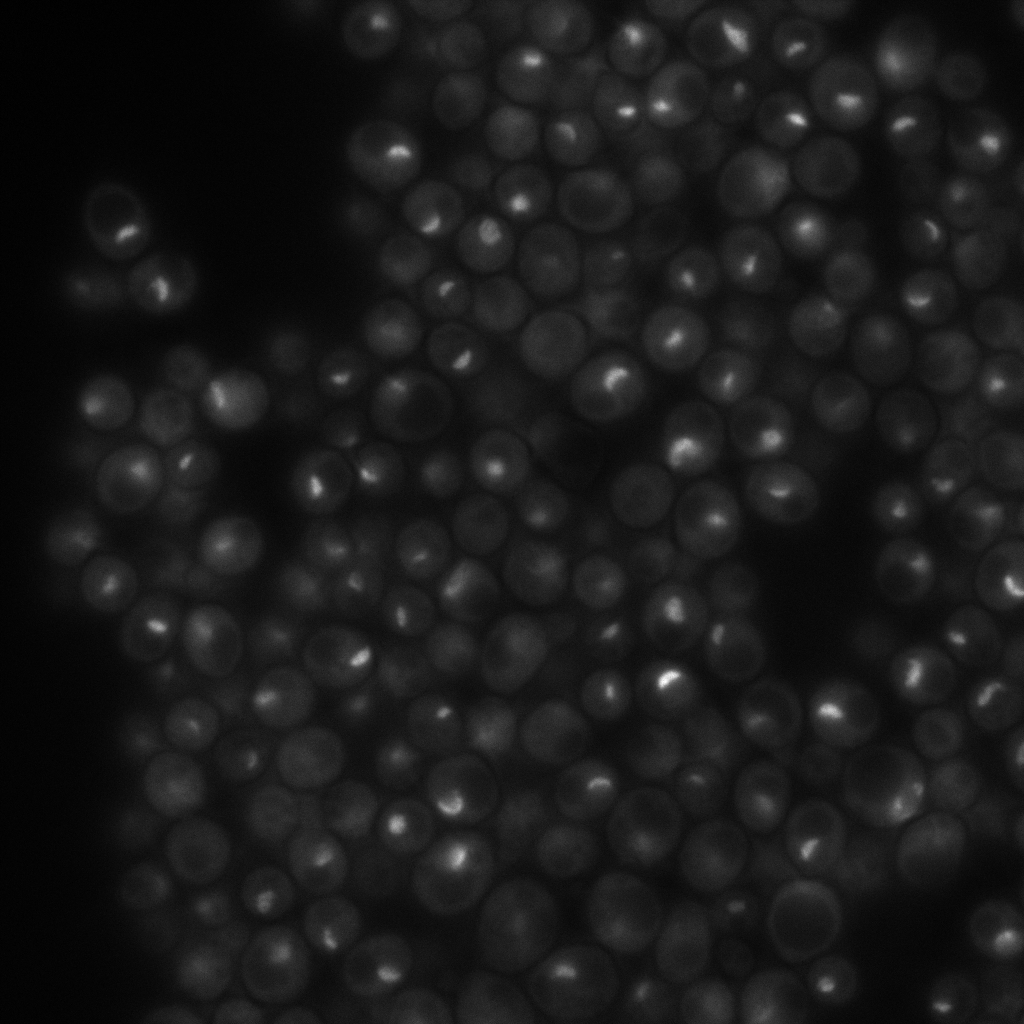

Supplement: Supplementary file 3 — Source data Fig. 2 [file 44318_2024_355_MOESM3_ESM.zip › Figure 2/2F/C1-Snap-1625 (Nsr1-GFP EXP).tif]

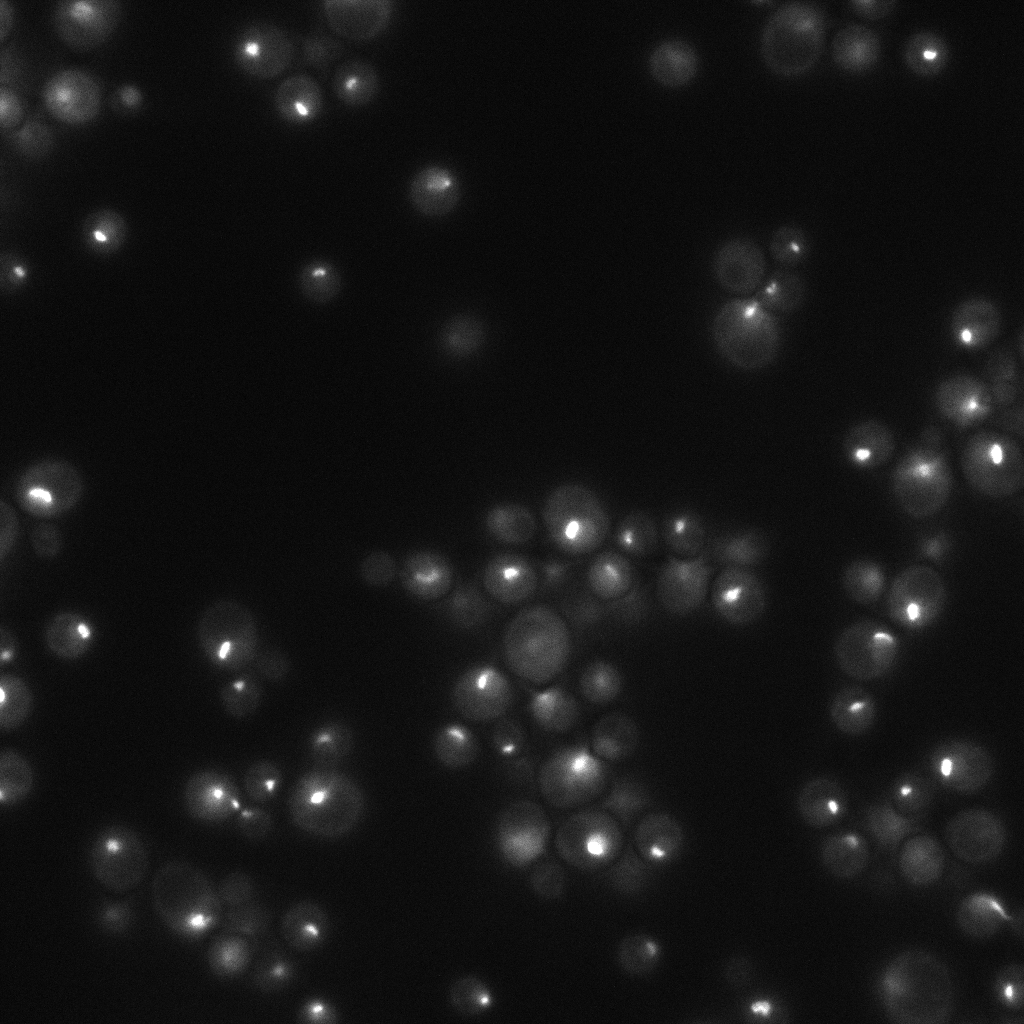

Supplement: Supplementary file 3 — Source data Fig. 2 [file 44318_2024_355_MOESM3_ESM.zip › Figure 2/2F/C1-Snap-1650 (Nsr1-GFP PDS).tif]

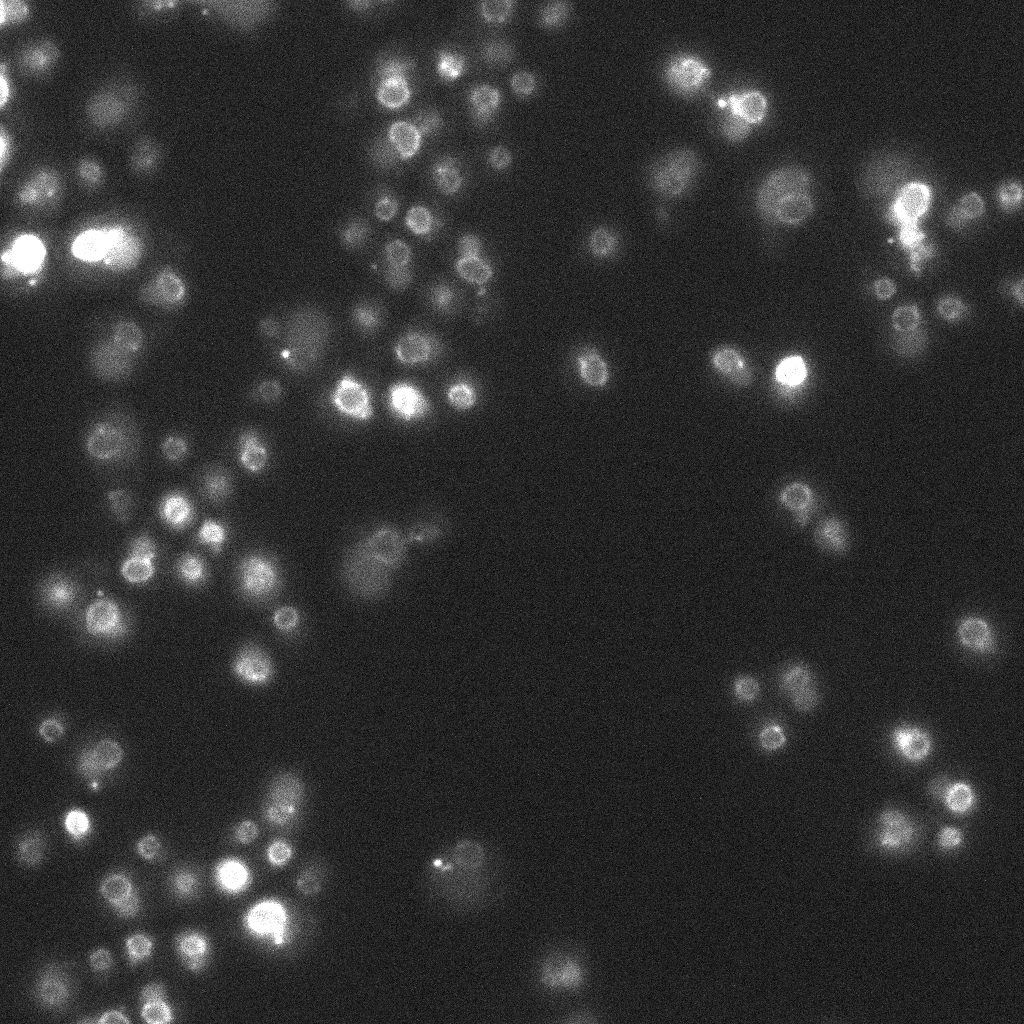

Supplement: Supplementary file 3 — Source data Fig. 2 [file 44318_2024_355_MOESM3_ESM.zip › Figure 2/2F/C2-Snap-68 (H1-Lro1*-mCh PDS).tif]

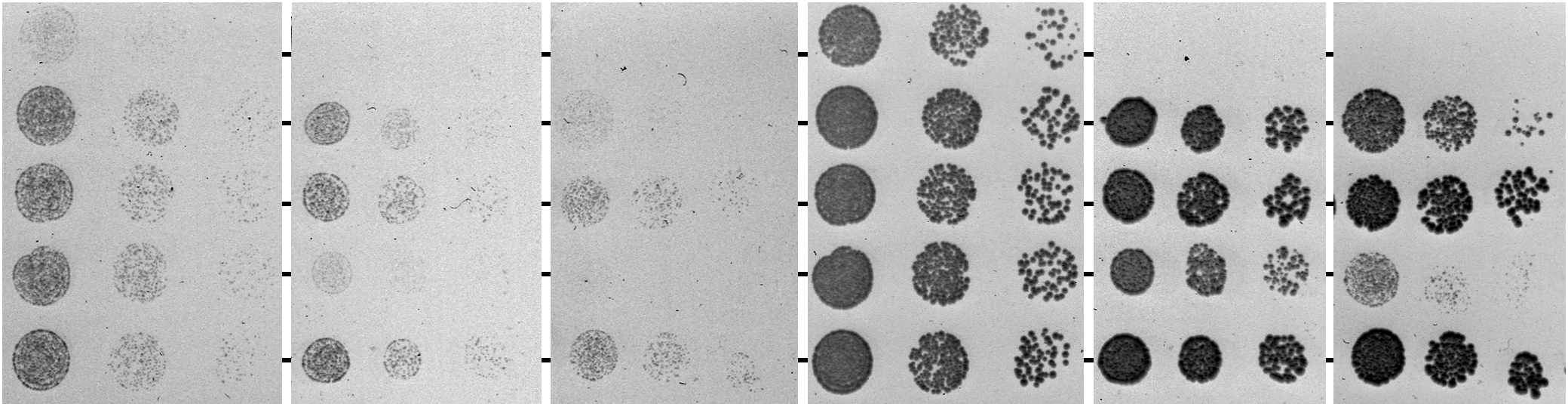

Supplement: Supplementary file 3 — Source data Fig. 2 [file 44318_2024_355_MOESM3_ESM.zip › Figure 2/2E/Spot growth assay (plus minus oleate).tif]

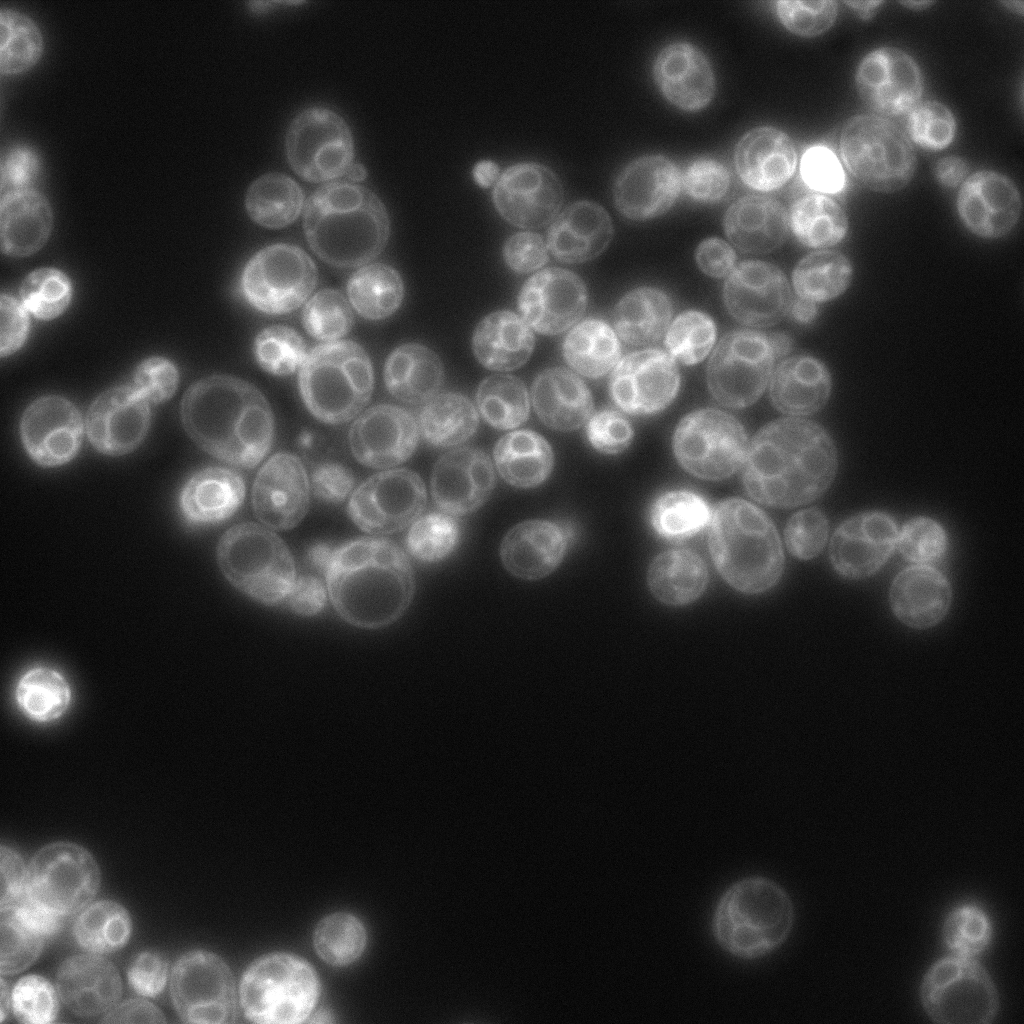

Supplement: Supplementary file 3 — Source data Fig. 2 [file 44318_2024_355_MOESM3_ESM.zip › Figure 2/2B/C1-Snap-1148-1(Lro1*[S324A]).tif]

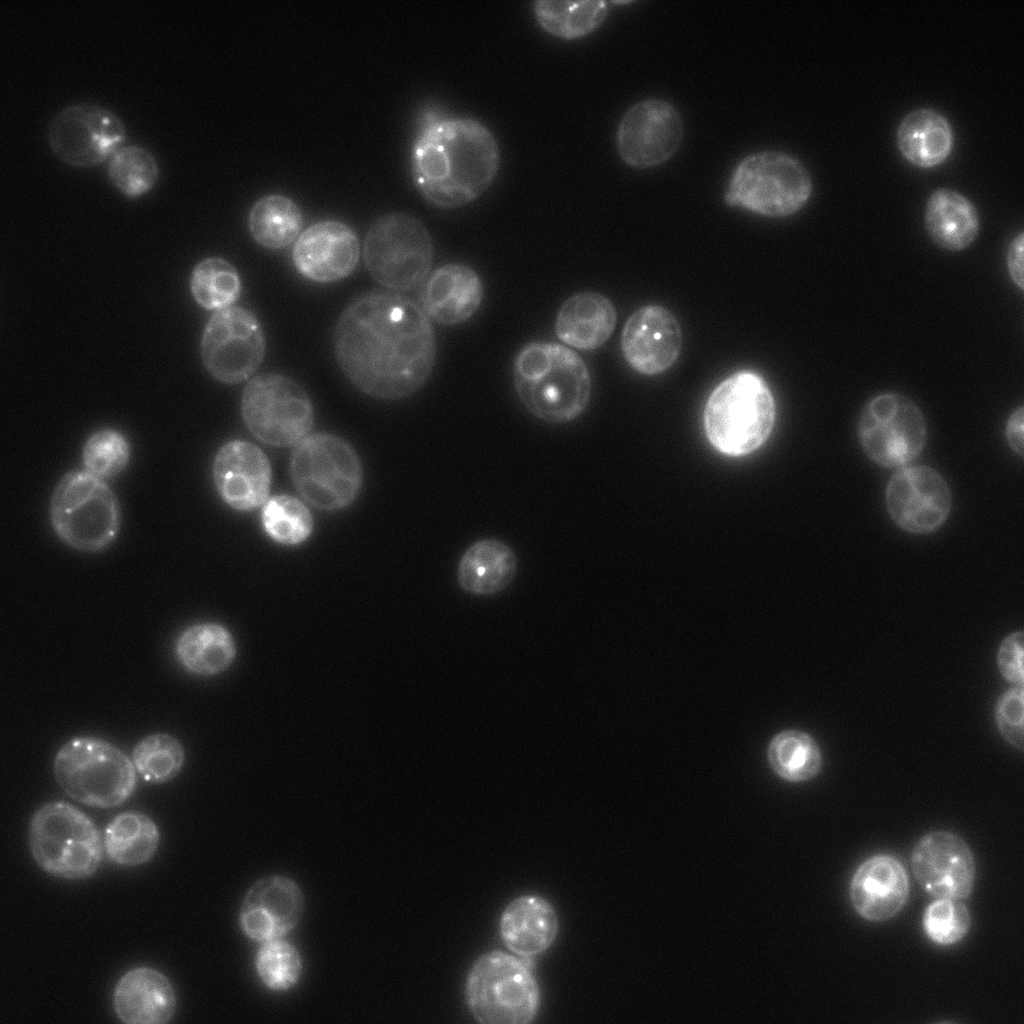

Supplement: Supplementary file 3 — Source data Fig. 2 [file 44318_2024_355_MOESM3_ESM.zip › Figure 2/2B/C1-Snap-1127-1 (Lro1-mCh).tif]

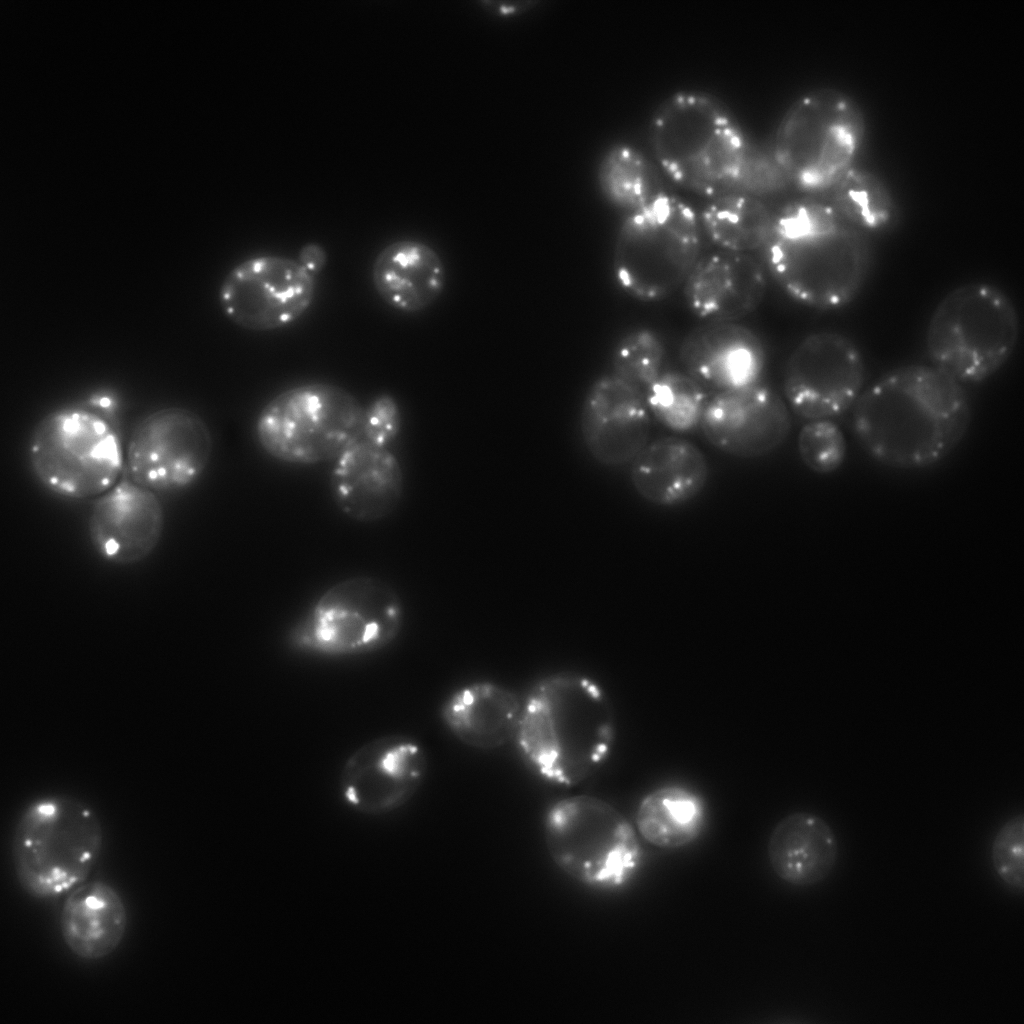

Supplement: Supplementary file 3 — Source data Fig. 2 [file 44318_2024_355_MOESM3_ESM.zip › Figure 2/2B/C1-Snap-1143-1(Lro1*-mCh).tif]

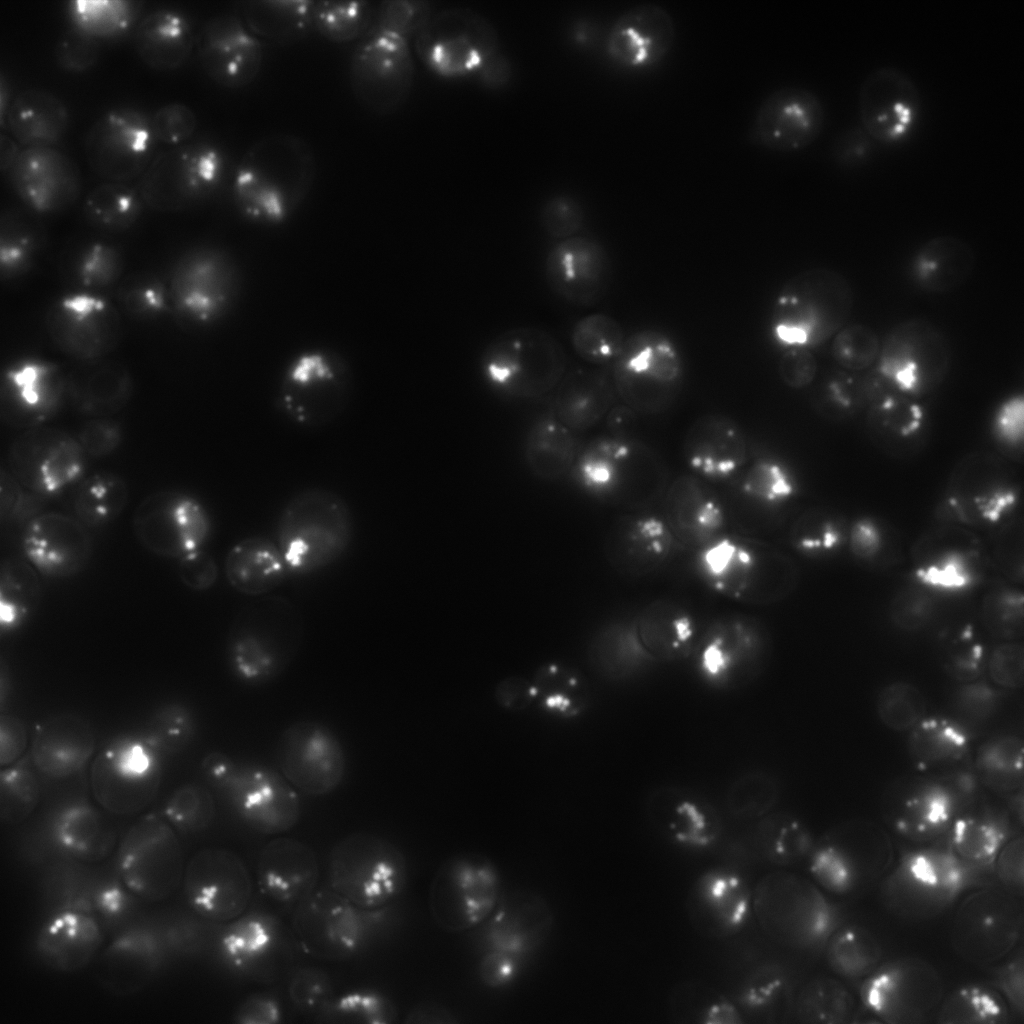

Supplement: Supplementary file 3 — Source data Fig. 2 [file 44318_2024_355_MOESM3_ESM.zip › Figure 2/2B/C1-Snap-1132-1(H1-Lro1*-mCh).tif]

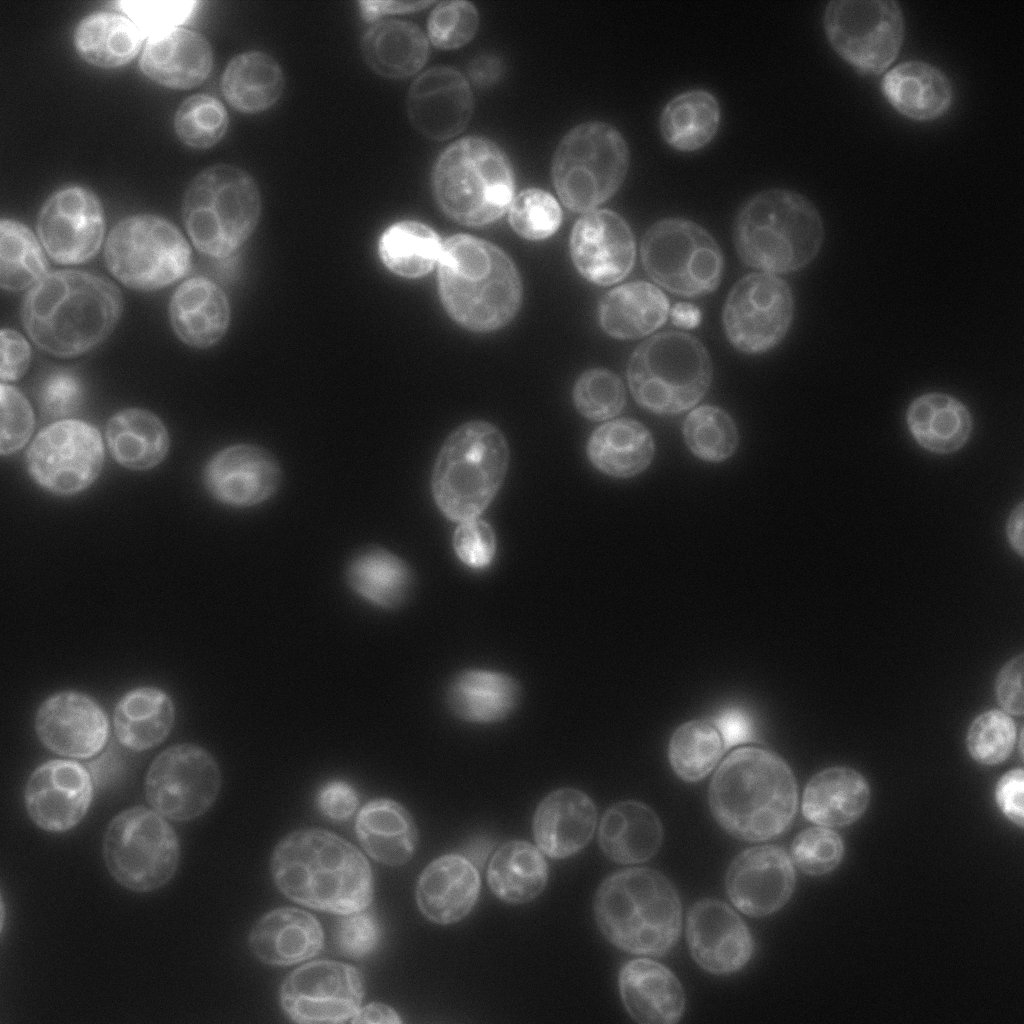

Supplement: Supplementary file 3 — Source data Fig. 2 [file 44318_2024_355_MOESM3_ESM.zip › Figure 2/2B/C1-Snap-1118-1 (vector).tif]

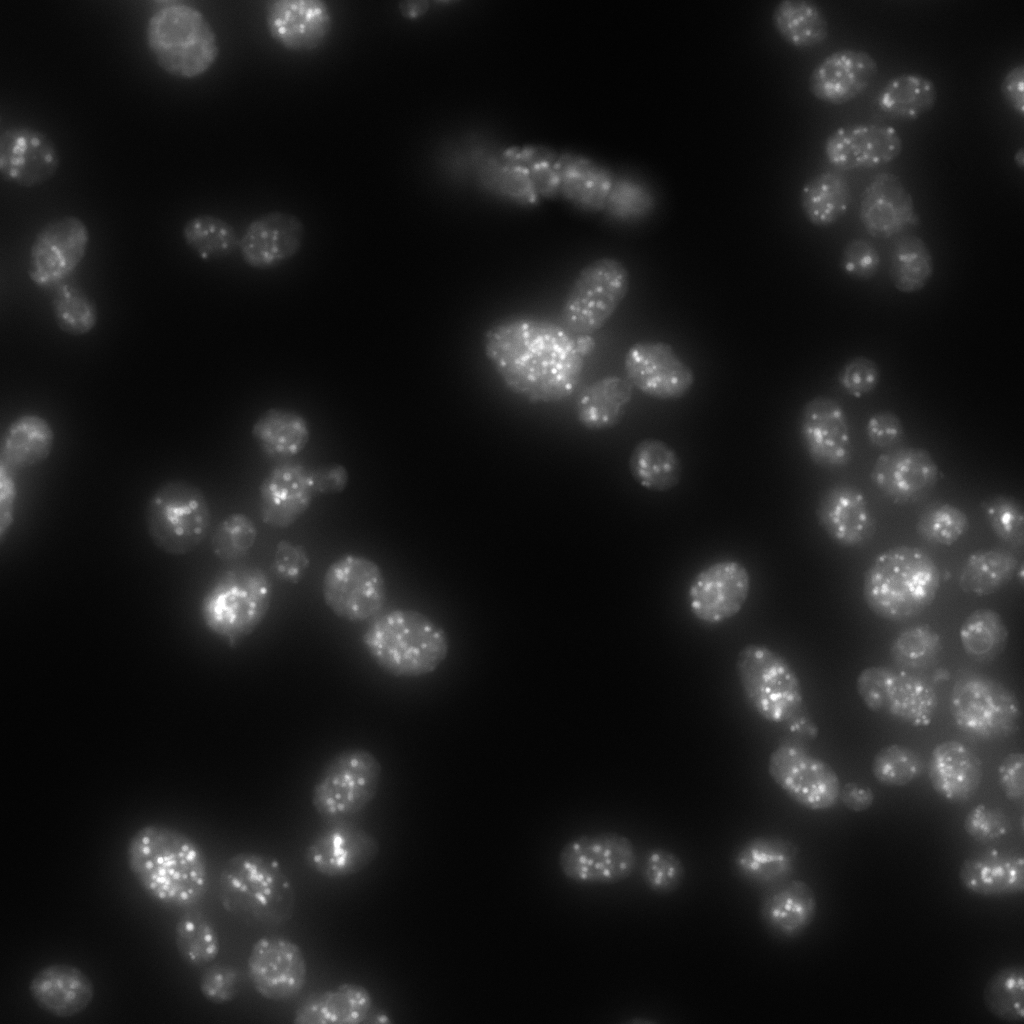

Supplement: Supplementary file 4 — Source data Fig. 3 [file 44318_2024_355_MOESM4_ESM.zip › Figure 3/3B/C1-MAX_Experiment-28 (GAL-Lro1* S324A MDH).tif]

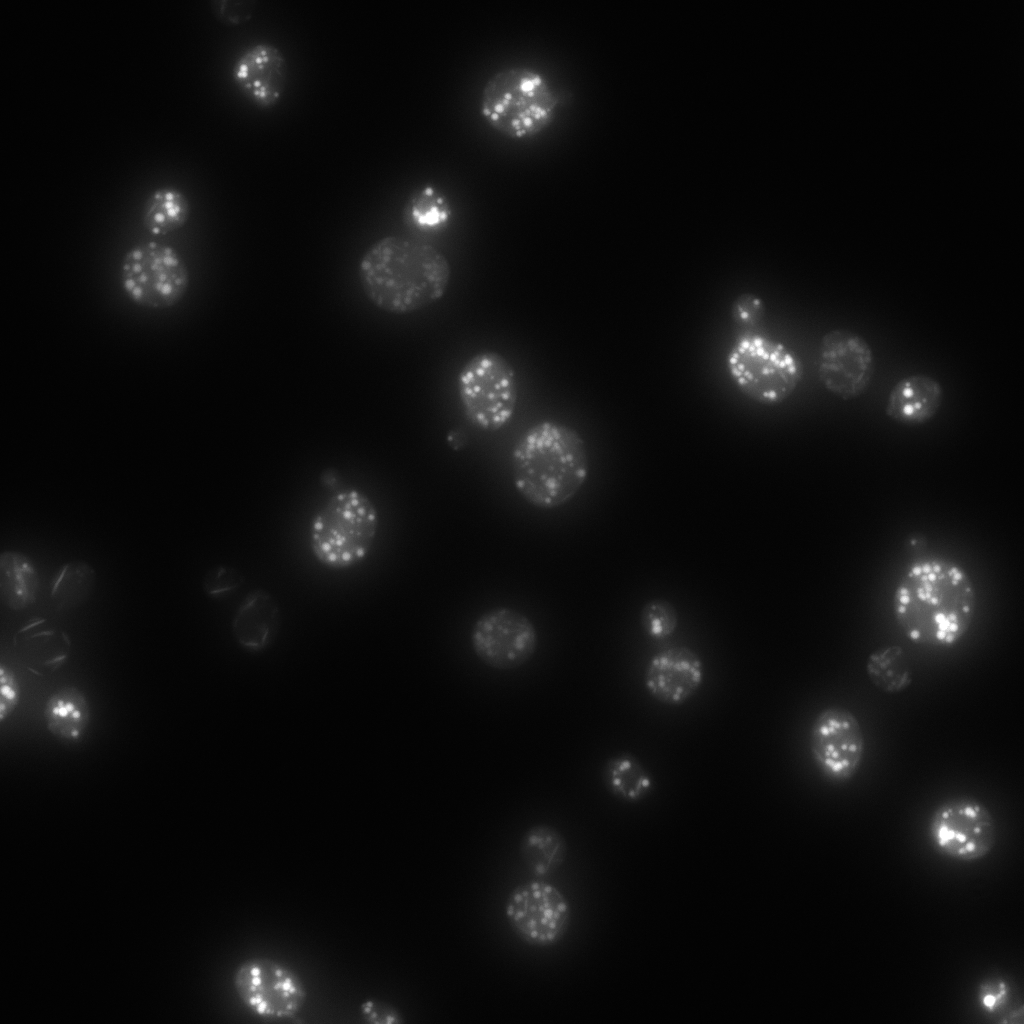

Supplement: Supplementary file 4 — Source data Fig. 3 [file 44318_2024_355_MOESM4_ESM.zip › Figure 3/3B/C3-MAX_Experiment-24 (GAL-Lro1* MDH).tif]

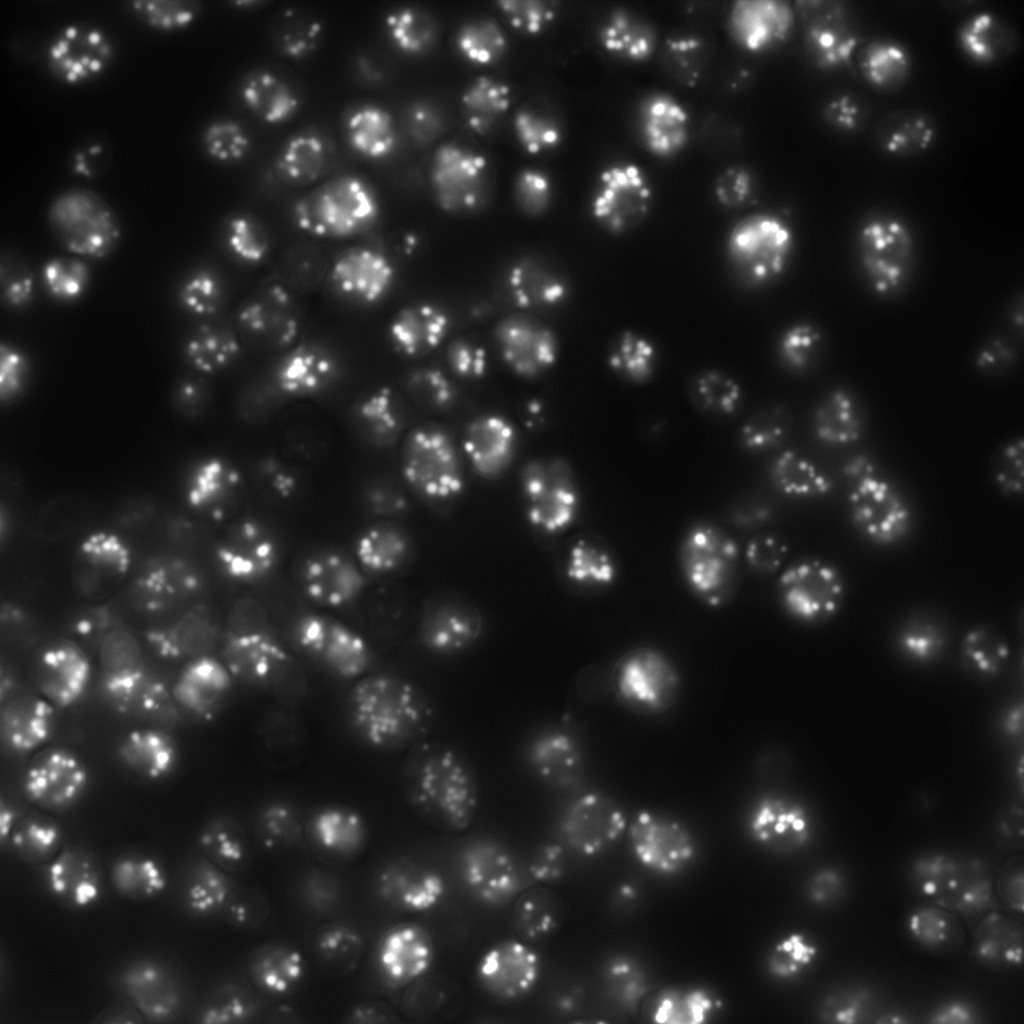

Supplement: Supplementary file 4 — Source data Fig. 3 [file 44318_2024_355_MOESM4_ESM.zip › Figure 3/3B/C1-Snap-1892 (GAL-Lro1*).tif]

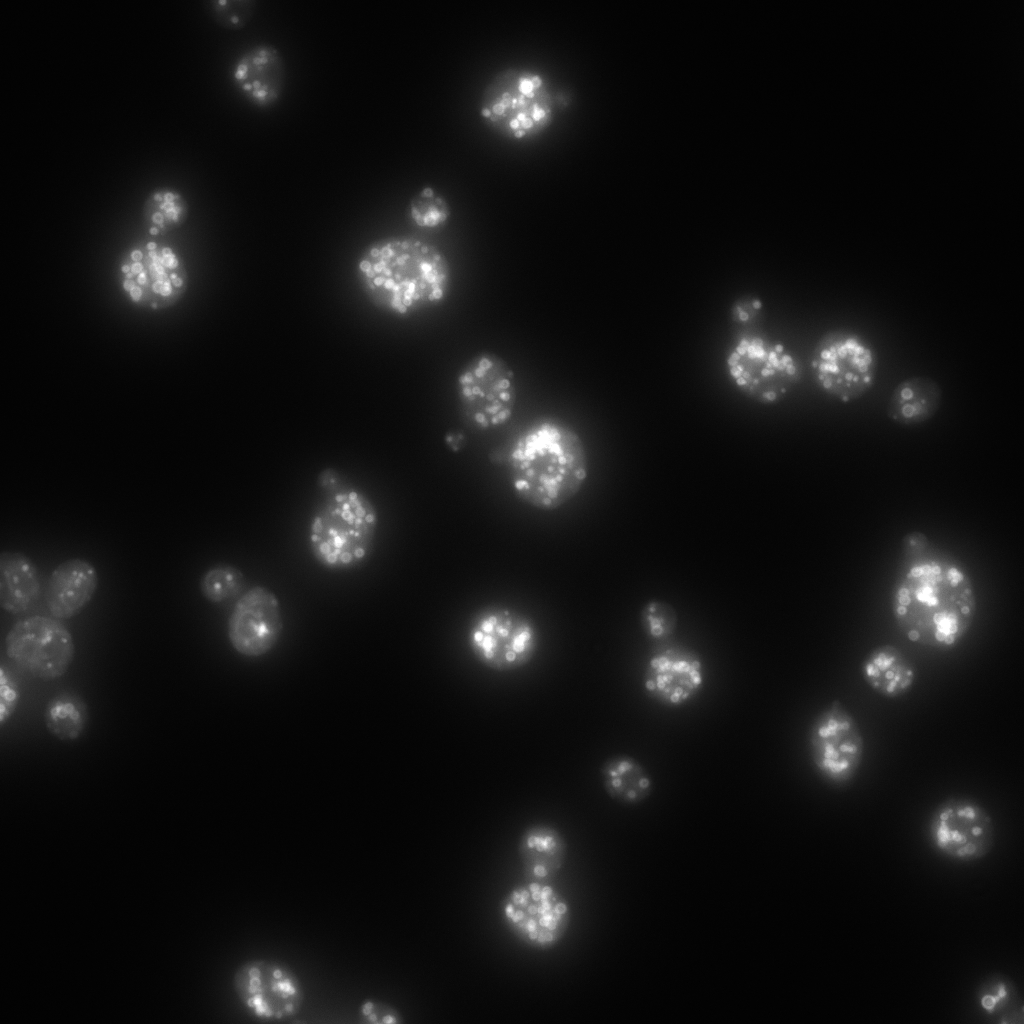

Supplement: Supplementary file 4 — Source data Fig. 3 [file 44318_2024_355_MOESM4_ESM.zip › Figure 3/3B/C1-MAX_Experiment-24 (GAL-Lro1* FAA4mNG).tif]

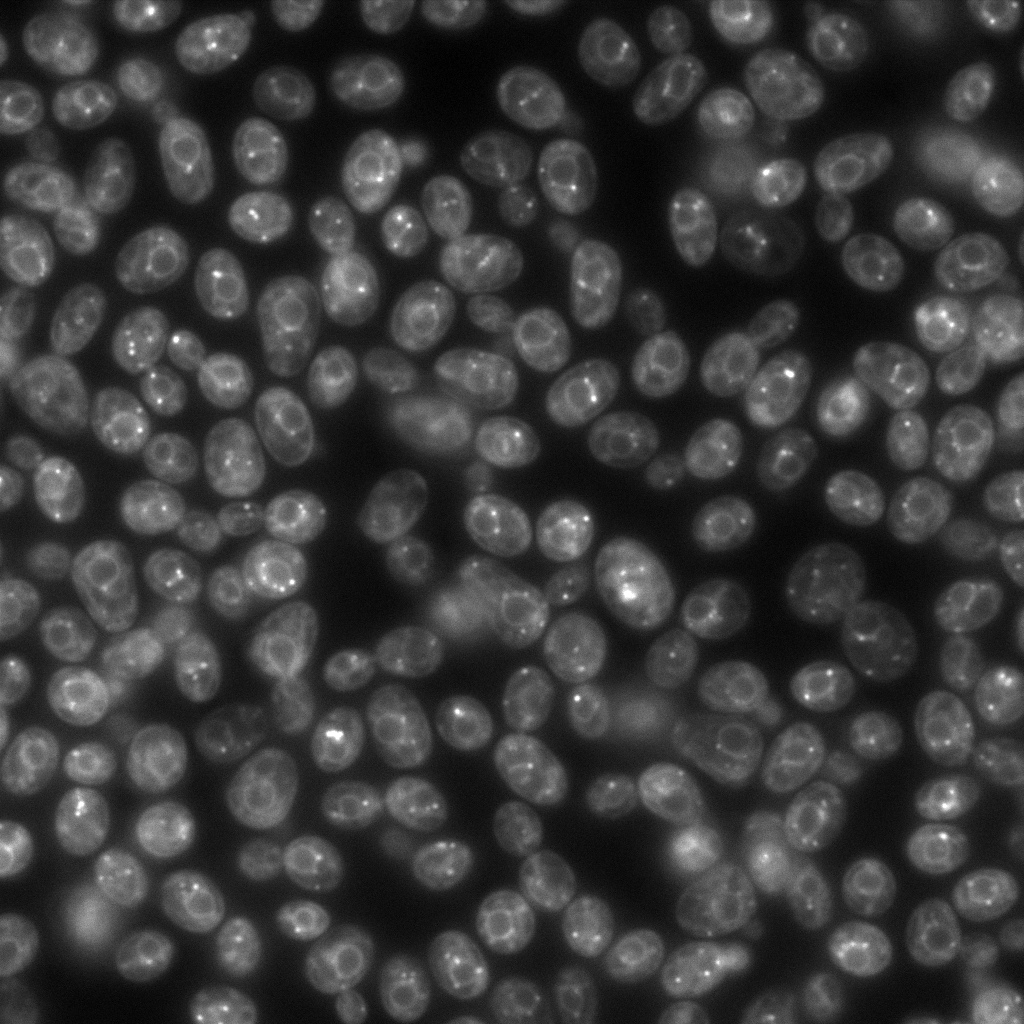

Supplement: Supplementary file 4 — Source data Fig. 3 [file 44318_2024_355_MOESM4_ESM.zip › Figure 3/3B/C1-Snap-1887(vector).tif]

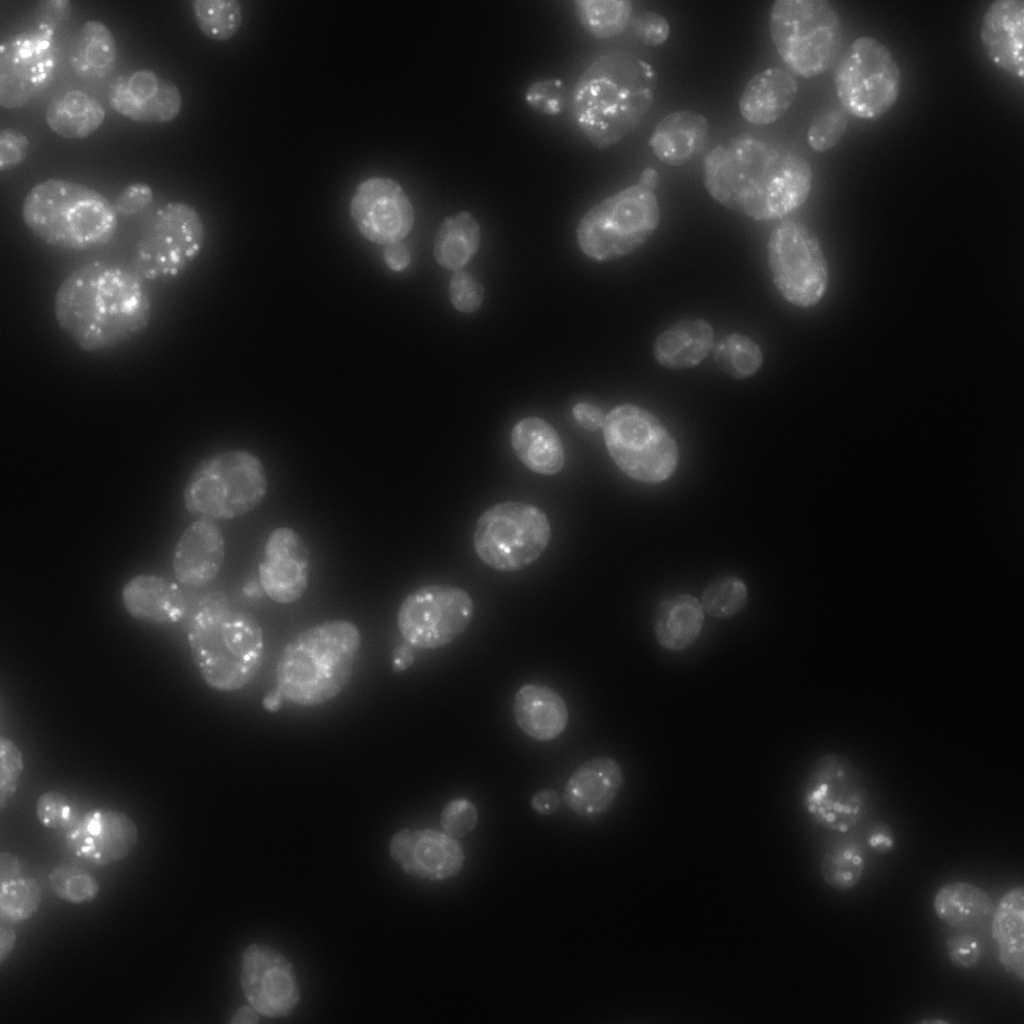

Supplement: Supplementary file 4 — Source data Fig. 3 [file 44318_2024_355_MOESM4_ESM.zip › Figure 3/3B/C1-MAX_Experiment16 (vector MDH).tif]

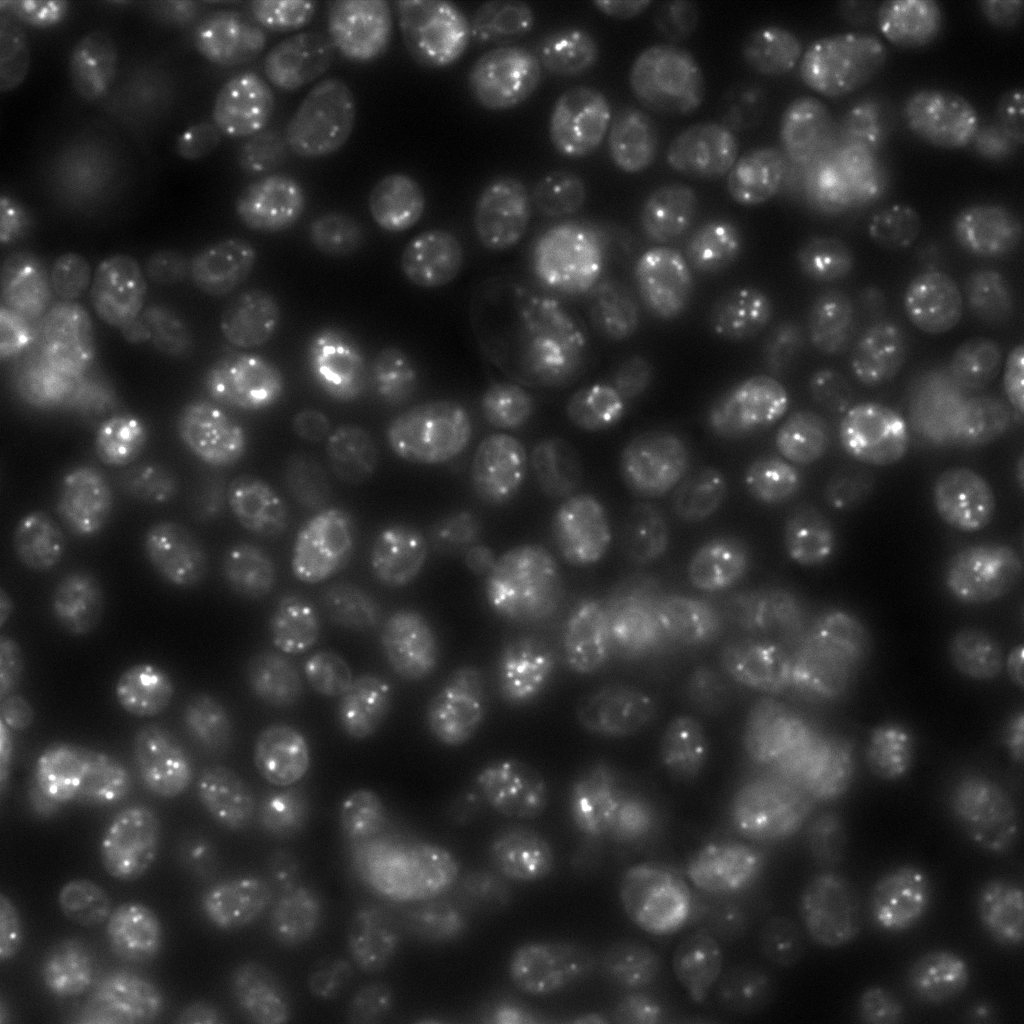

Supplement: Supplementary file 4 — Source data Fig. 3 [file 44318_2024_355_MOESM4_ESM.zip › Figure 3/3B/C1-Snap-1897(GAL-Lro1*-S324A).tif]

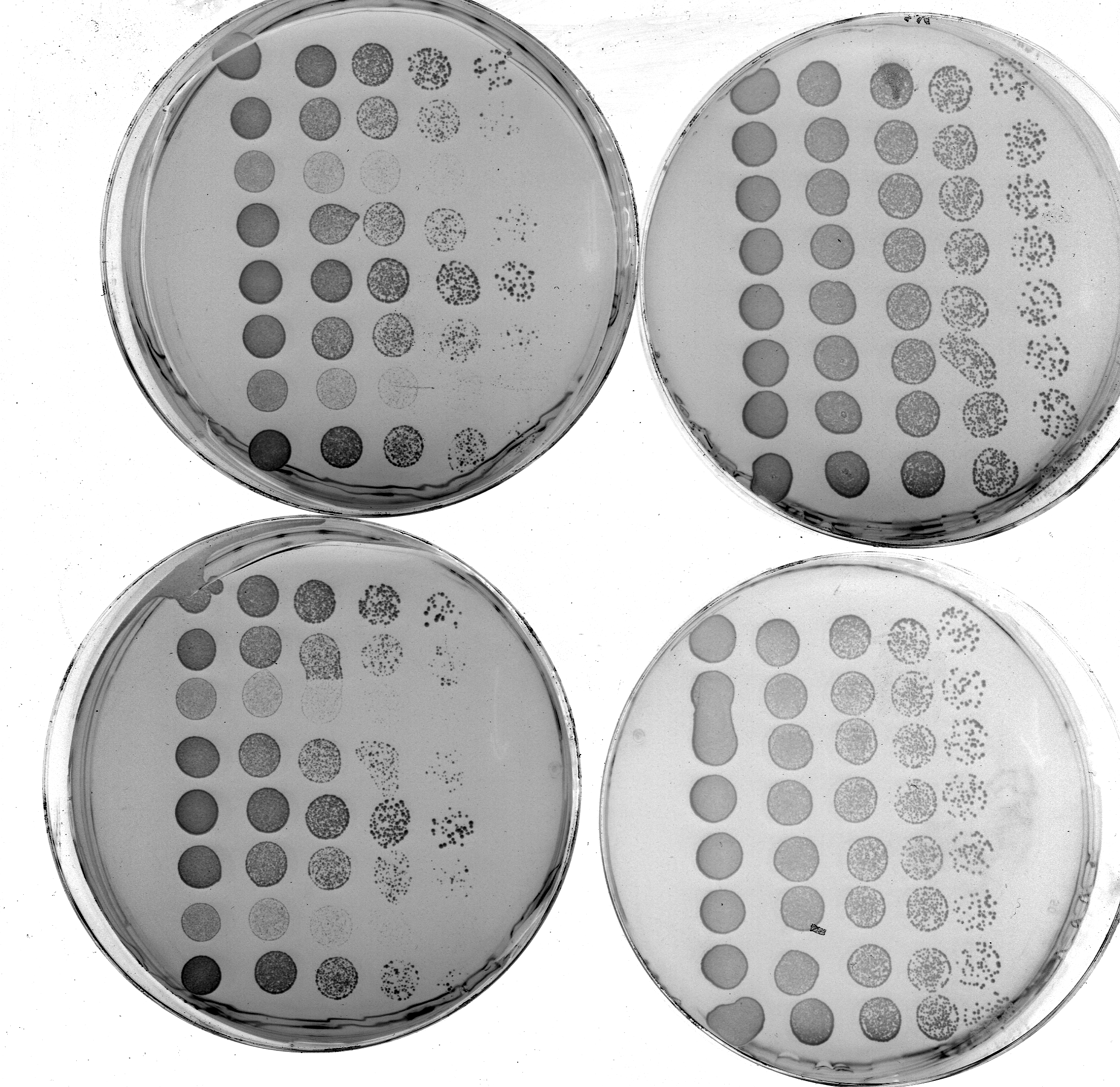

Supplement: Supplementary file 4 — Source data Fig. 3 [file 44318_2024_355_MOESM4_ESM.zip › Figure 3/3C/Spot growth assay (Gluc Gal).tif]

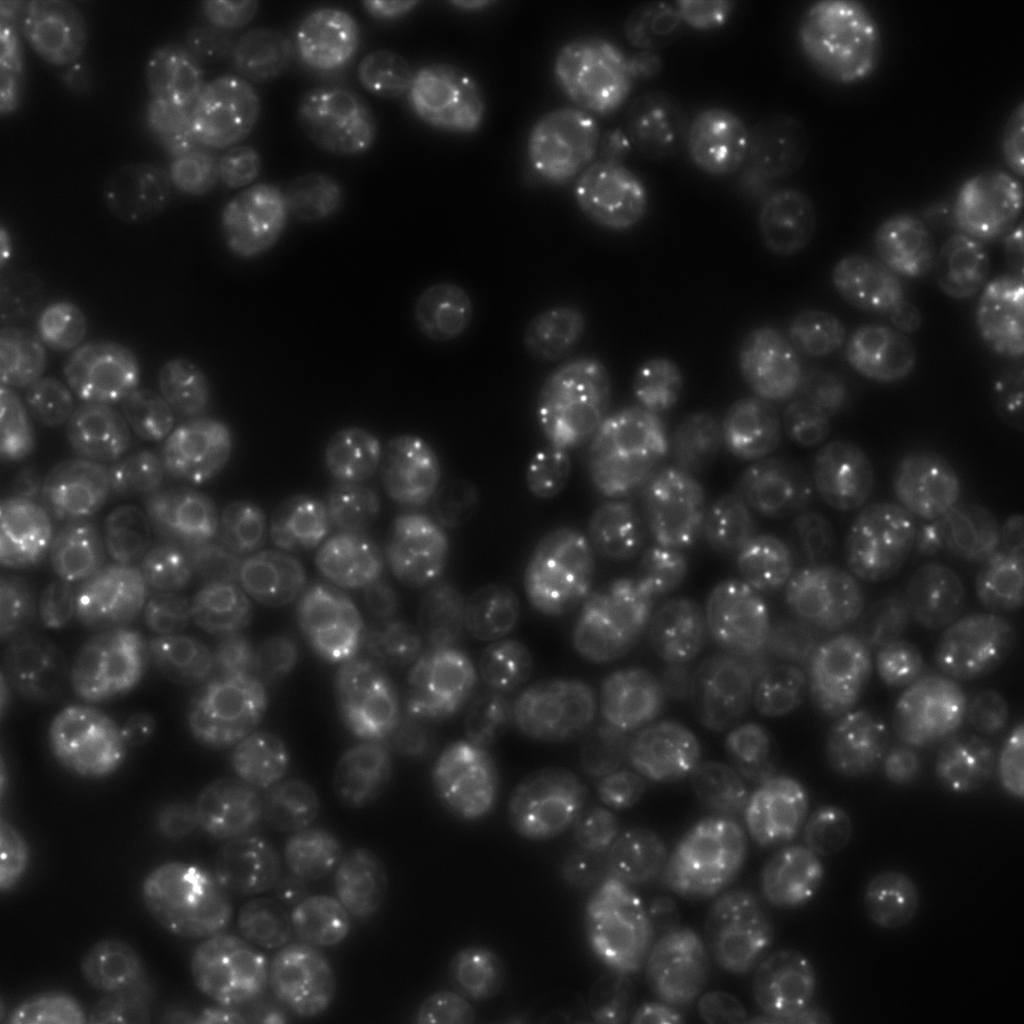

Supplement: Supplementary file 4 — Source data Fig. 3 [file 44318_2024_355_MOESM4_ESM.zip › Figure 3/3A/C1-Snap-1347(GAL-Lro1).tif]

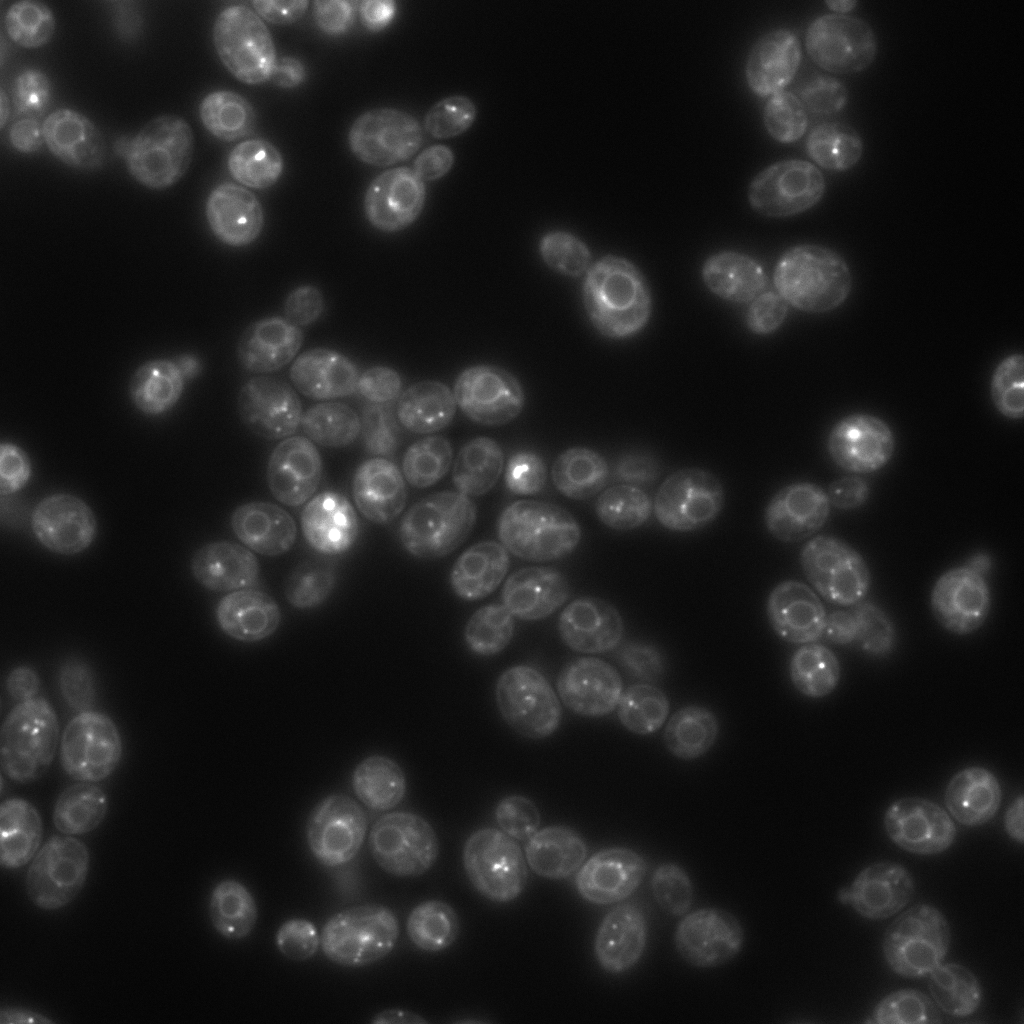

Supplement: Supplementary file 4 — Source data Fig. 3 [file 44318_2024_355_MOESM4_ESM.zip › Figure 3/3A/C1-Snap-1342(vector).tif]

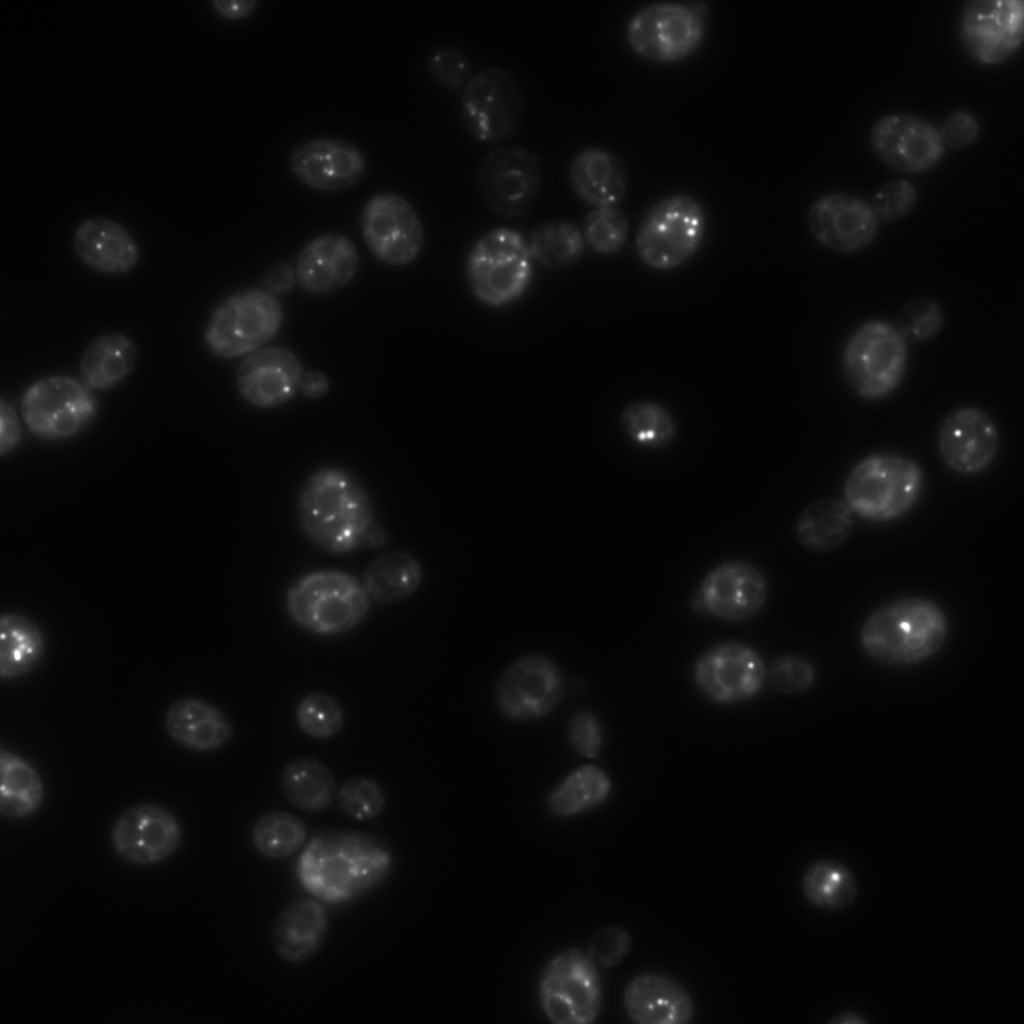

Supplement: Supplementary file 4 — Source data Fig. 3 [file 44318_2024_355_MOESM4_ESM.zip › Figure 3/3A/C1-Snap-1352(GAL-Lro1 S324A).tif]

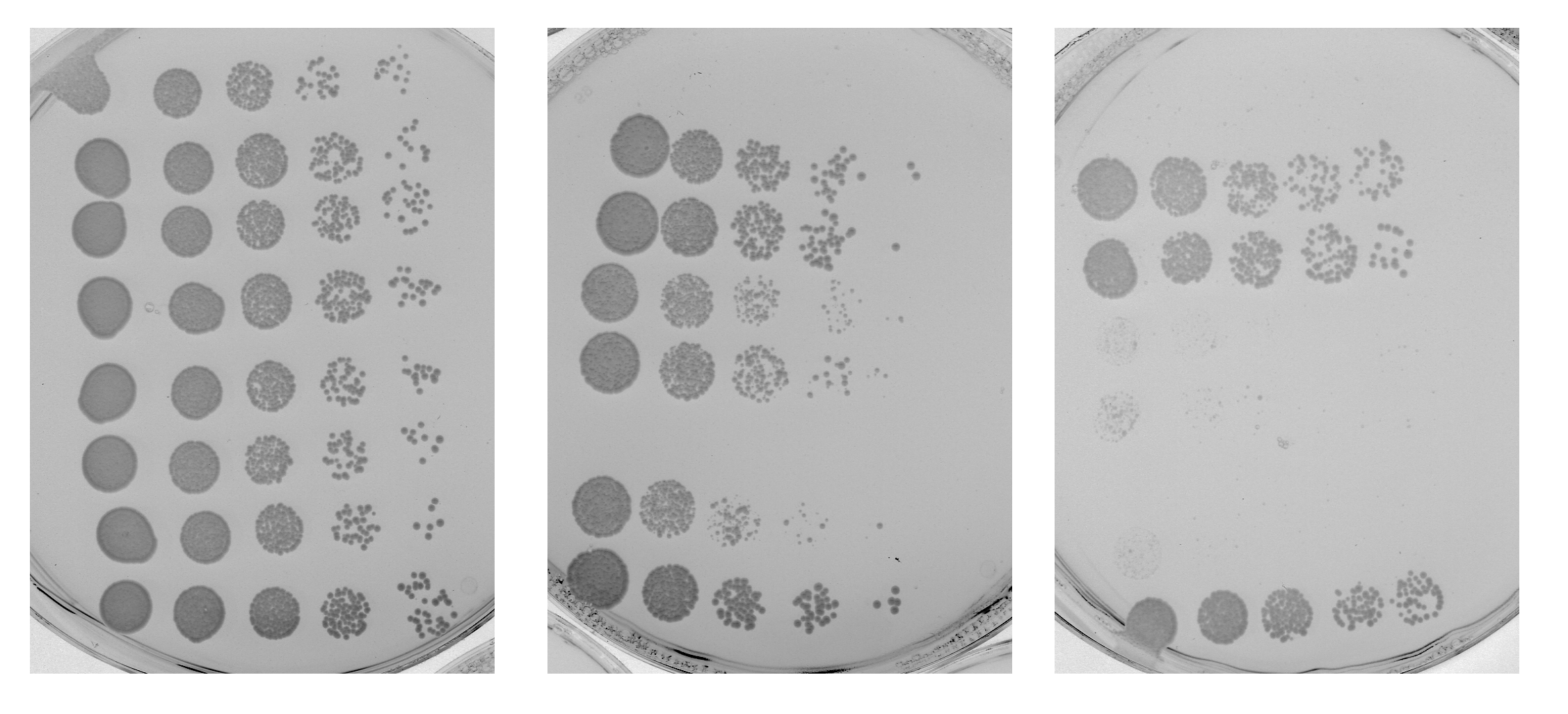

Supplement: Supplementary file 5 — Source data Fig. 4 [file 44318_2024_355_MOESM5_ESM.zip › Figure 4/4B/Spot growth assays ( plus minus oleate).tif]

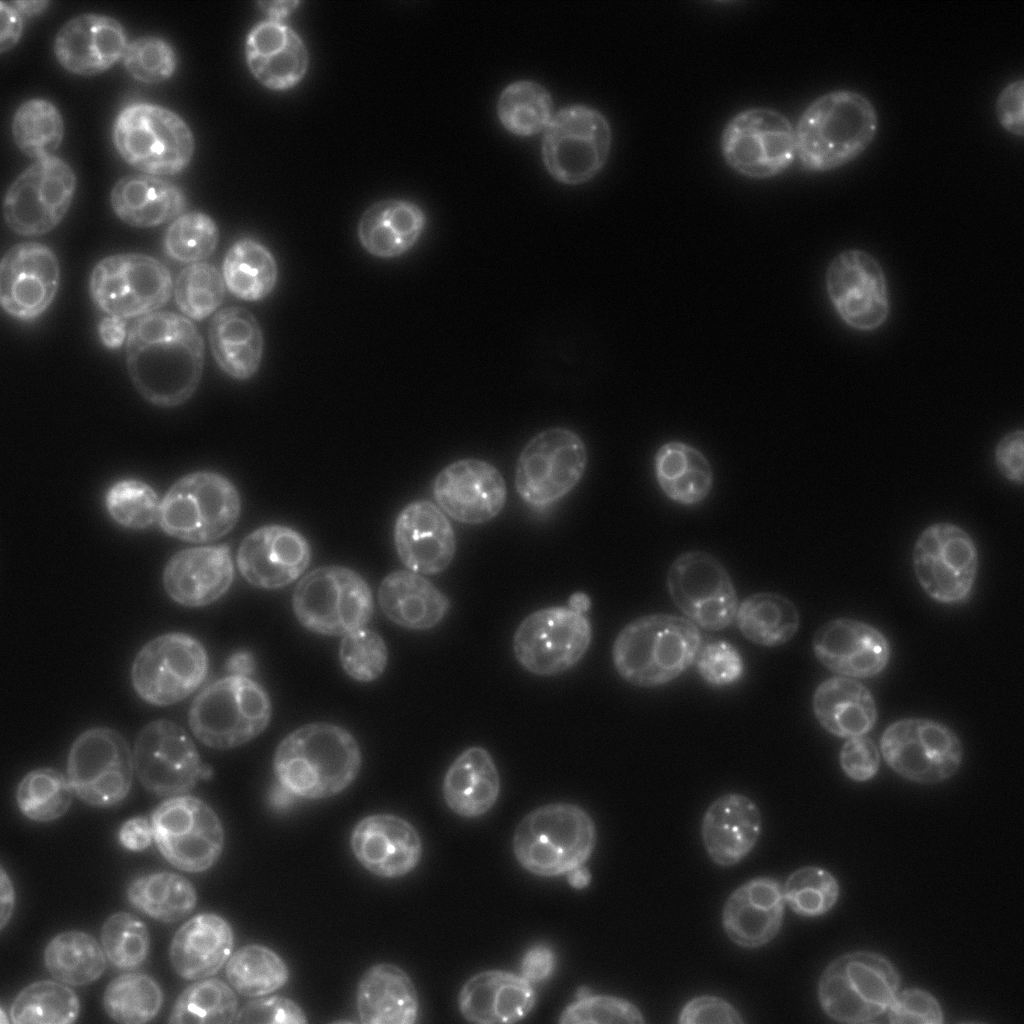

Supplement: Supplementary file 5 — Source data Fig. 4 [file 44318_2024_355_MOESM5_ESM.zip › Figure 4/4C/593 (GS string-mCh BODIPY Exp).tif]

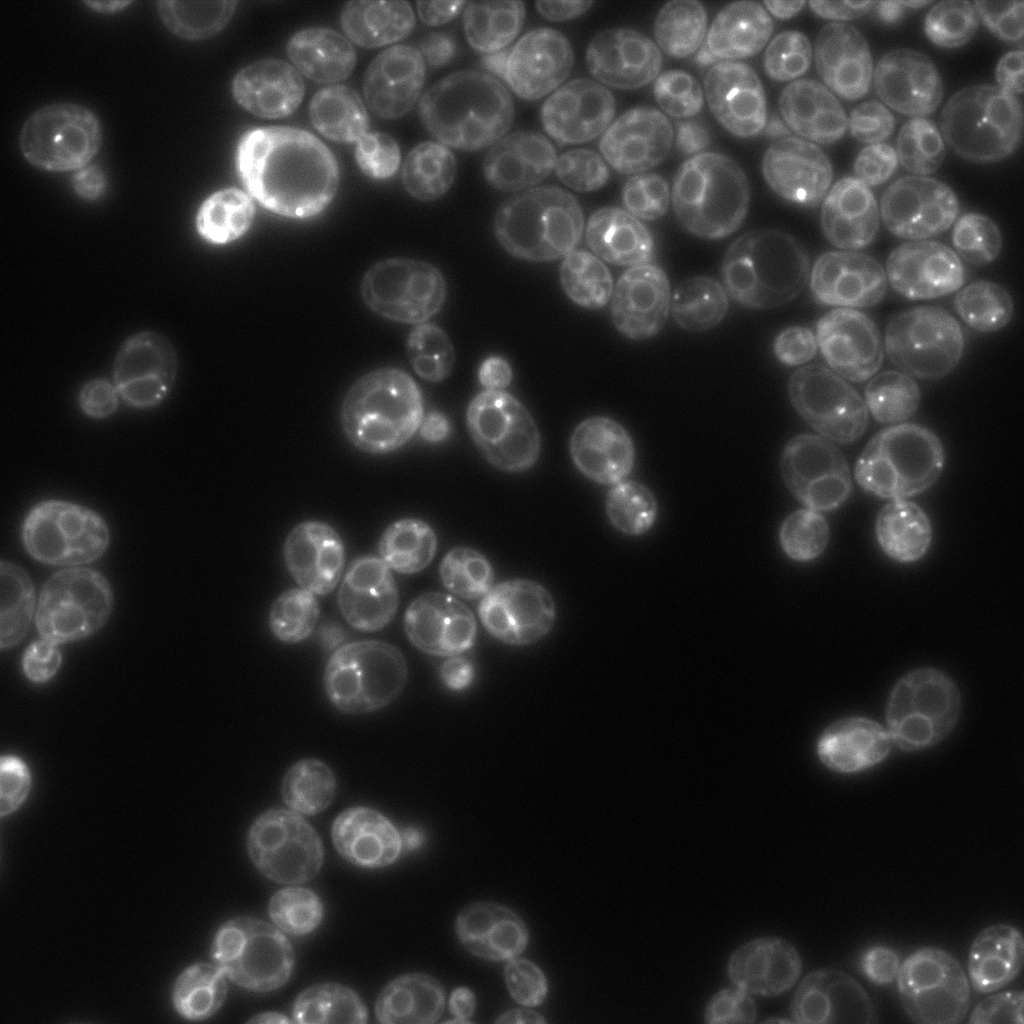

Supplement: Supplementary file 5 — Source data Fig. 4 [file 44318_2024_355_MOESM5_ESM.zip › Figure 4/4C/569 (Lro1-mCh BODIPY Exp).tif]

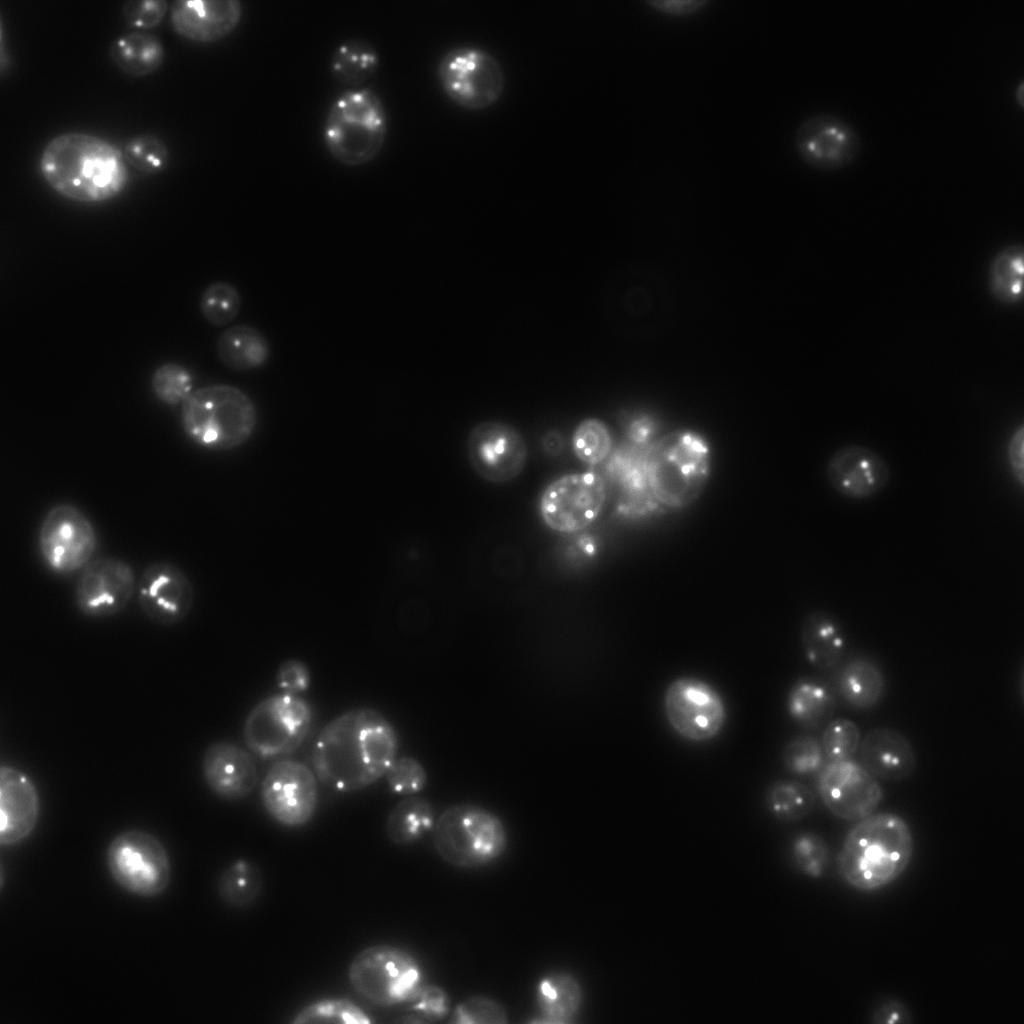

Supplement: Supplementary file 5 — Source data Fig. 4 [file 44318_2024_355_MOESM5_ESM.zip › Figure 4/4C/621 (GS string-mCh BODIPY Stat).tif]

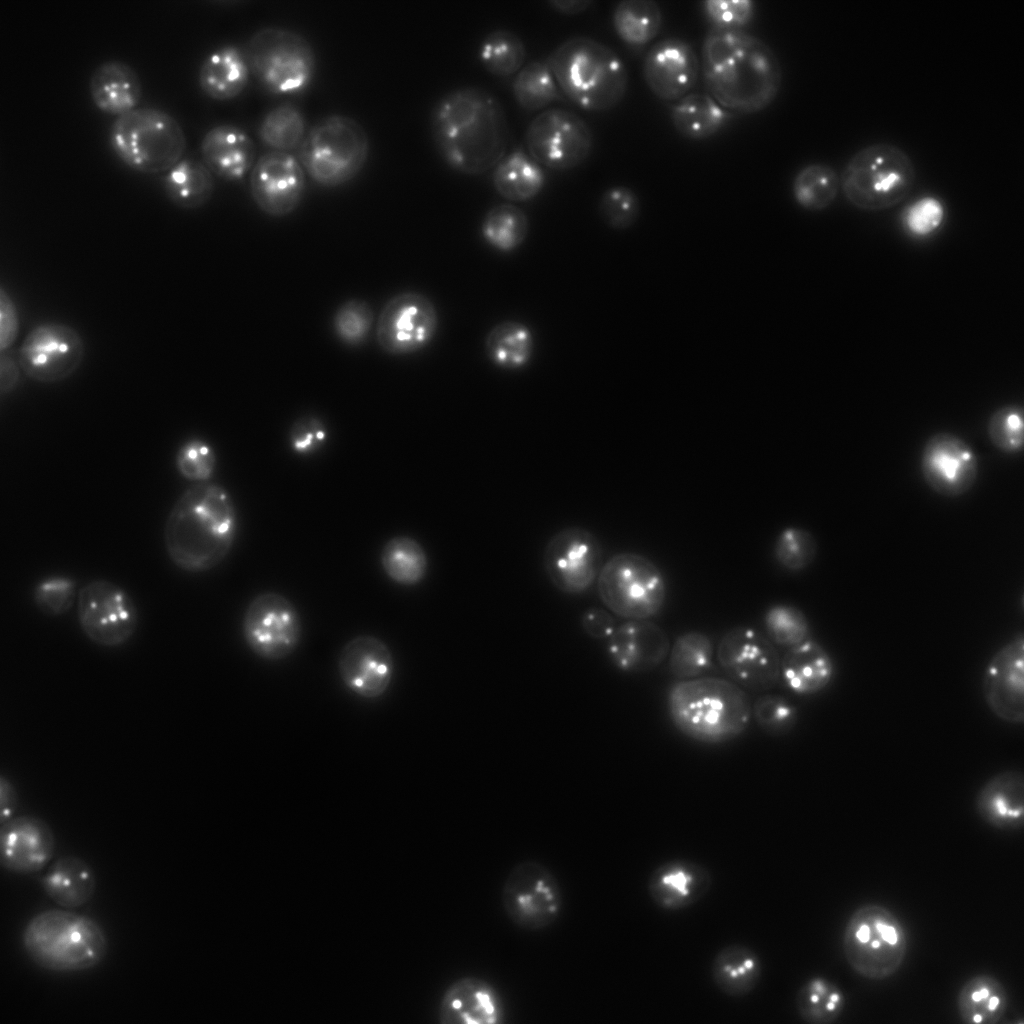

Supplement: Supplementary file 5 — Source data Fig. 4 [file 44318_2024_355_MOESM5_ESM.zip › Figure 4/4C/605 (Lro1-mCh BODIPY Stat).tif]

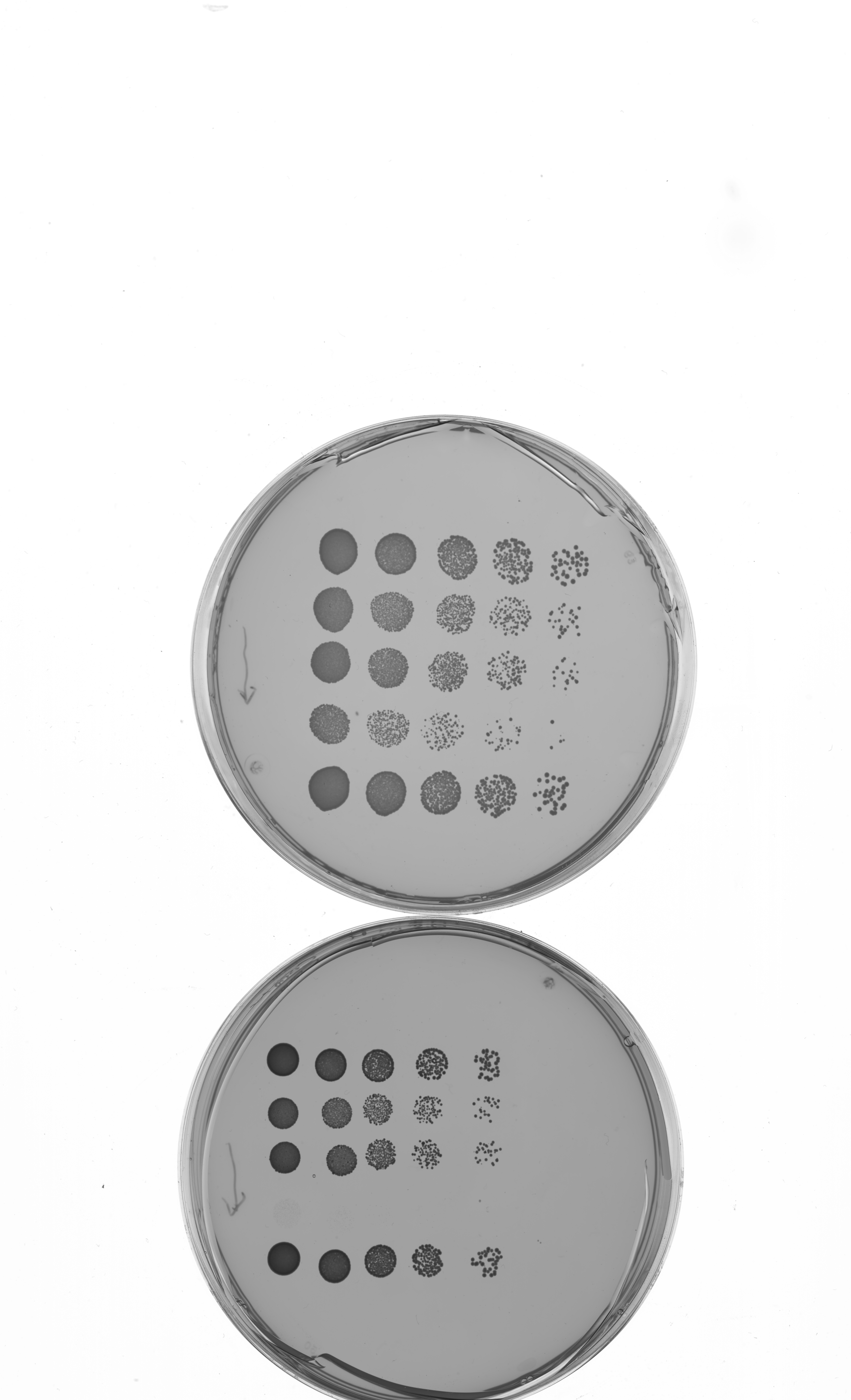

Supplement: Supplementary file 6 — Source data Fig. 5 [file 44318_2024_355_MOESM6_ESM.zip › Figure 5/5D/Spot growth assay (plus minus copper).tif]

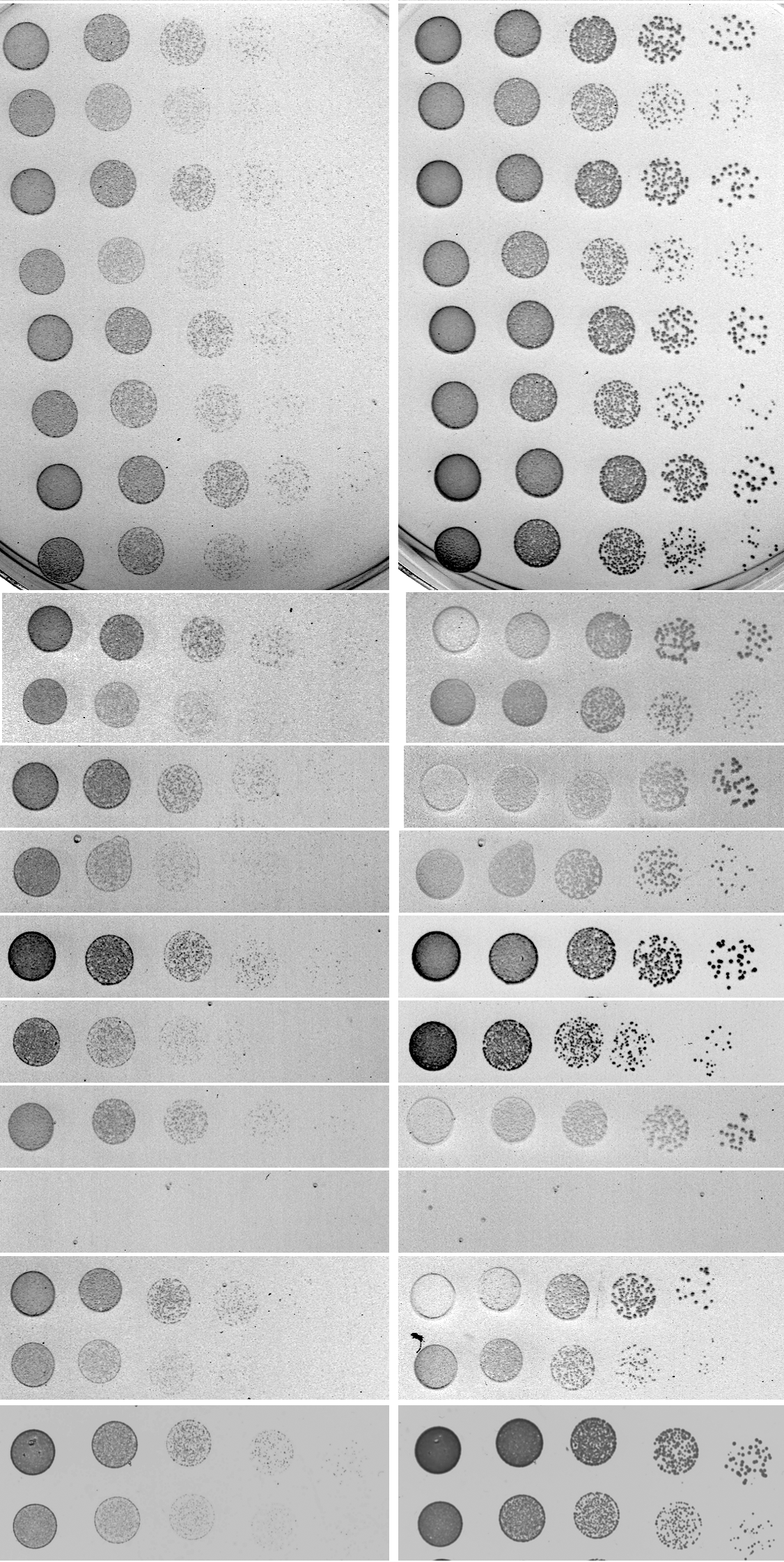

Supplement: Supplementary file 6 — Source data Fig. 5 [file 44318_2024_355_MOESM6_ESM.zip › Figure 5/5B/Spot growth assay (puls minus copper).tif]

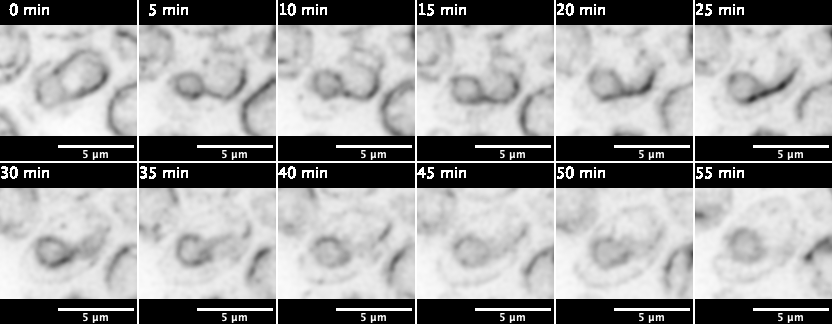

Supplement: Supplementary file 7 — Source data Fig. 6 [file 44318_2024_355_MOESM7_ESM.zip › Figure 6/6F/6F montage time lapse.tif]

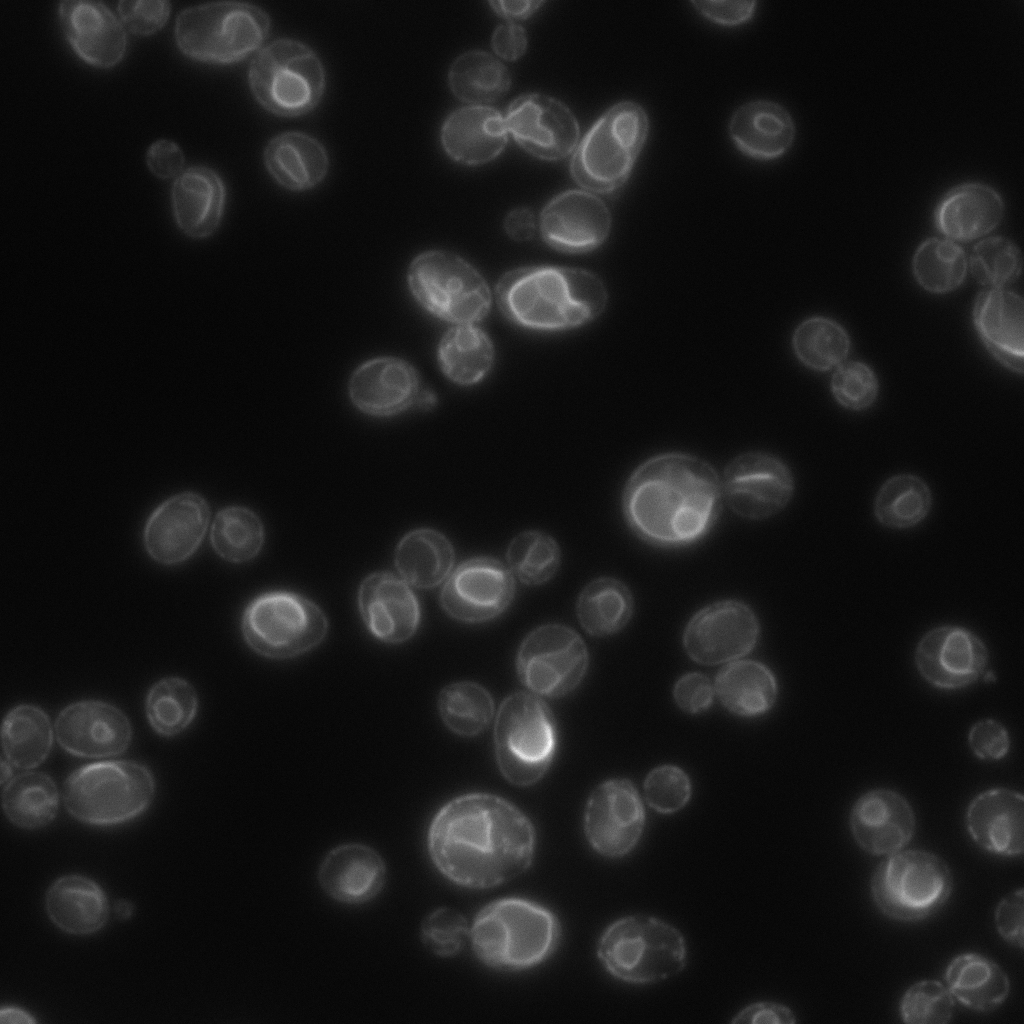

Supplement: Supplementary file 7 — Source data Fig. 6 [file 44318_2024_355_MOESM7_ESM.zip › Figure 6/6H/28-1 Sec63-mNG.tif]

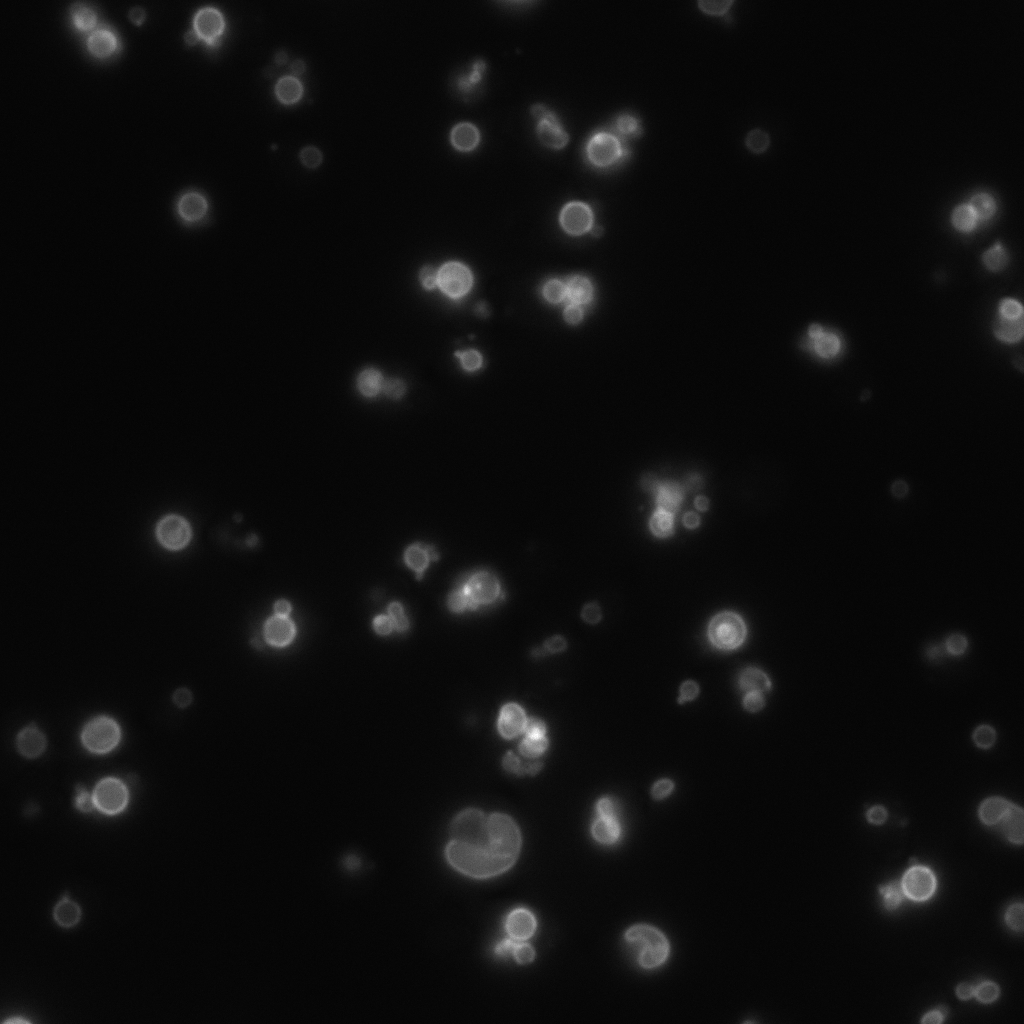

Supplement: Supplementary file 7 — Source data Fig. 6 [file 44318_2024_355_MOESM7_ESM.zip › Figure 6/6H/28-1 Vph1-mCh.tif]

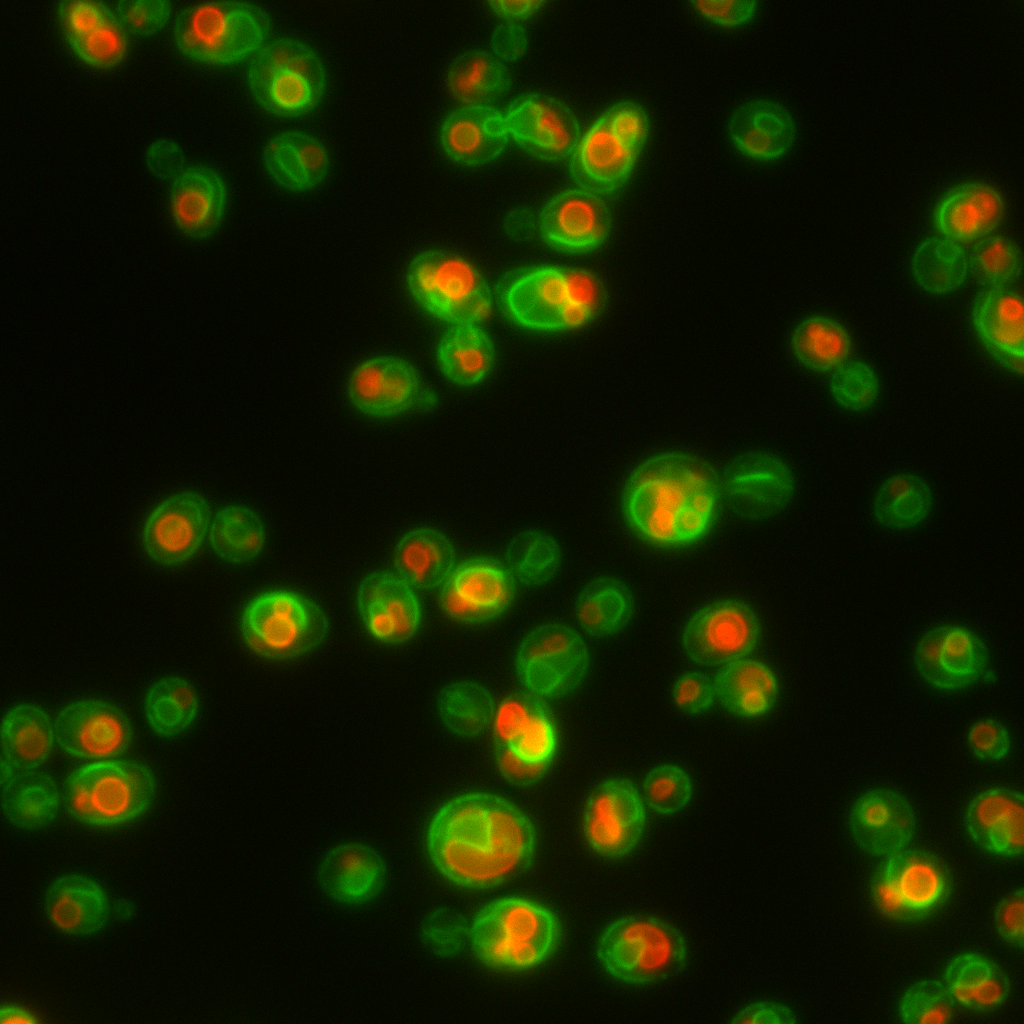

Supplement: Supplementary file 7 — Source data Fig. 6 [file 44318_2024_355_MOESM7_ESM.zip › Figure 6/6H/28-1 Merge.tif]

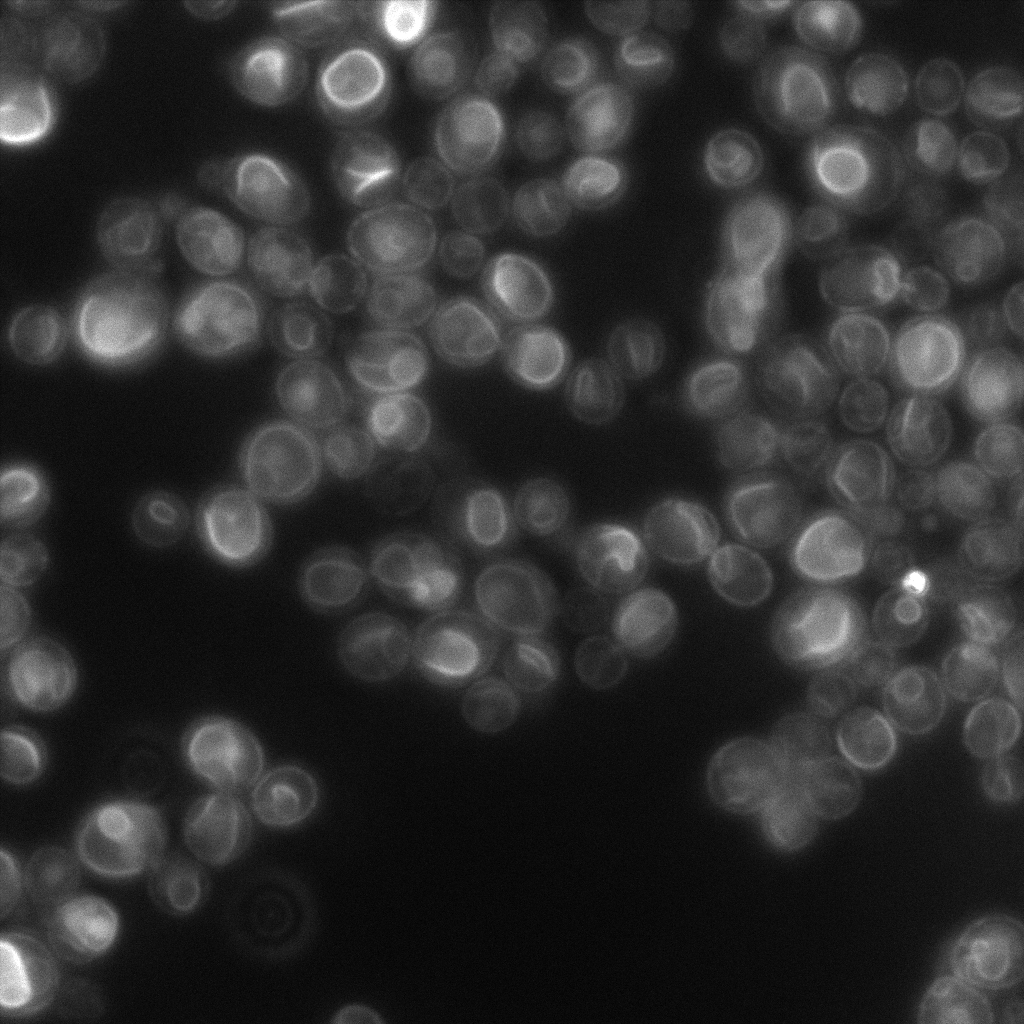

Supplement: Supplementary file 7 — Source data Fig. 6 [file 44318_2024_355_MOESM7_ESM.zip › Figure 6/6E/C2-Snap-854 Vector (Sec63-mCh).tif]

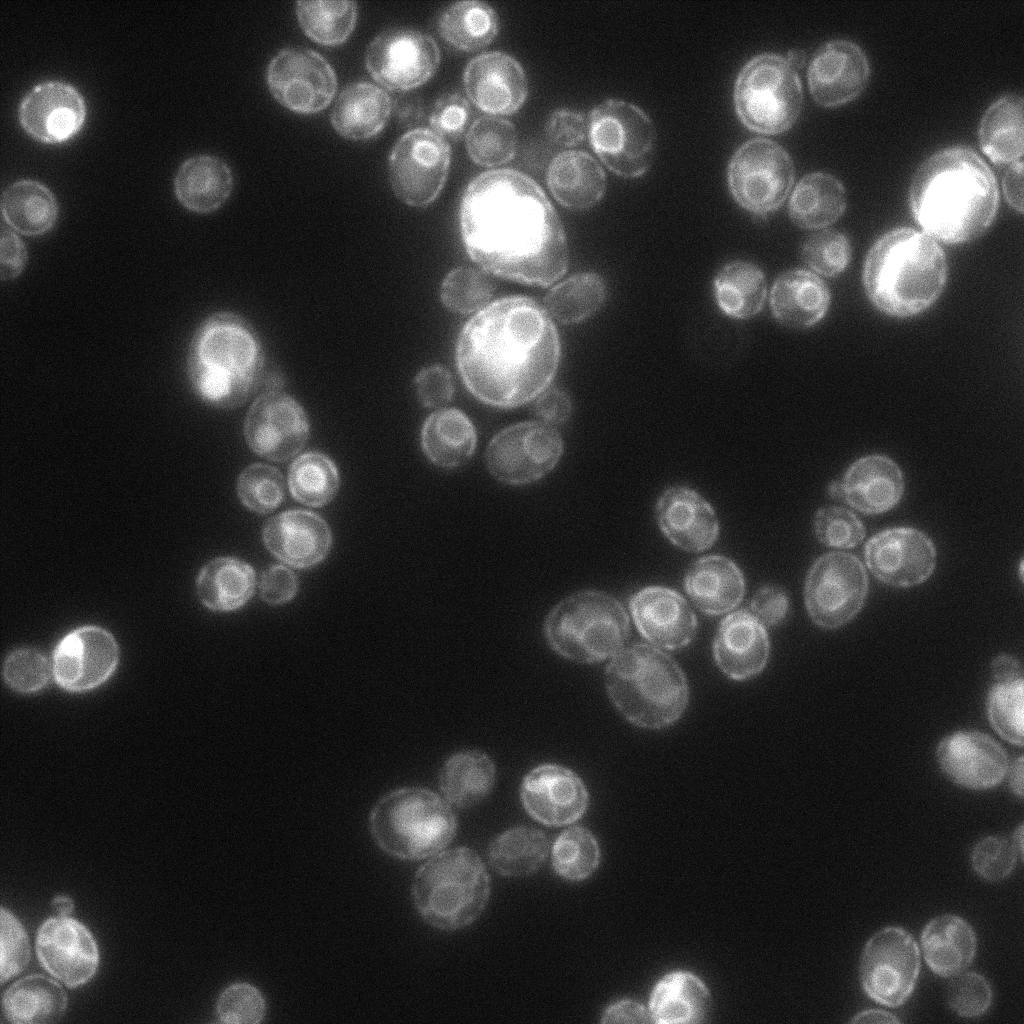

Supplement: Supplementary file 7 — Source data Fig. 6 [file 44318_2024_355_MOESM7_ESM.zip › Figure 6/6E/C2-Snap-861 Cup1-Lro1* (Sec63-mCh).tif]

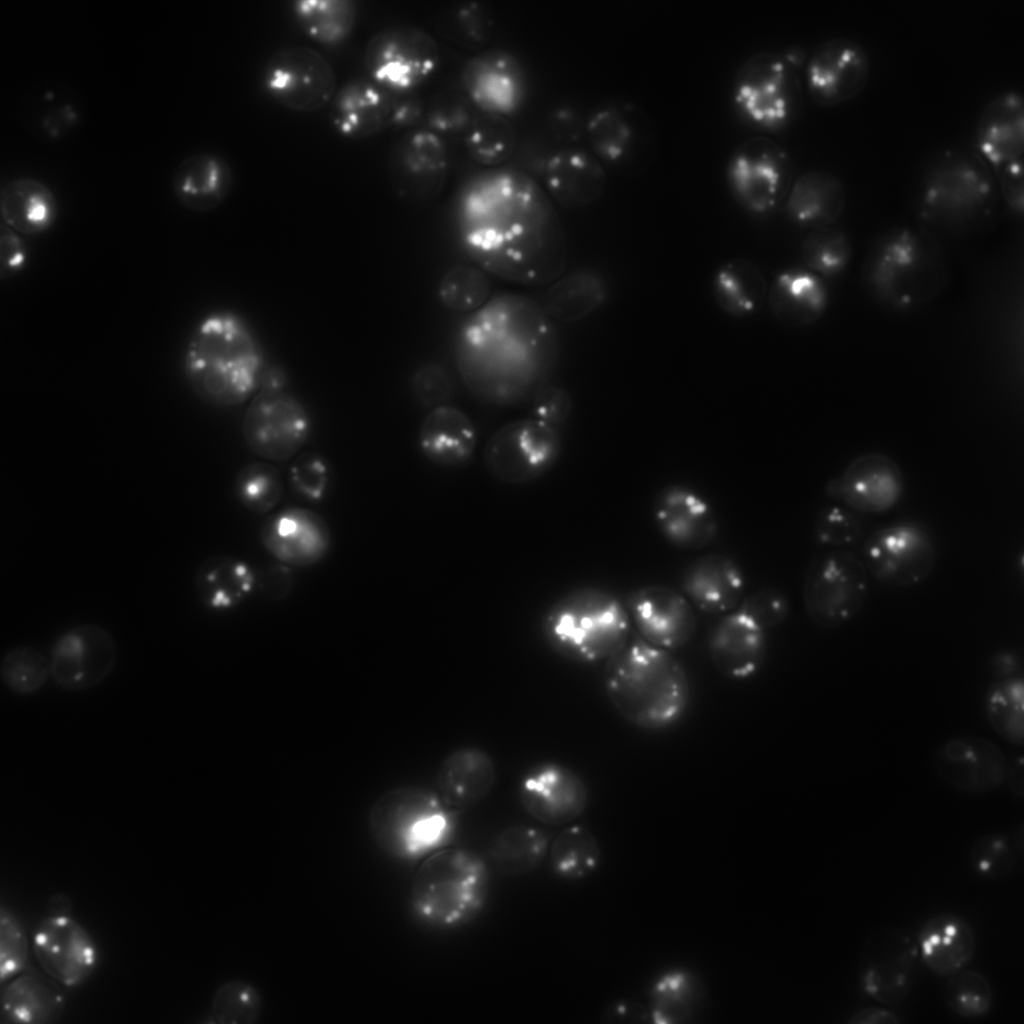

Supplement: Supplementary file 7 — Source data Fig. 6 [file 44318_2024_355_MOESM7_ESM.zip › Figure 6/6E/C1-Snap-861 Cup1-Lro1* (BODIPY).tif]

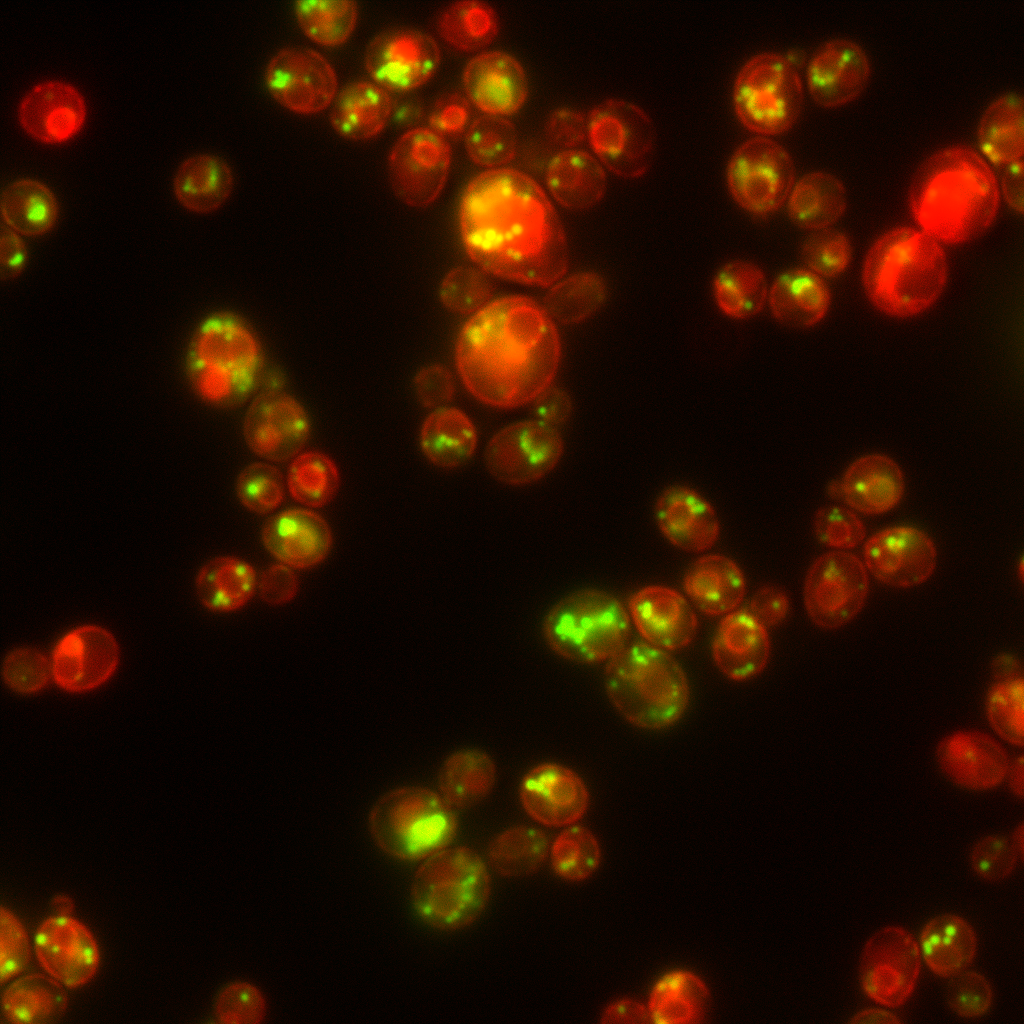

Supplement: Supplementary file 7 — Source data Fig. 6 [file 44318_2024_355_MOESM7_ESM.zip › Figure 6/6E/Merge-Cup1-Lro1*.tif]

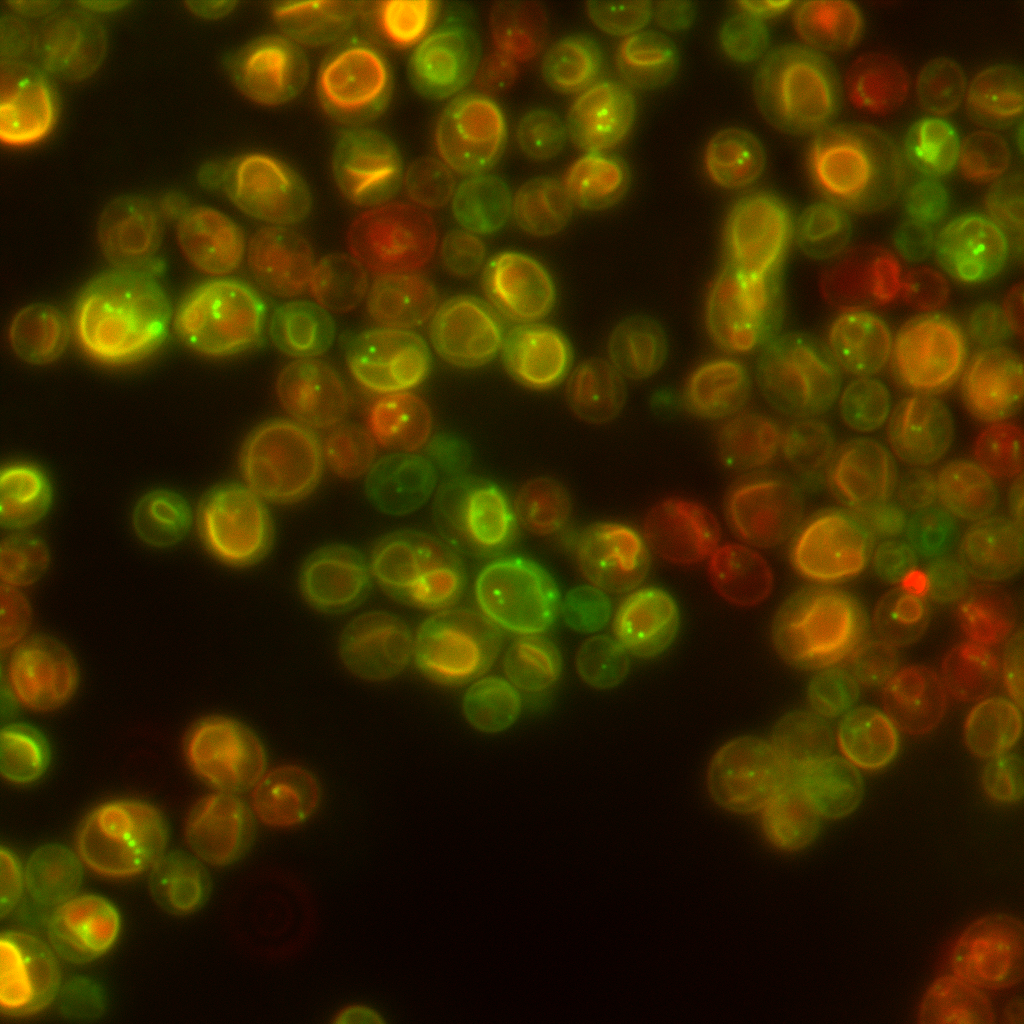

Supplement: Supplementary file 7 — Source data Fig. 6 [file 44318_2024_355_MOESM7_ESM.zip › Figure 6/6E/Merge -Vector.tif]

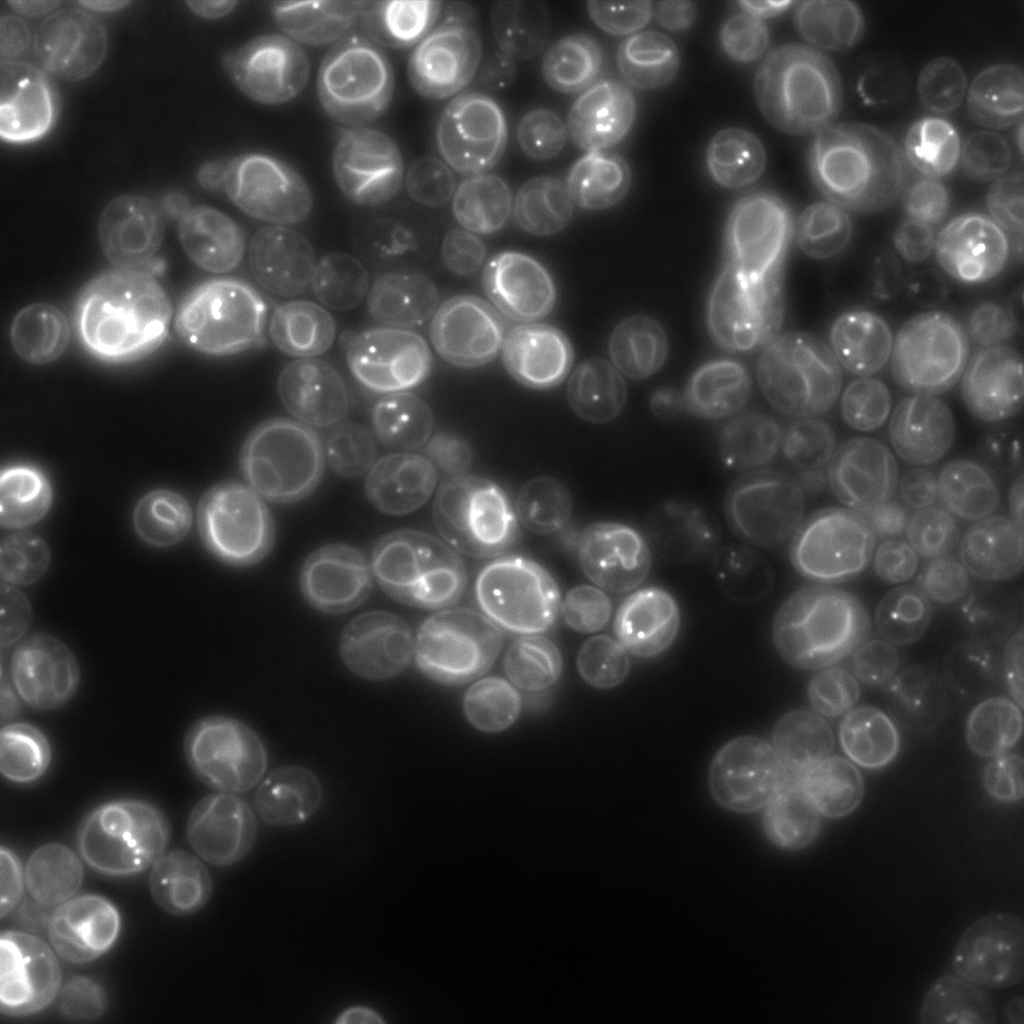

Supplement: Supplementary file 7 — Source data Fig. 6 [file 44318_2024_355_MOESM7_ESM.zip › Figure 6/6E/C1-Snap-854 Vector (BODIPY).tif]

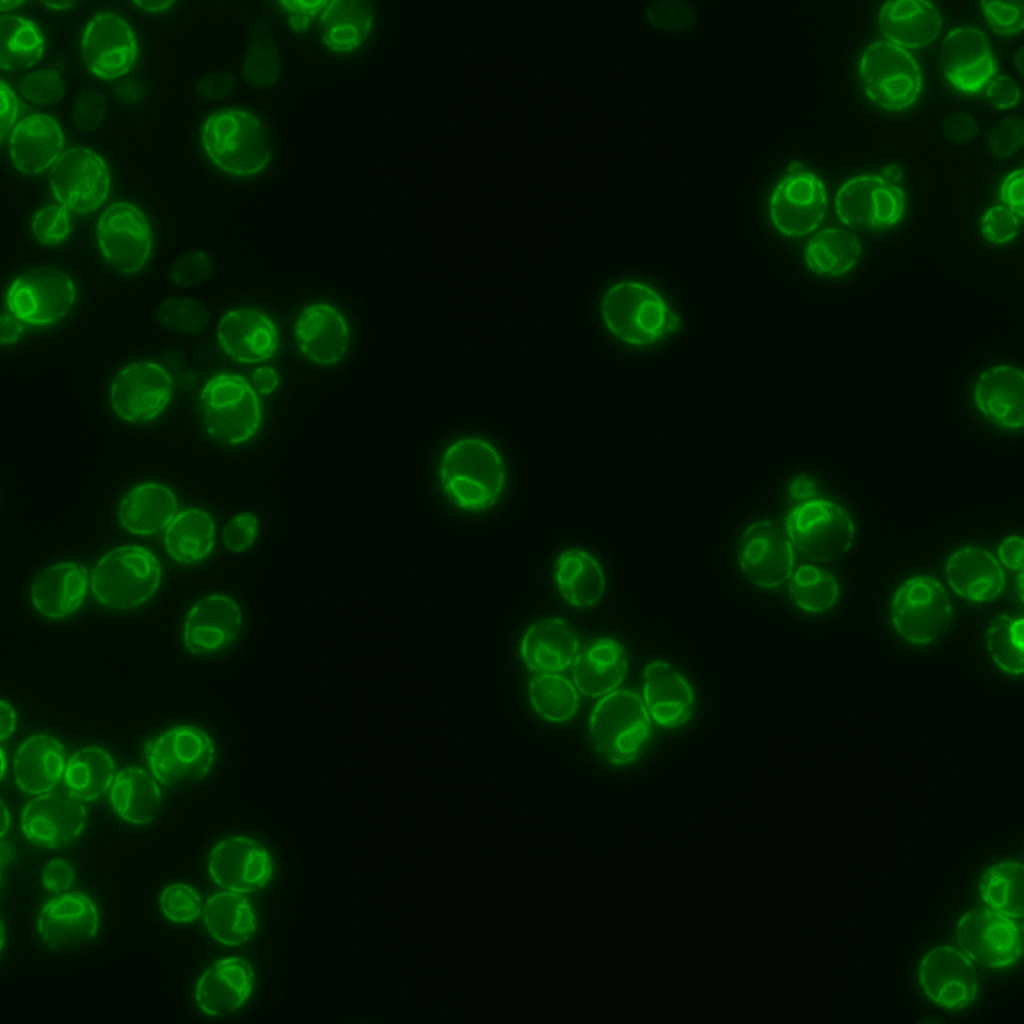

Supplement: Supplementary file 7 — Source data Fig. 6 [file 44318_2024_355_MOESM7_ESM.zip › Figure 6/6C/38 no dgk1 z15 -1 (Sec63-mNG) copy.tif]

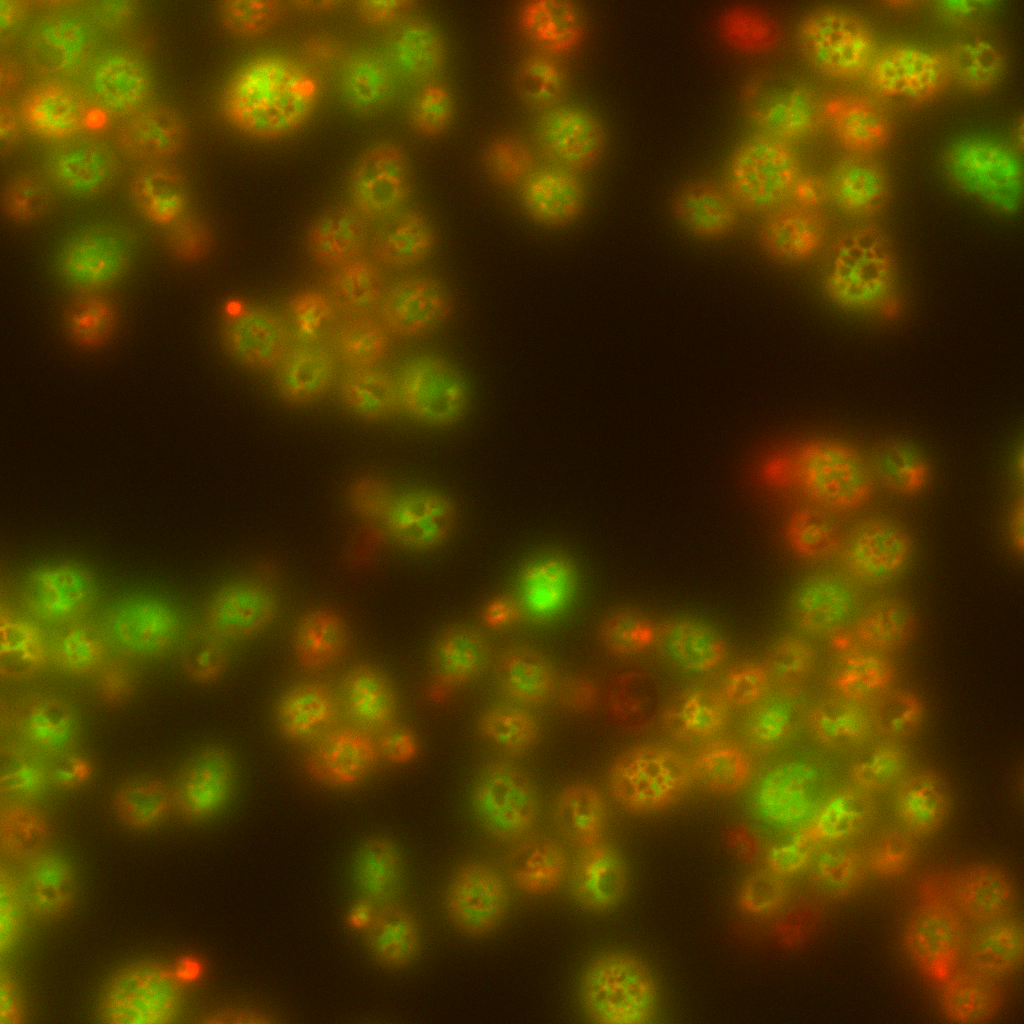

Supplement: Supplementary file 7 — Source data Fig. 6 [file 44318_2024_355_MOESM7_ESM.zip › Figure 6/6C/28 Cup1-Lro1* z8-1 MERGE copy.tif]

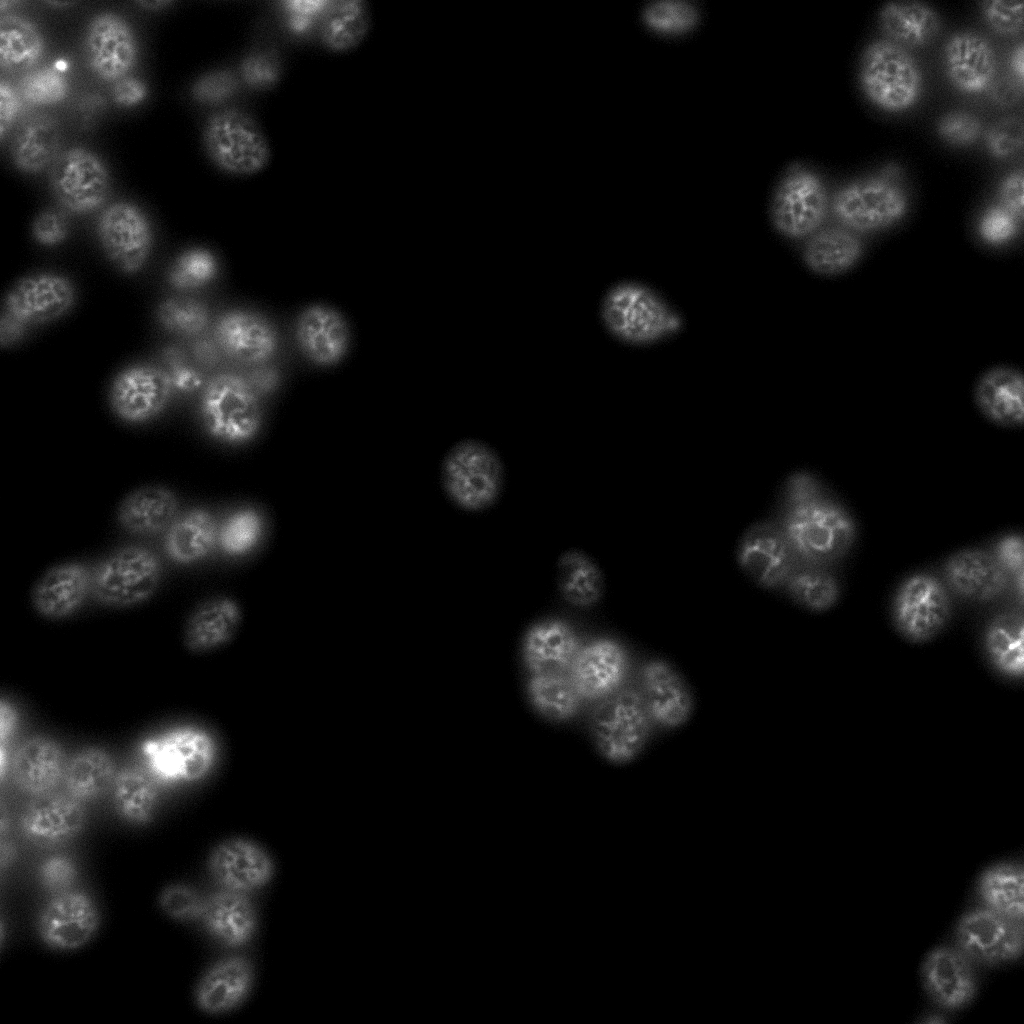

Supplement: Supplementary file 7 — Source data Fig. 6 [file 44318_2024_355_MOESM7_ESM.zip › Figure 6/6C/38 no dgk1-1 z7.tif (Rtn1-mCh)-1 copy.tif]

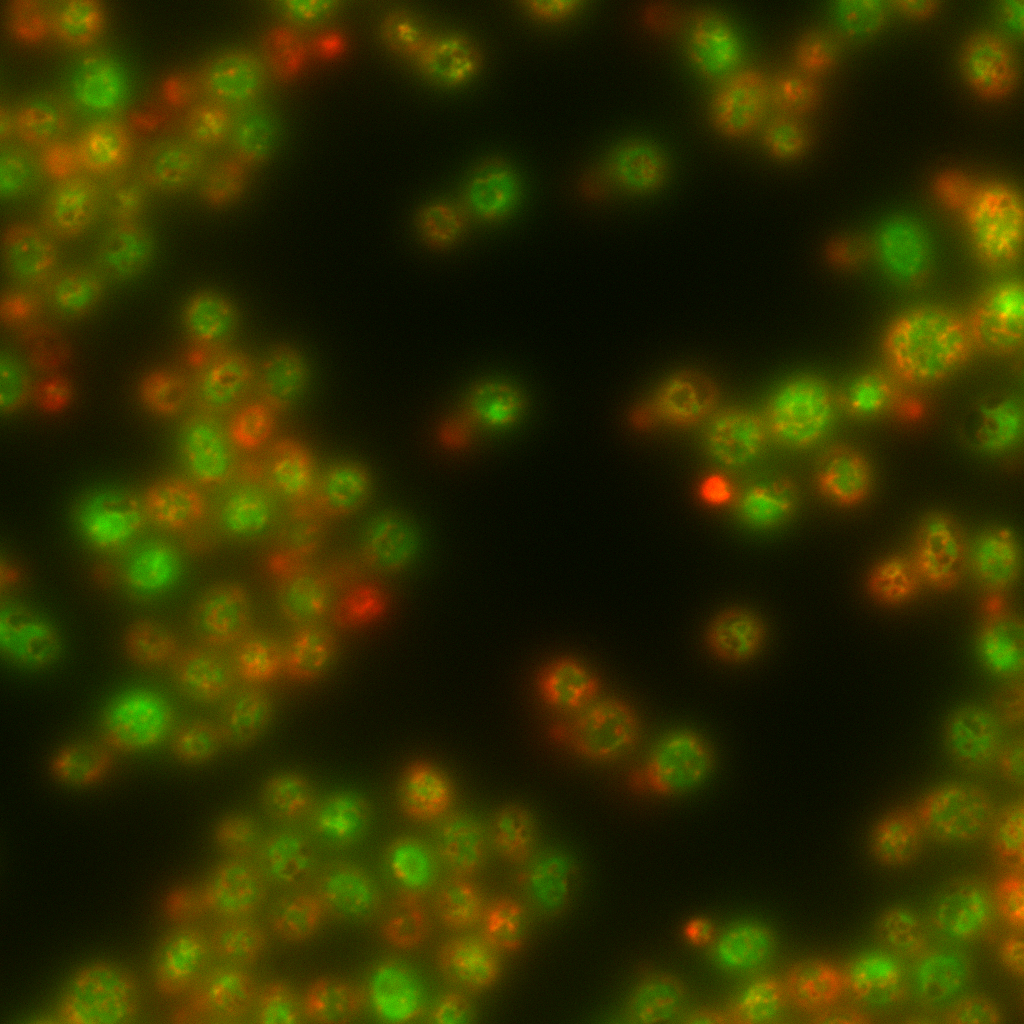

Supplement: Supplementary file 7 — Source data Fig. 6 [file 44318_2024_355_MOESM7_ESM.zip › Figure 6/6C/22 Cup1-Lro1*[S324A] z7 MERGE copy.tif]

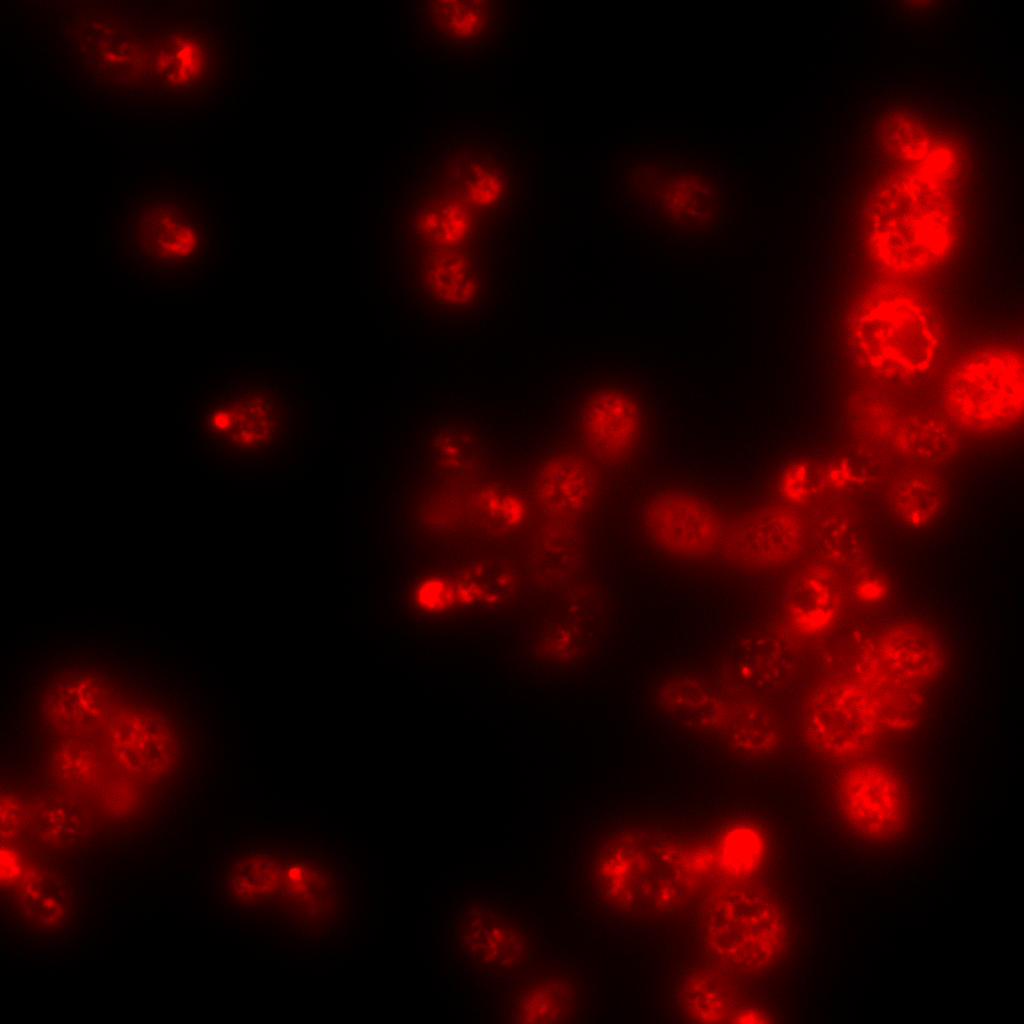

Supplement: Supplementary file 7 — Source data Fig. 6 [file 44318_2024_355_MOESM7_ESM.zip › Figure 6/6C/35 pura z7-1 (Rtn1-mCh) copy.tif]

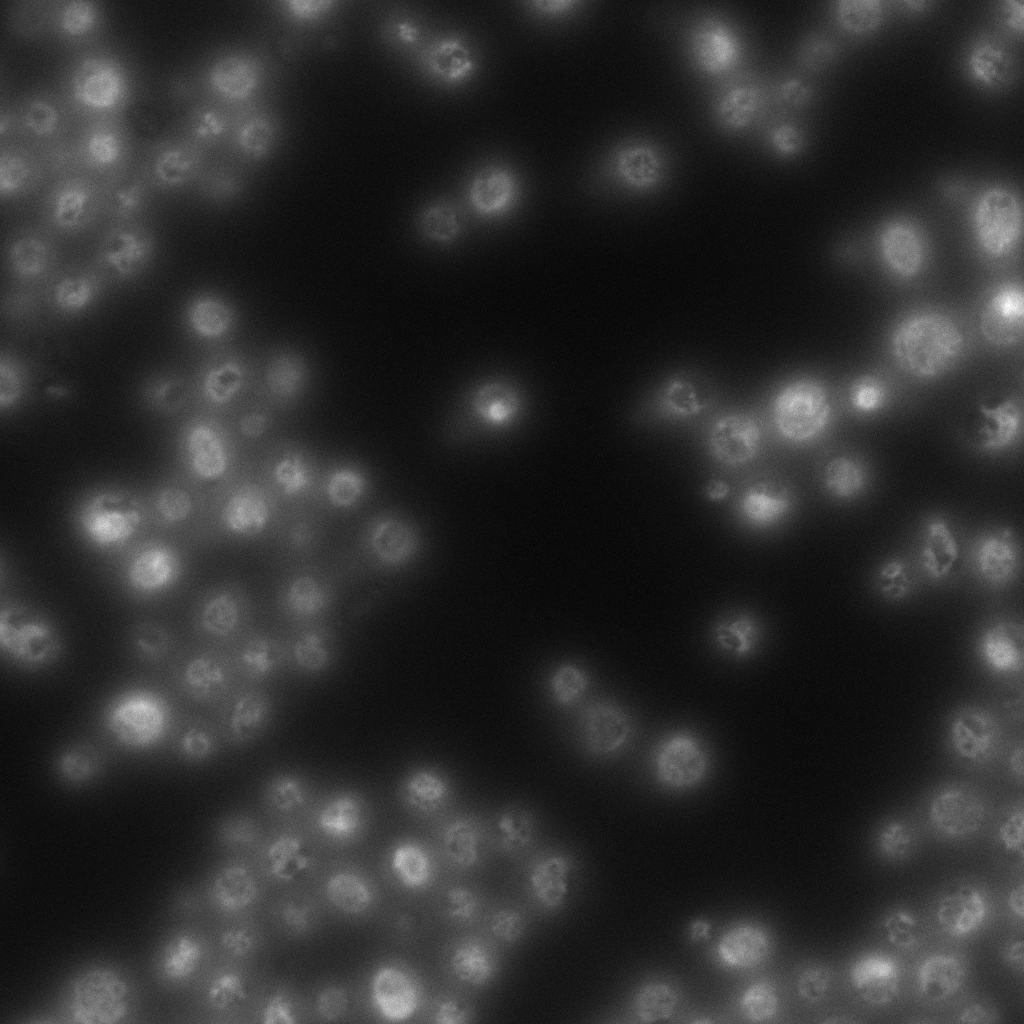

Supplement: Supplementary file 7 — Source data Fig. 6 [file 44318_2024_355_MOESM7_ESM.zip › Figure 6/6C/22 Cup1-Lro1*[S324A] z7 (Sec63-mNG) copy.tif]

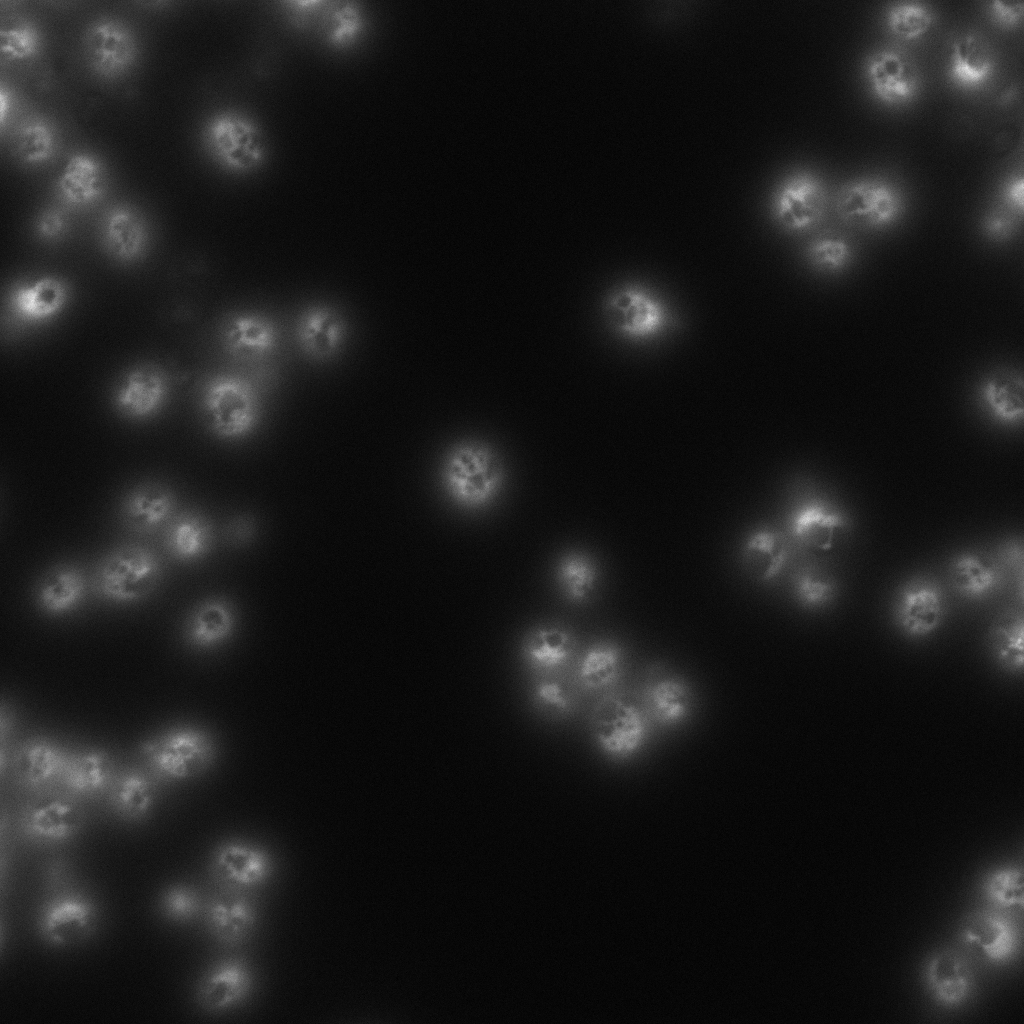

Supplement: Supplementary file 7 — Source data Fig. 6 [file 44318_2024_355_MOESM7_ESM.zip › Figure 6/6C/38 no dgk1-1 z7.tif (Sec63-mNG)-1 copy.tif]

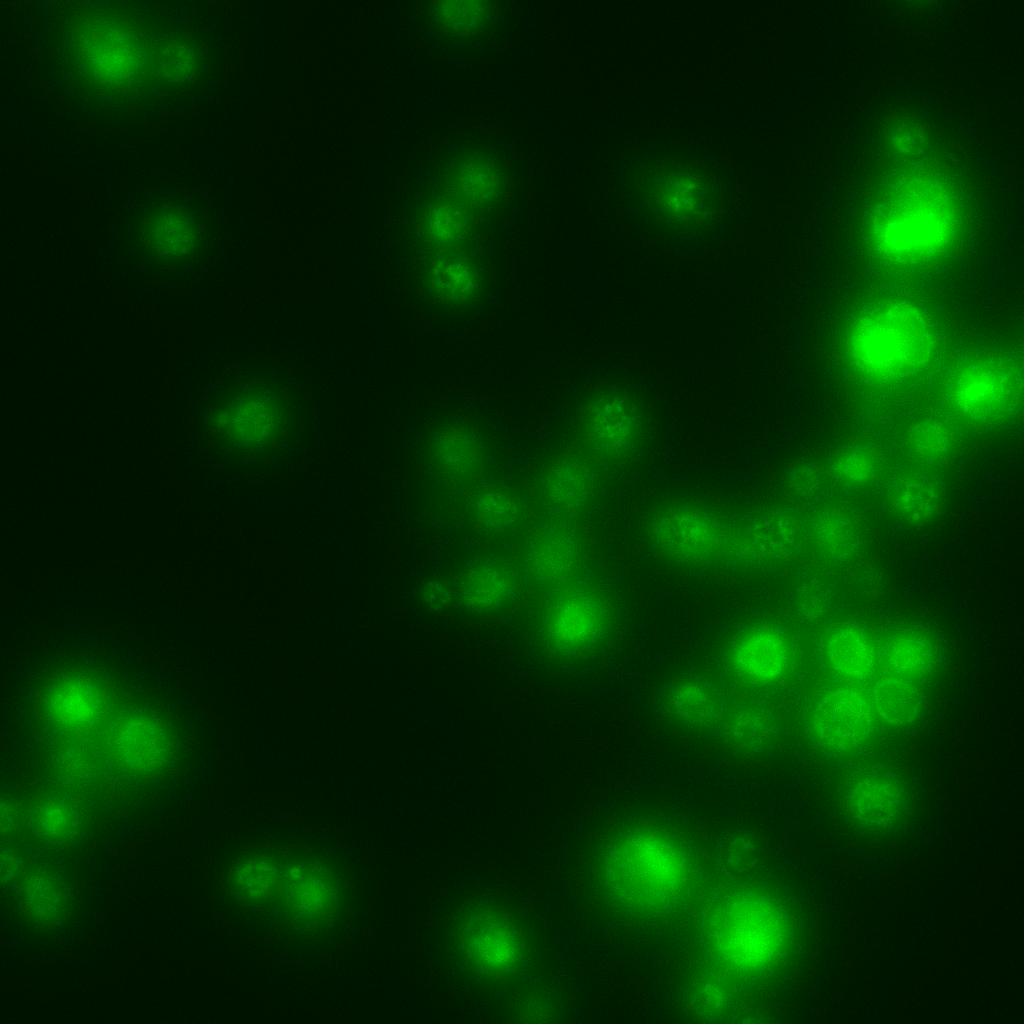

Supplement: Supplementary file 7 — Source data Fig. 6 [file 44318_2024_355_MOESM7_ESM.zip › Figure 6/6C/35 pura z7-1 (Sec63-mNG) copy.tif]

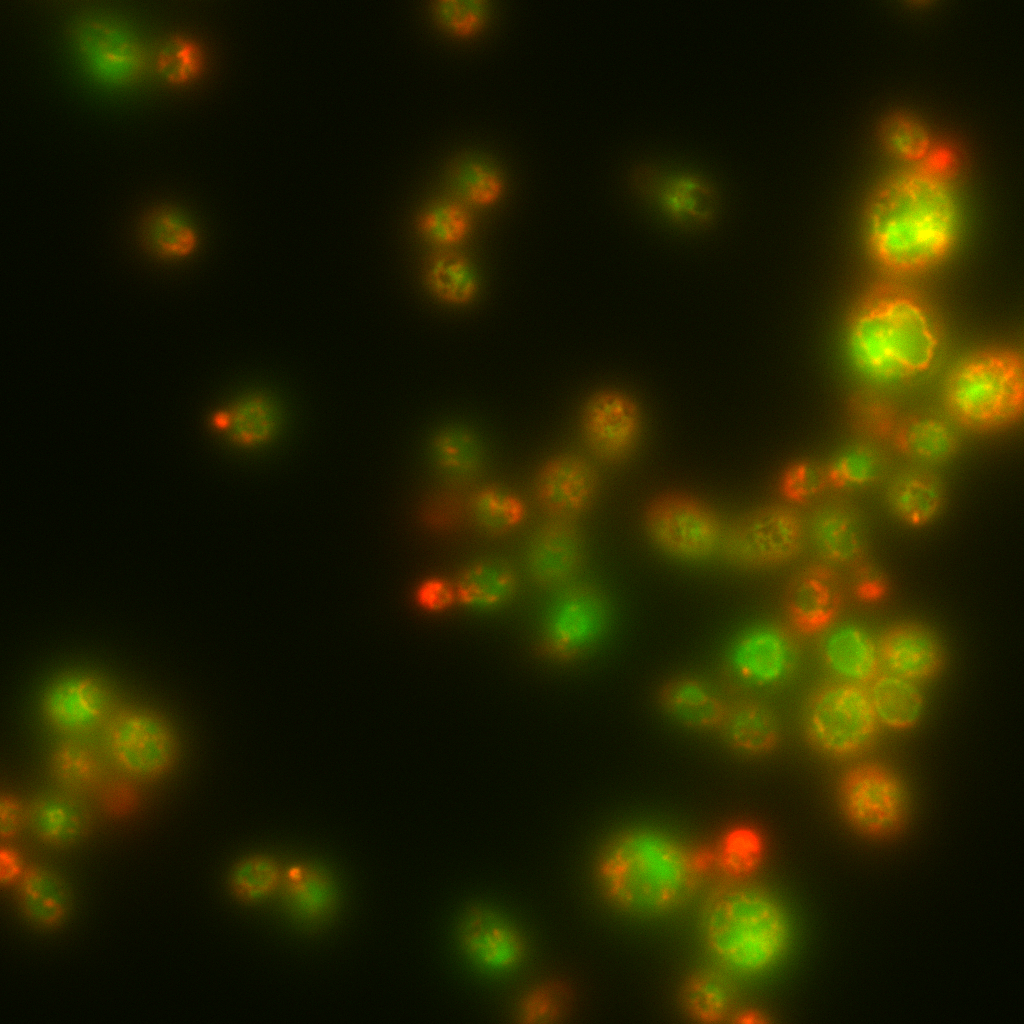

Supplement: Supplementary file 7 — Source data Fig. 6 [file 44318_2024_355_MOESM7_ESM.zip › Figure 6/6C/35 pura z7 -1 MERGE copy.tif]

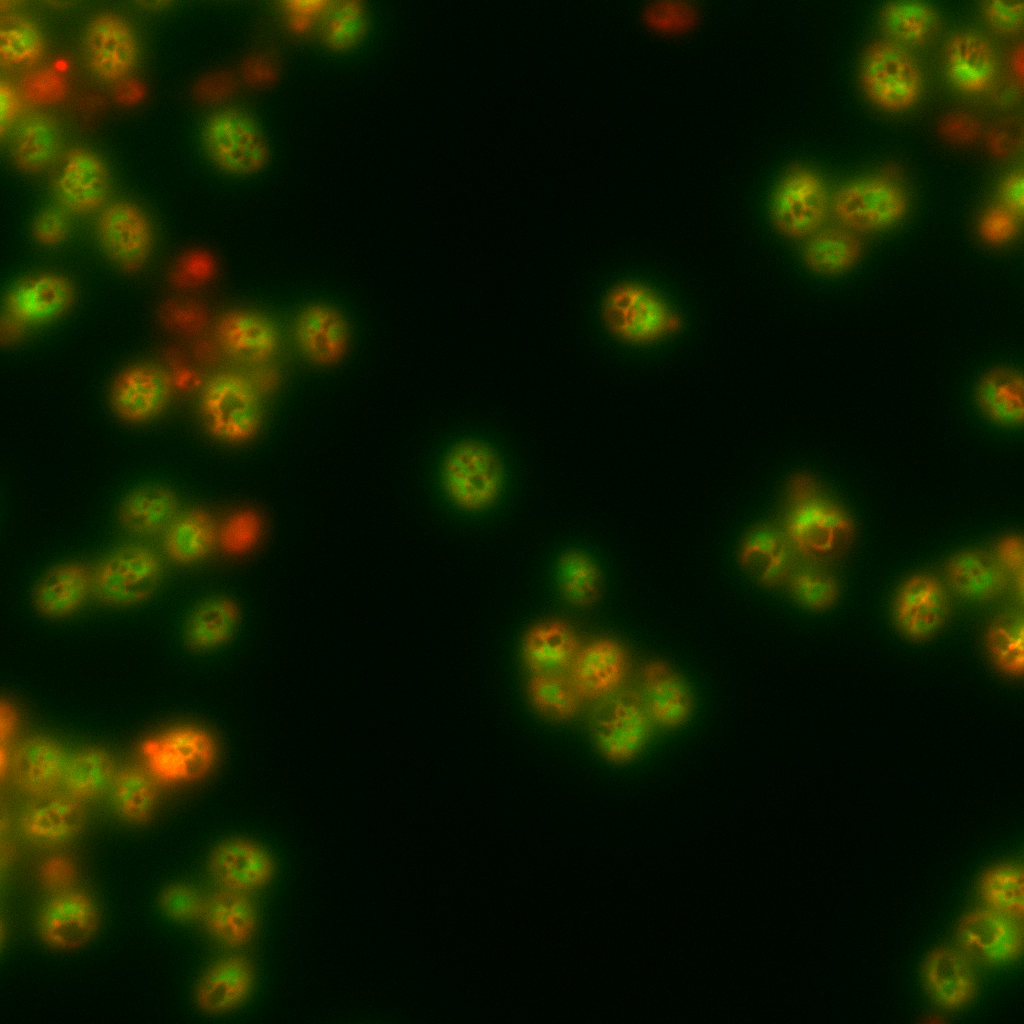

Supplement: Supplementary file 7 — Source data Fig. 6 [file 44318_2024_355_MOESM7_ESM.zip › Figure 6/6C/38 no dgk1-1 MERGE z7 copy.tif]

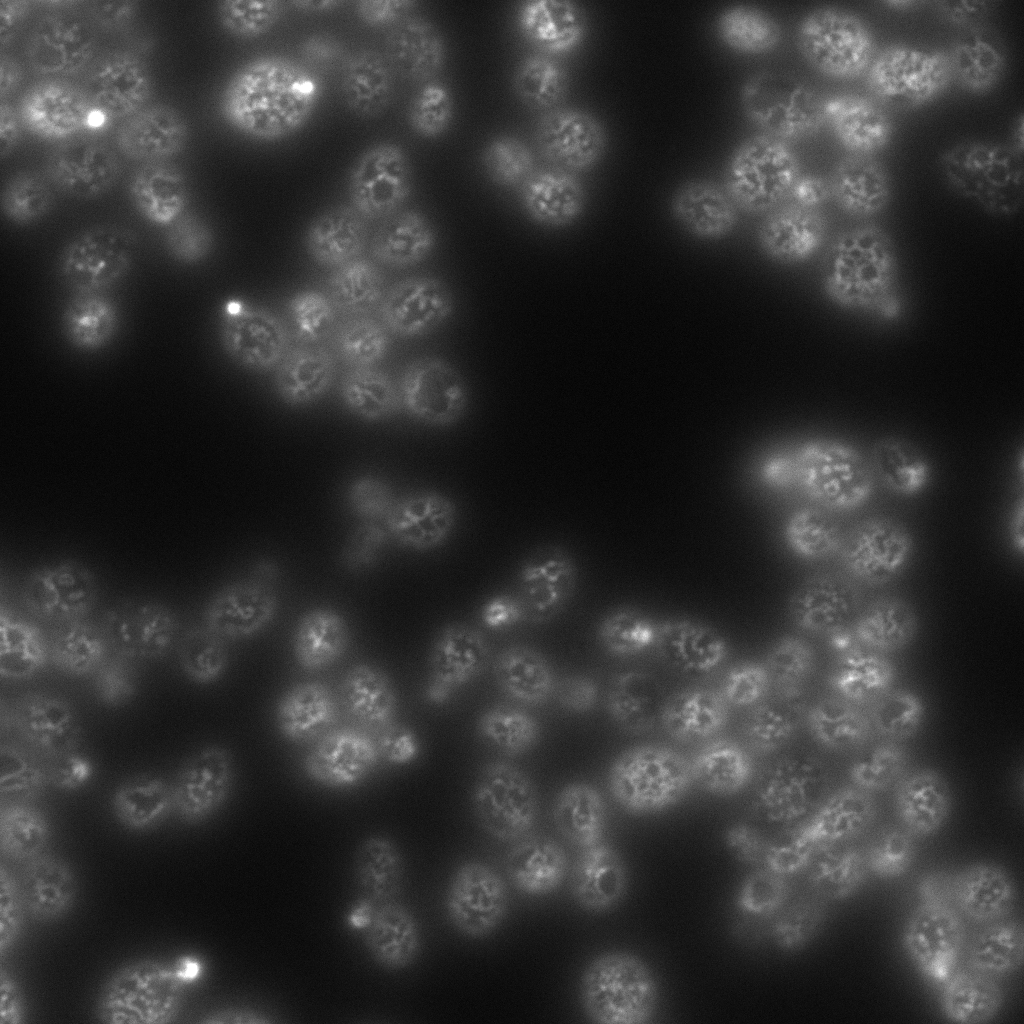

Supplement: Supplementary file 7 — Source data Fig. 6 [file 44318_2024_355_MOESM7_ESM.zip › Figure 6/6C/28 Cup1-Lro1* z8-1 (Rtn1-mCh) copy.tif]

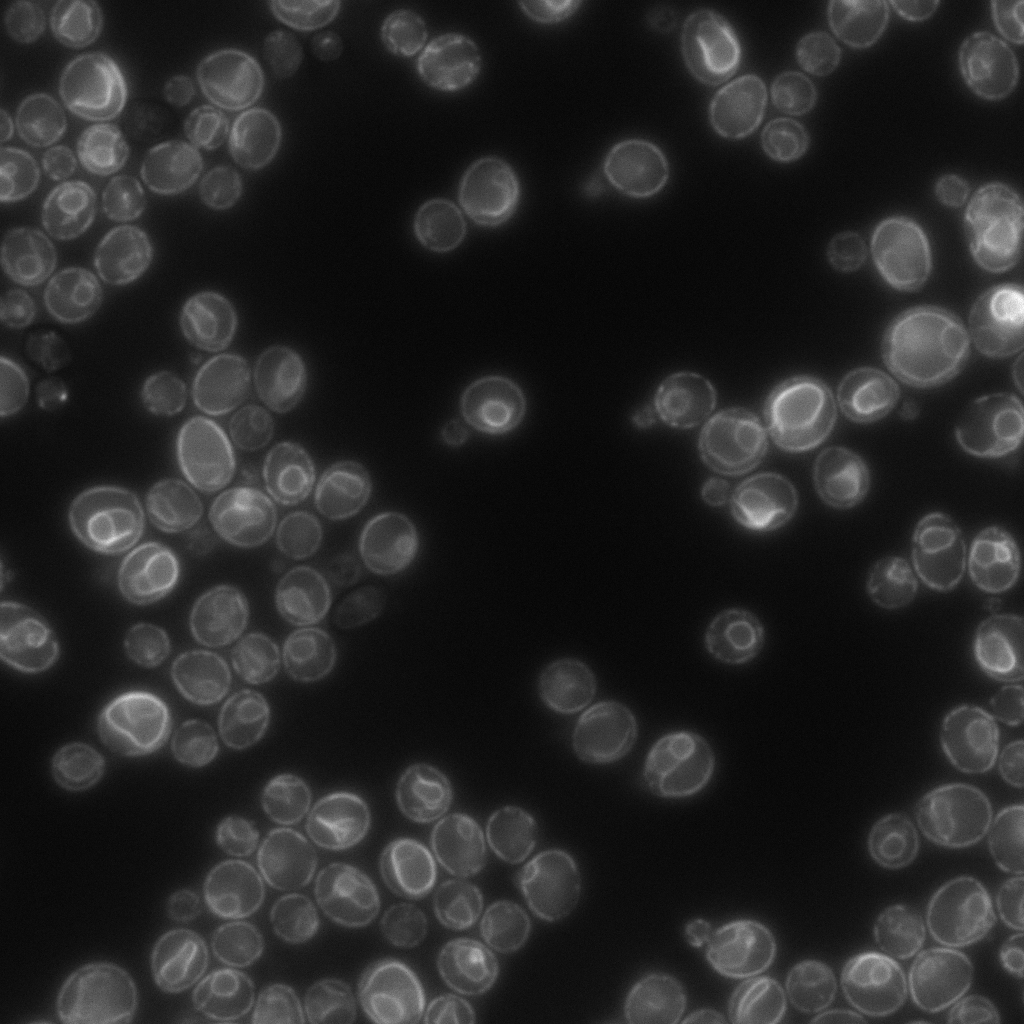

Supplement: Supplementary file 7 — Source data Fig. 6 [file 44318_2024_355_MOESM7_ESM.zip › Figure 6/6C/22 Cup1-Lro1*[S324A] z13 (Sec63-mNG) copy.tif]

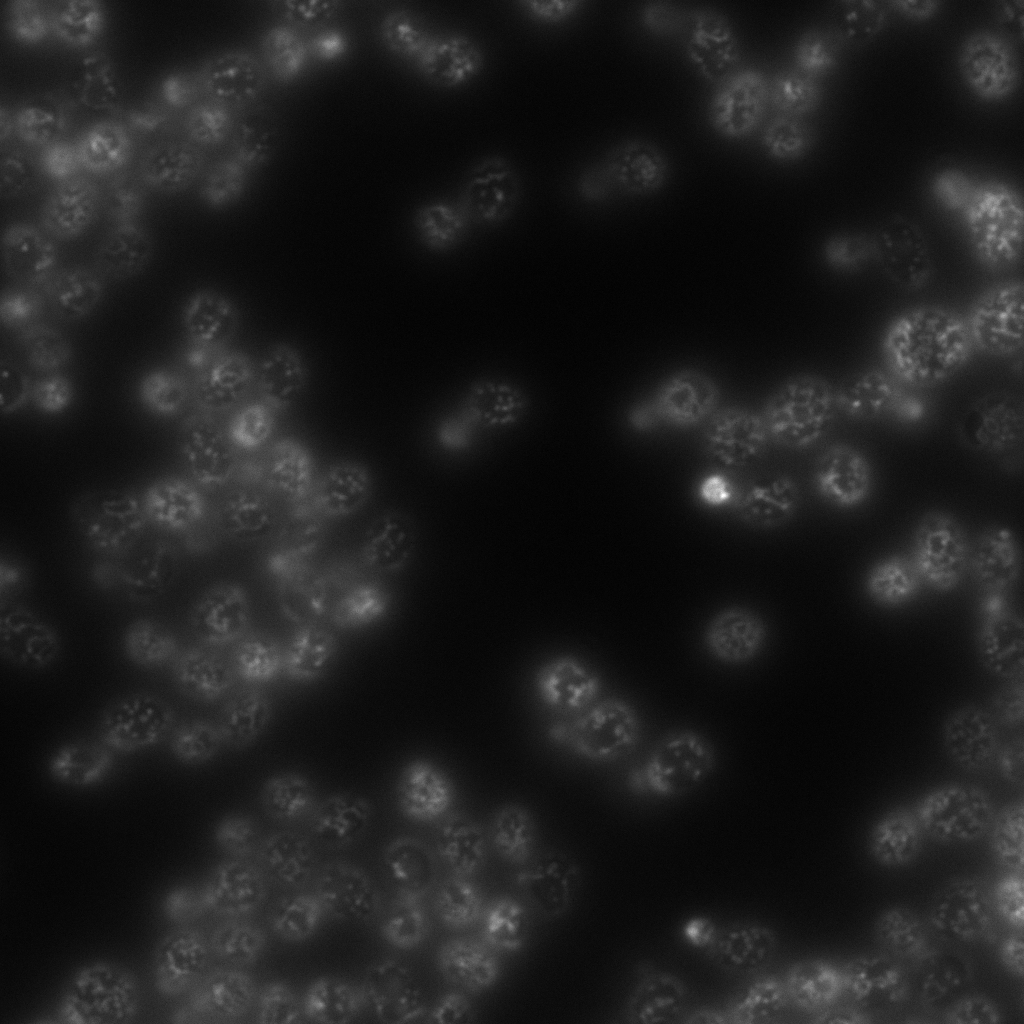

Supplement: Supplementary file 7 — Source data Fig. 6 [file 44318_2024_355_MOESM7_ESM.zip › Figure 6/6C/22 Cup1-Lro1*[S324A] z7 (Rtn1-mCh) copy.tif]

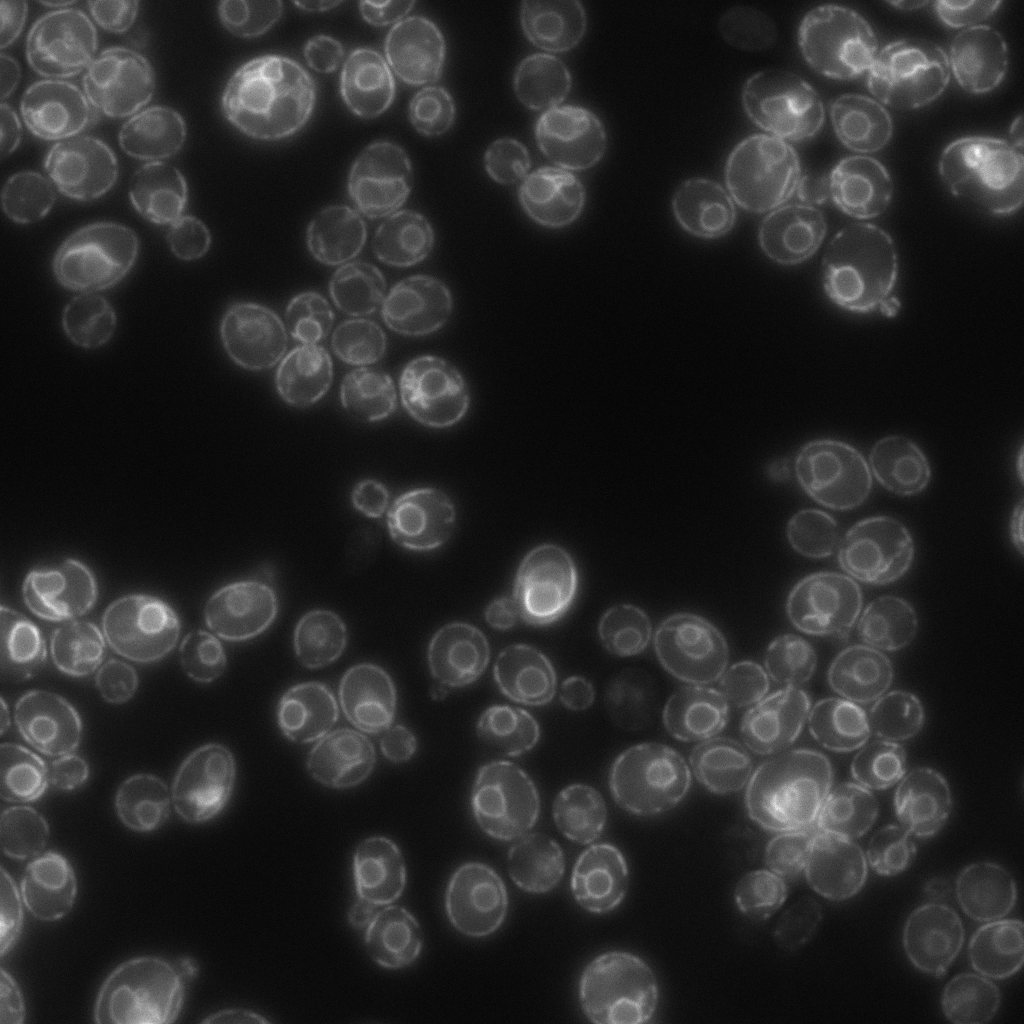

Supplement: Supplementary file 7 — Source data Fig. 6 [file 44318_2024_355_MOESM7_ESM.zip › Figure 6/6C/28 Cup1-Lro1* z16-1 (Sec63-mNG) copy.tif]

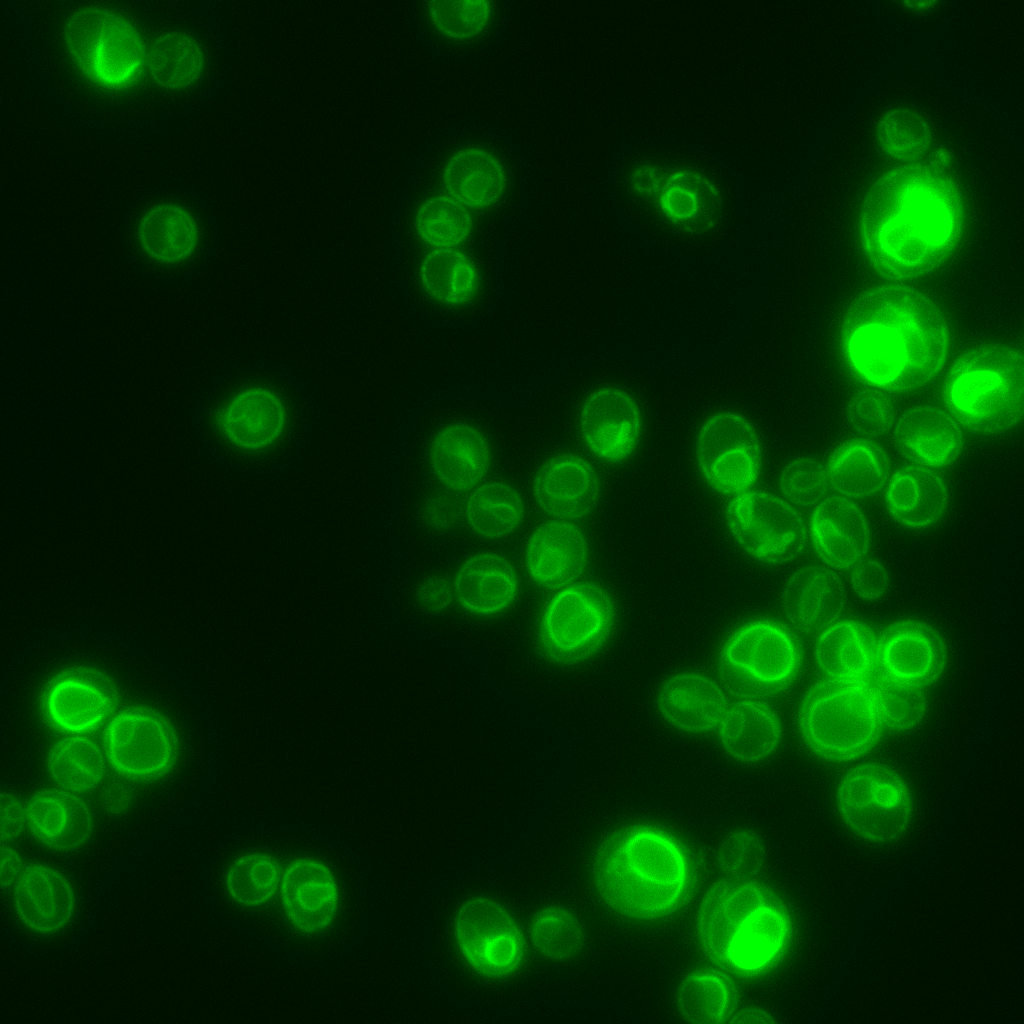

Supplement: Supplementary file 7 — Source data Fig. 6 [file 44318_2024_355_MOESM7_ESM.zip › Figure 6/6C/35 puraz16-1 (Sec63-mNG) copy.tif]

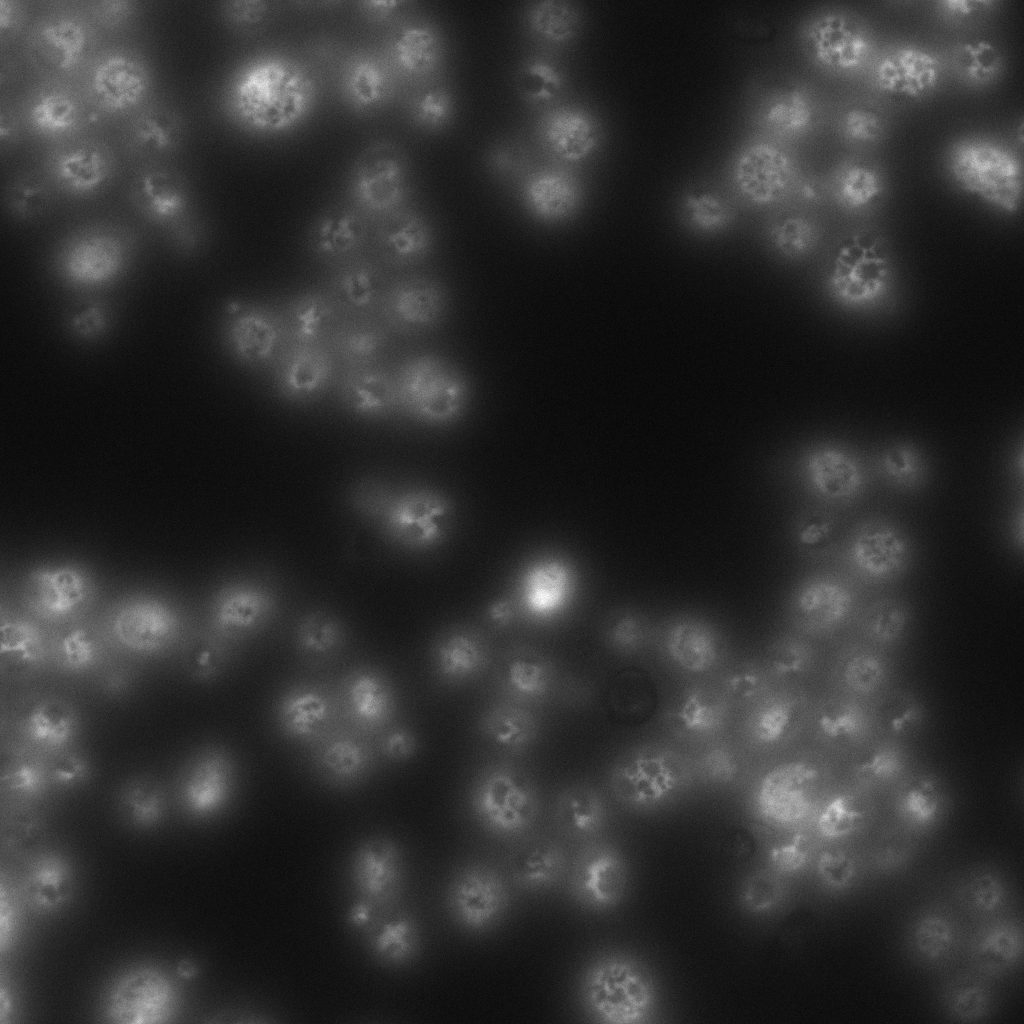

Supplement: Supplementary file 7 — Source data Fig. 6 [file 44318_2024_355_MOESM7_ESM.zip › Figure 6/6C/28 Cup1-Lro1* z8-1 (Sec63-mNG) copy.tif]

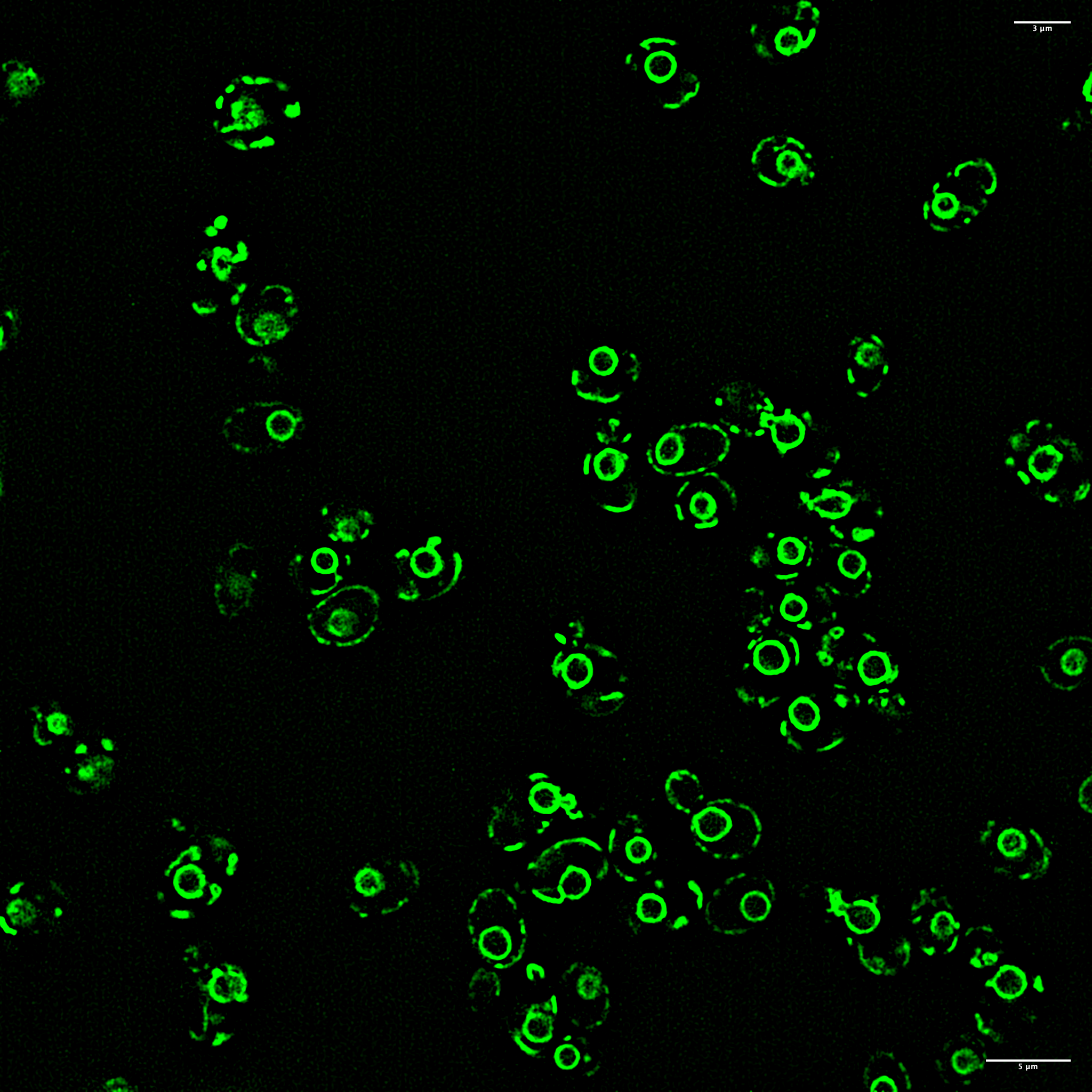

Supplement: Supplementary file 8 — Source data Fig. 7 [file 44318_2024_355_MOESM8_ESM.zip › Figure 7/7D/Image 6 (Lro1* Pah1-7A).tif]

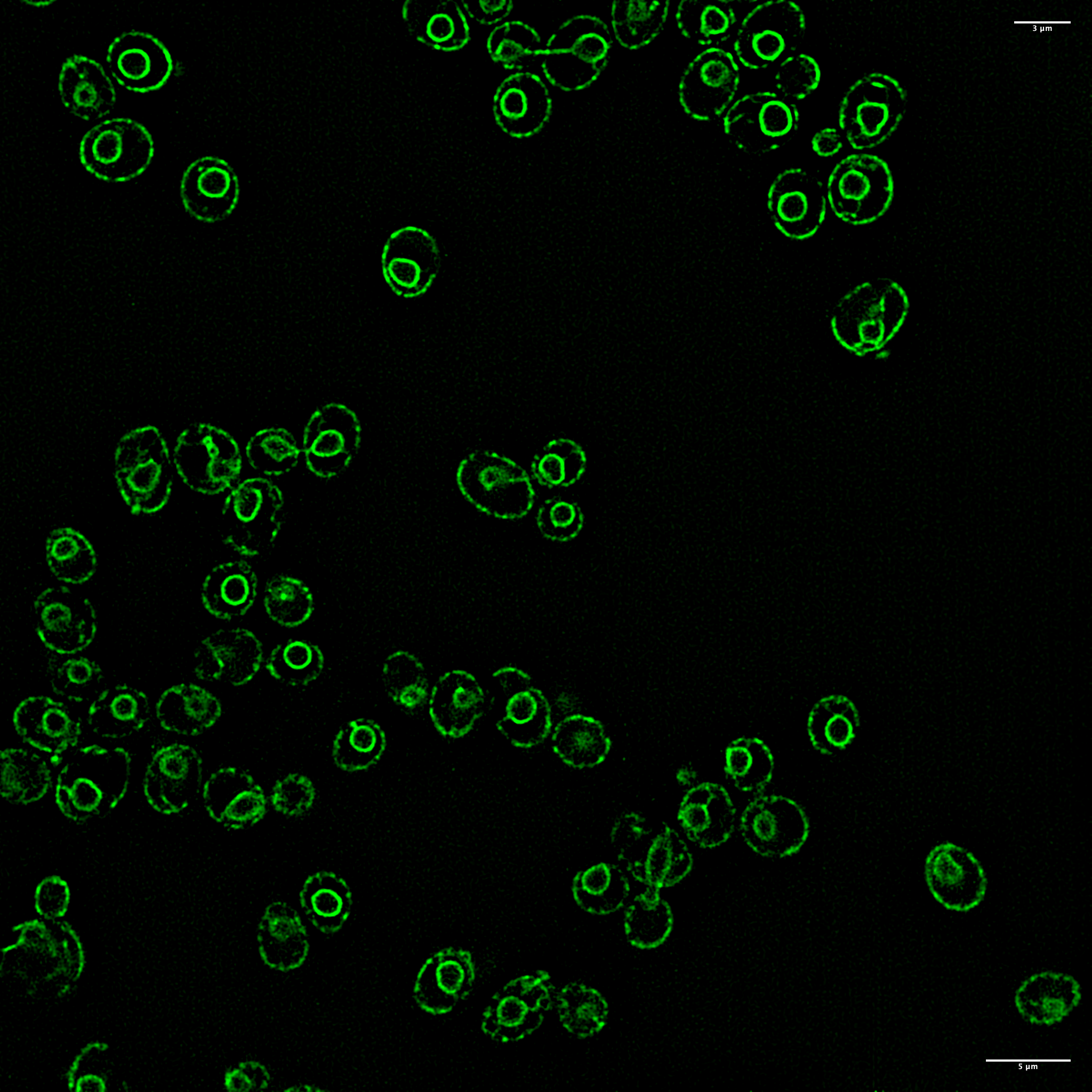

Supplement: Supplementary file 8 — Source data Fig. 7 [file 44318_2024_355_MOESM8_ESM.zip › Figure 7/7D/Image 16 (Lro1 Vector).tif]

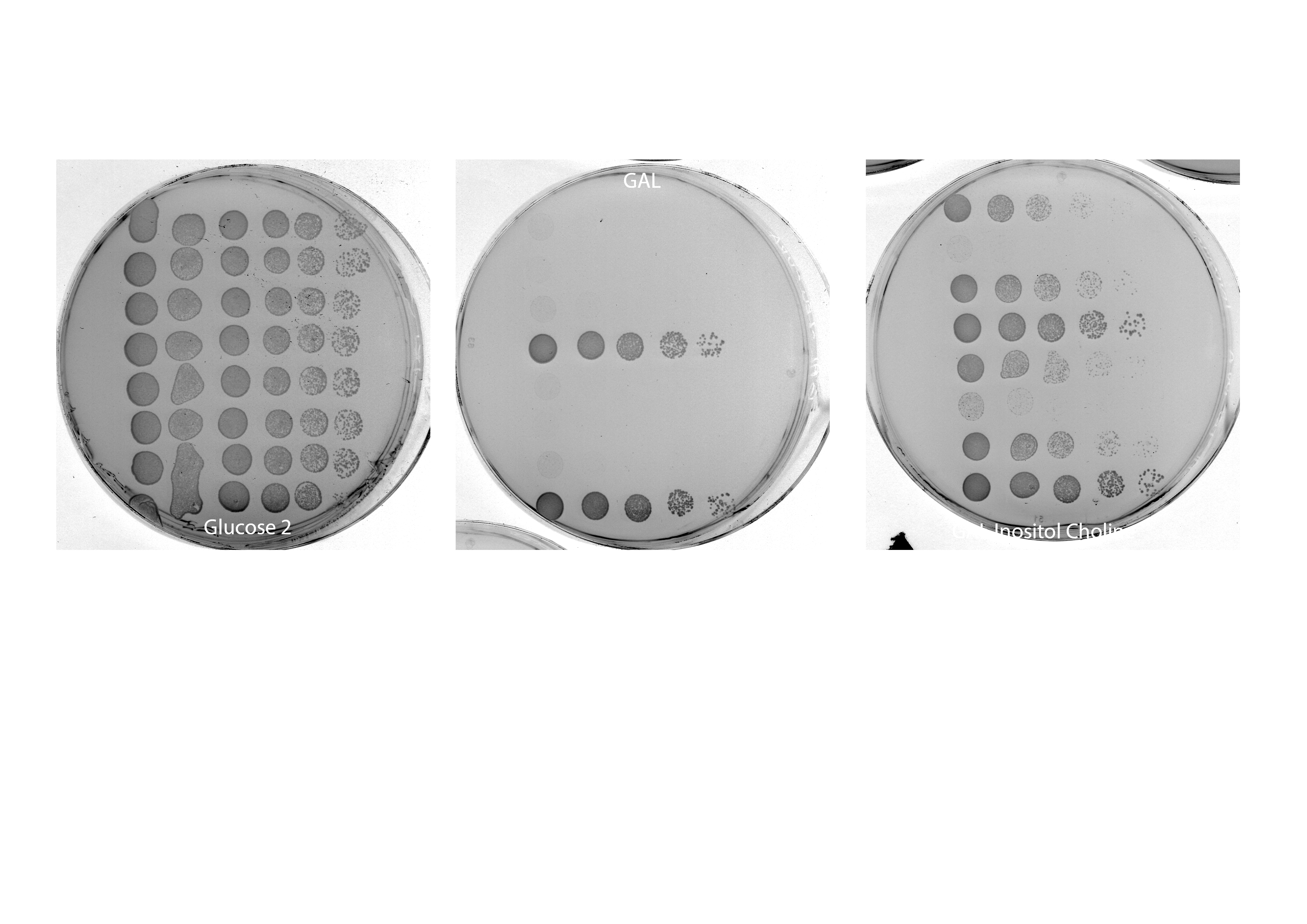

Supplement: Supplementary file 8 — Source data Fig. 7 [file 44318_2024_355_MOESM8_ESM.zip › Figure 7/7C/Spot growth assay (Gluc Gal).tif]

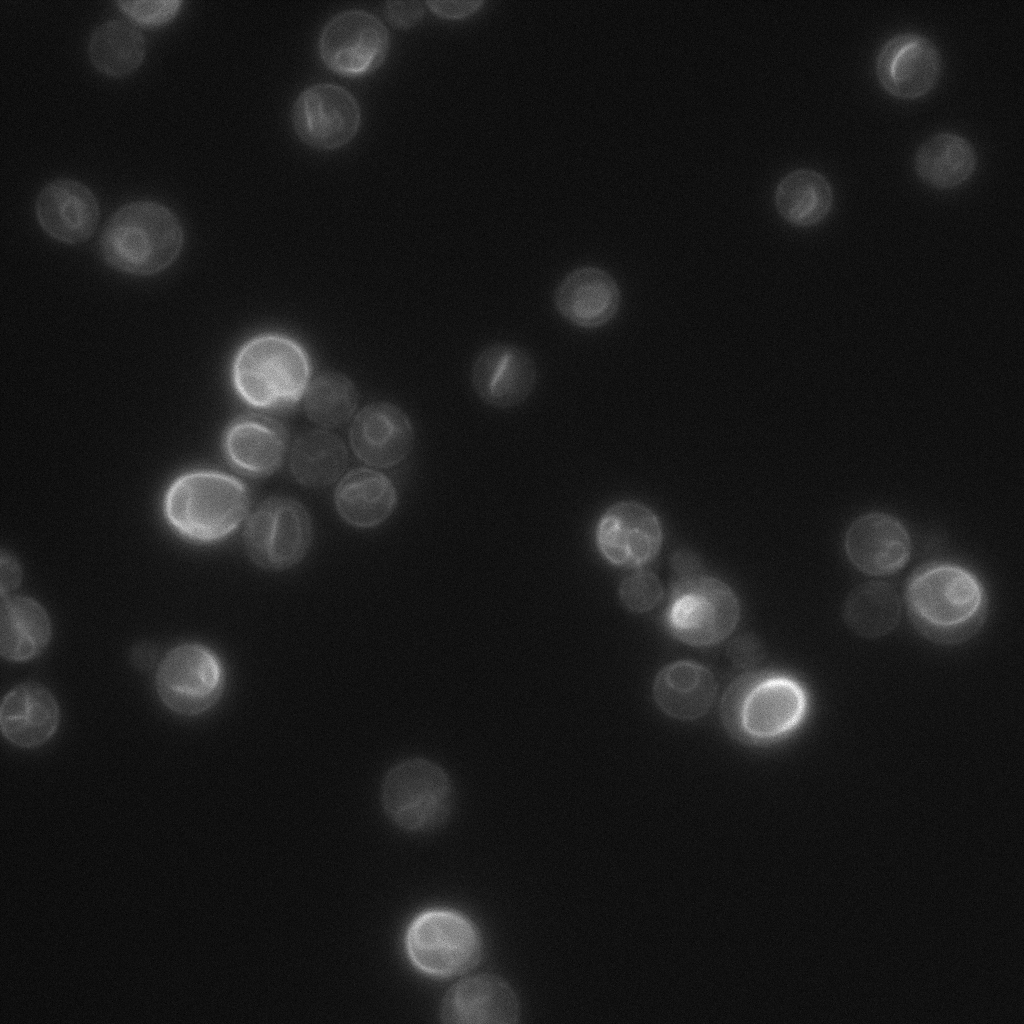

Supplement: Supplementary file 8 — Source data Fig. 7 [file 44318_2024_355_MOESM8_ESM.zip › Figure 7/7B/Vector.tif]

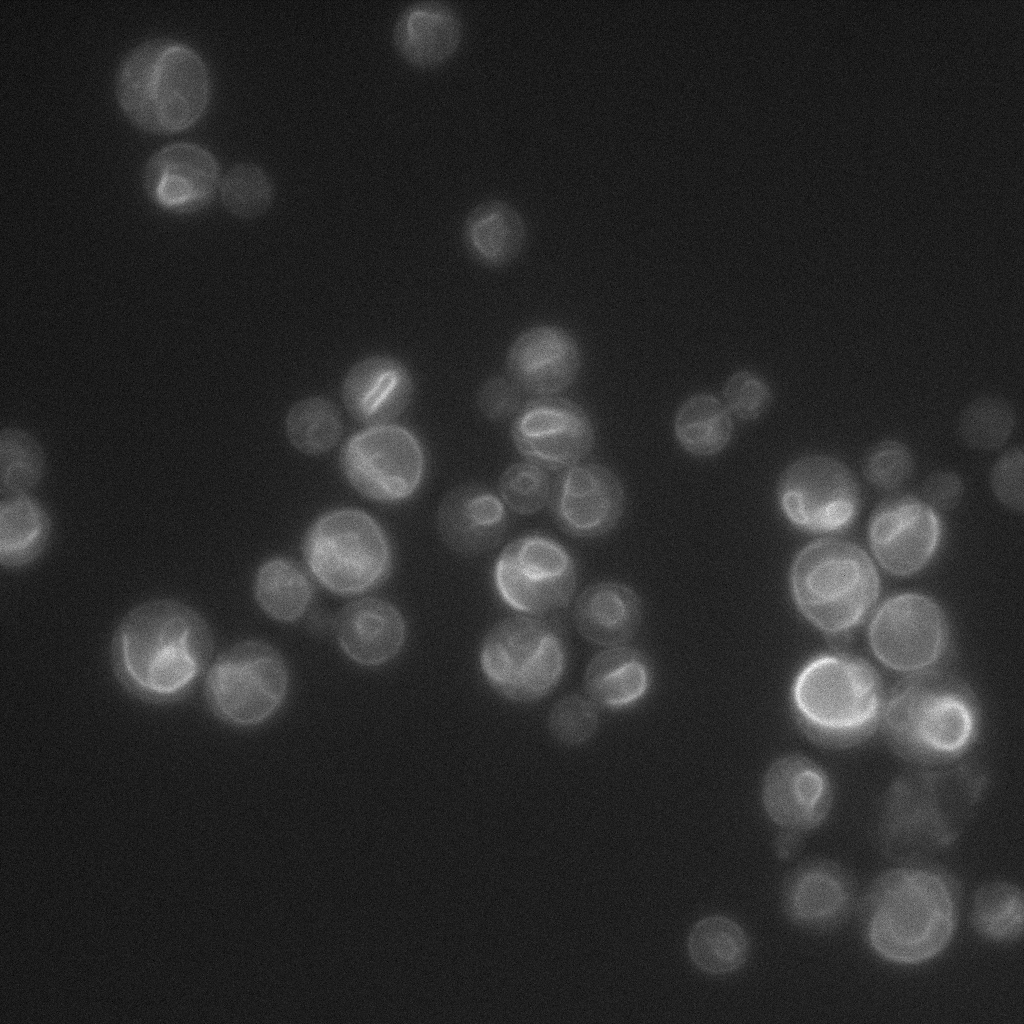

Supplement: Supplementary file 8 — Source data Fig. 7 [file 44318_2024_355_MOESM8_ESM.zip › Figure 7/7B/Cup1-Lro1*.tif]

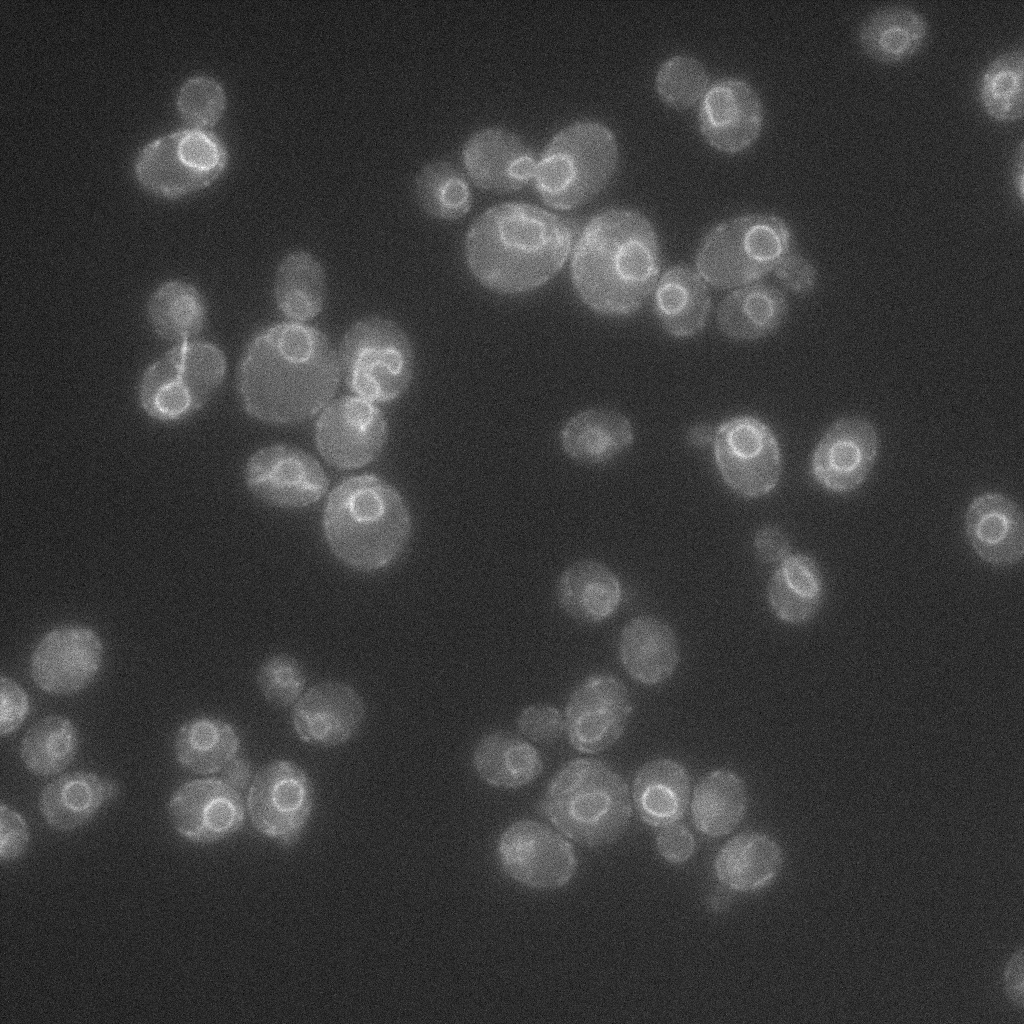

Supplement: Supplementary file 8 — Source data Fig. 7 [file 44318_2024_355_MOESM8_ESM.zip › Figure 7/7B/Pah1.tif]

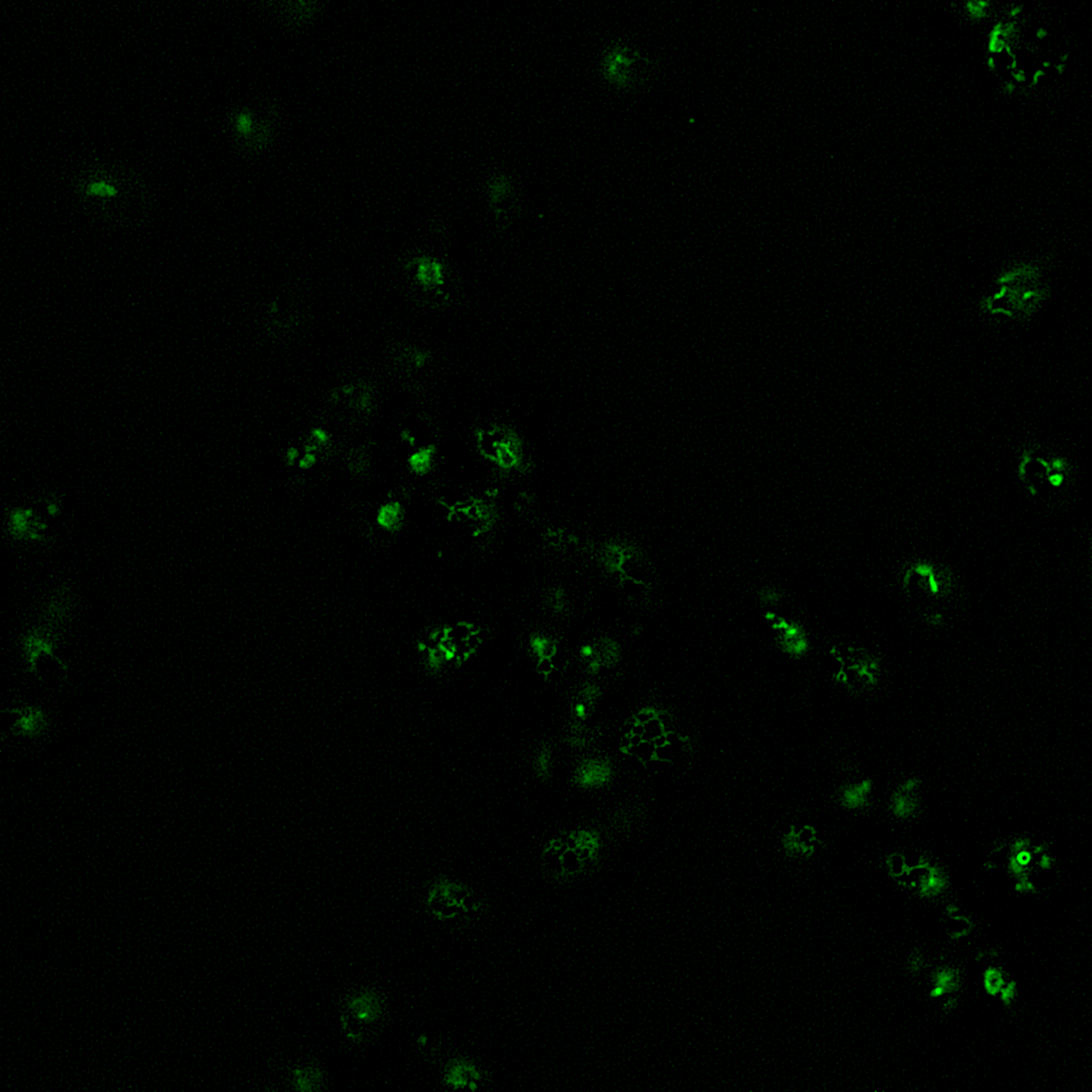

Supplement: Supplementary file 8 — Source data Fig. 7 [file 44318_2024_355_MOESM8_ESM.zip › Figure 7/7E/Image 5z17 (Lro1* Pah1-7A) cortical.tif]

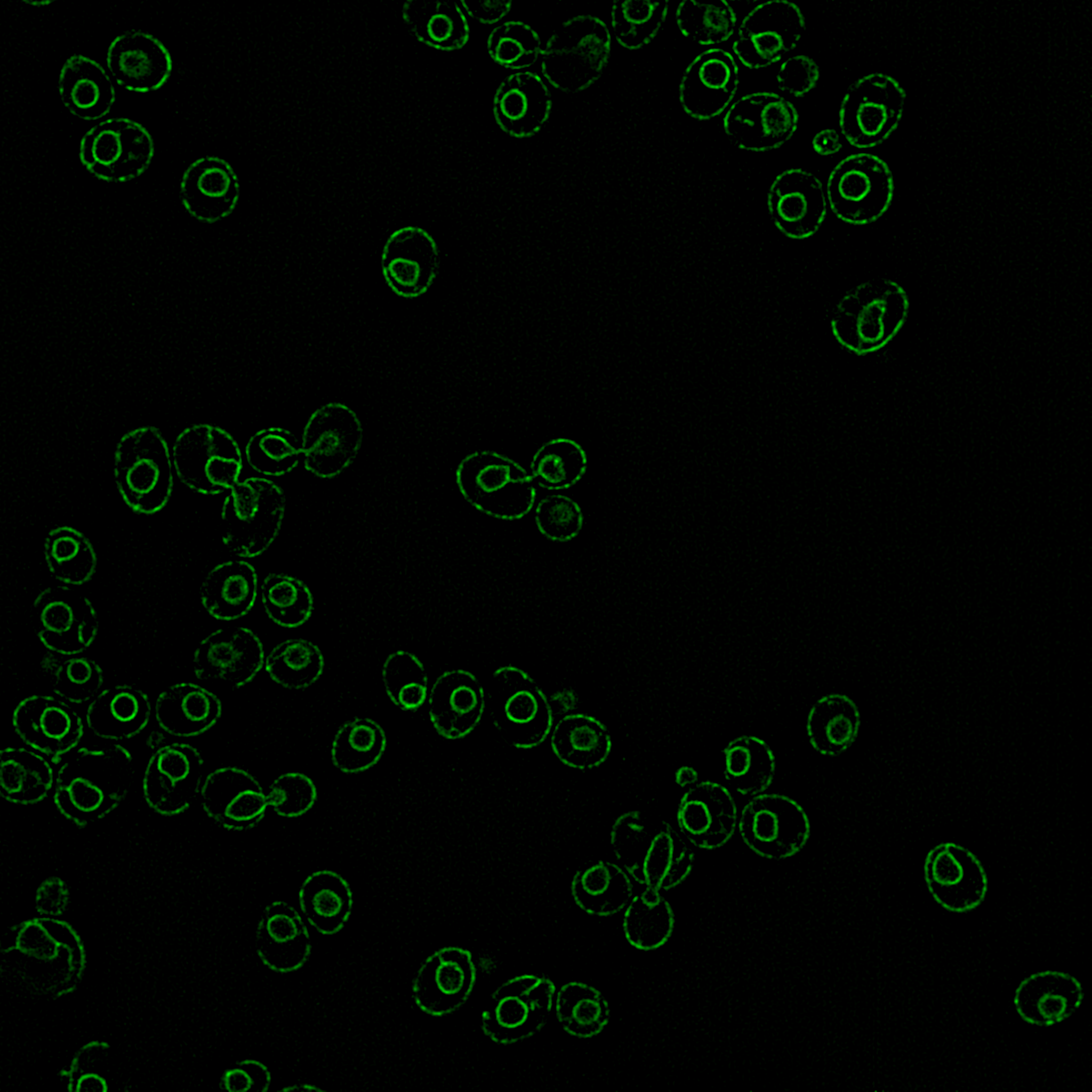

Supplement: Supplementary file 8 — Source data Fig. 7 [file 44318_2024_355_MOESM8_ESM.zip › Figure 7/7E/Image 16z25 (Lro1 vector) middle .tif]

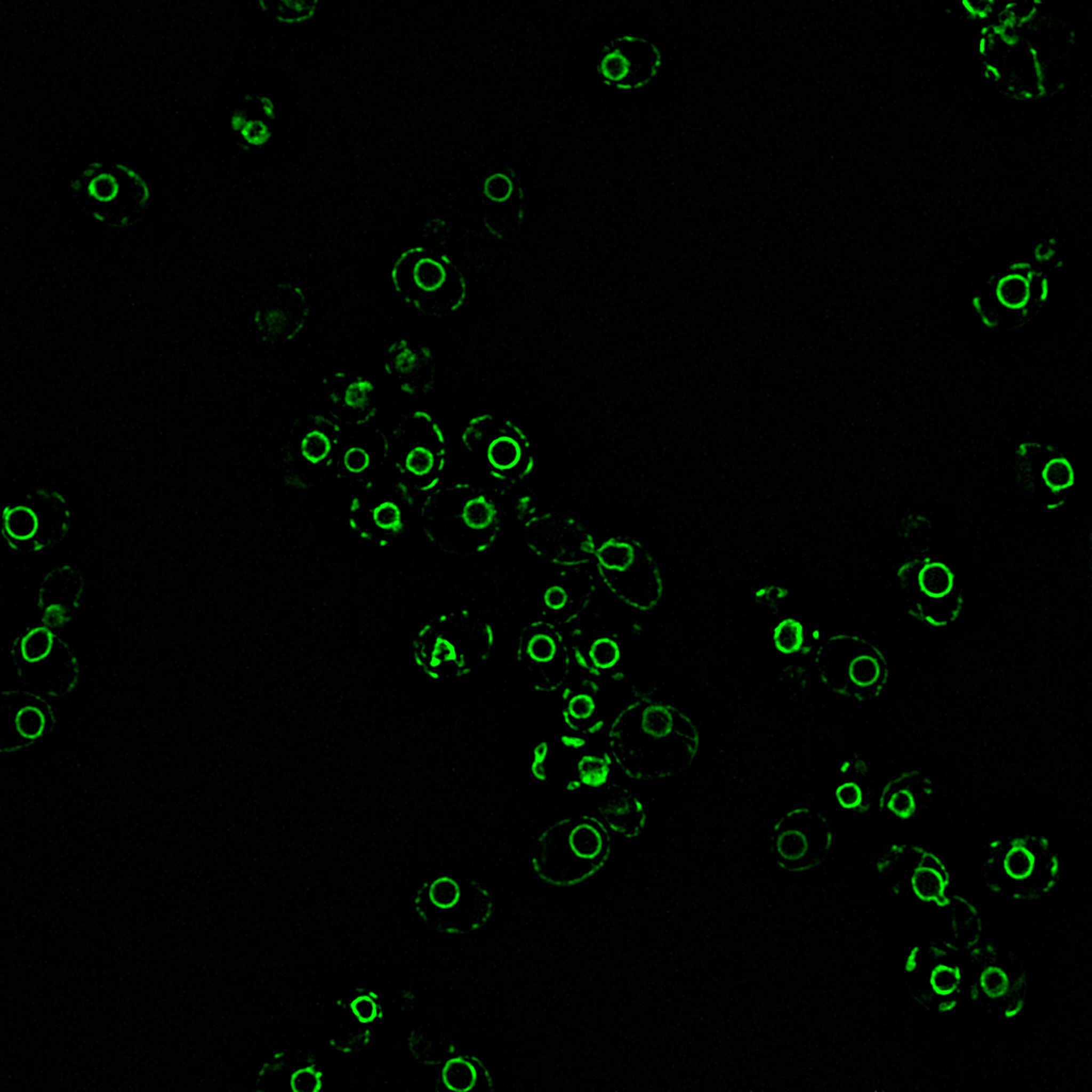

Supplement: Supplementary file 8 — Source data Fig. 7 [file 44318_2024_355_MOESM8_ESM.zip › Figure 7/7E/Image 5z31 (Lro1* Pah1-7A) middle.tif]

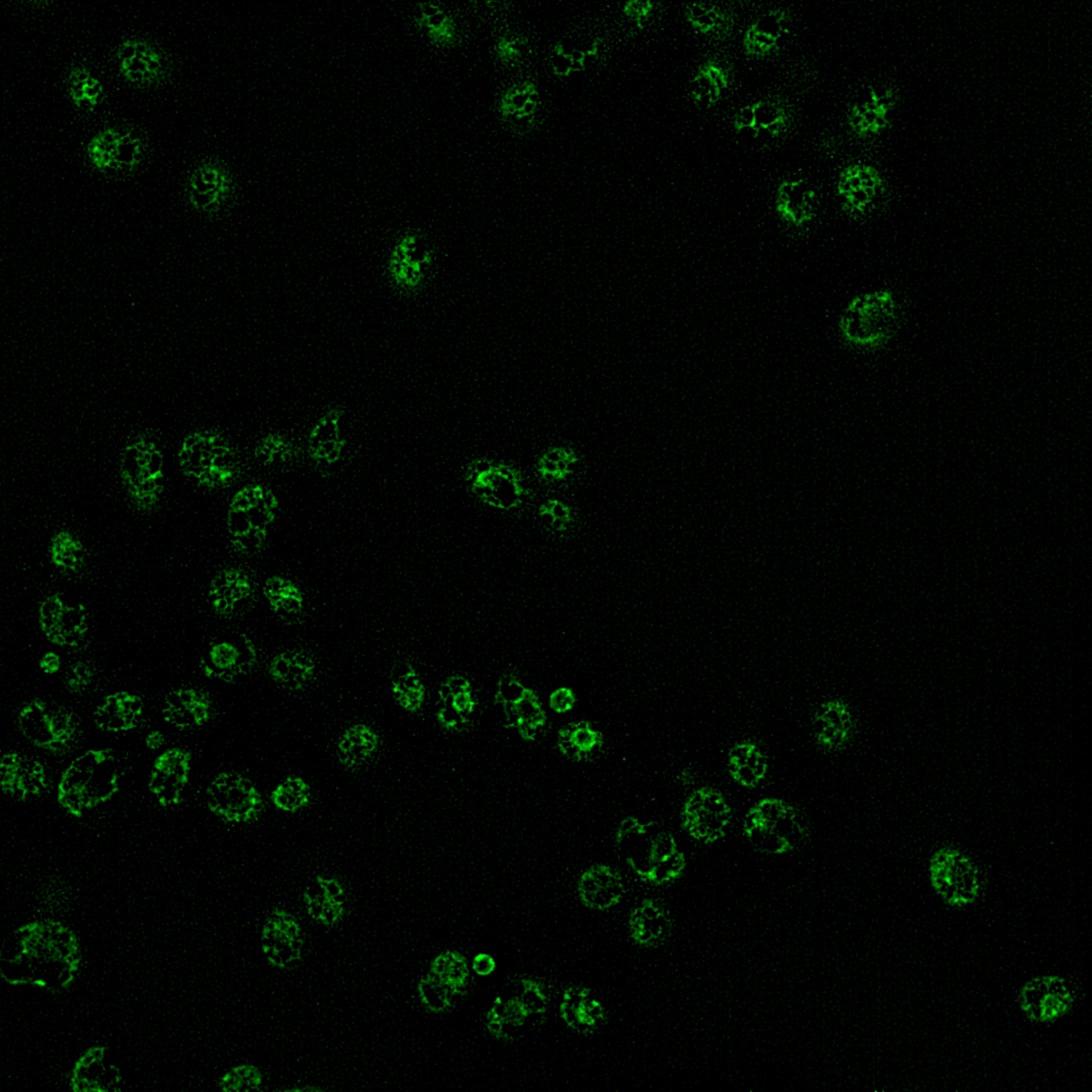

Supplement: Supplementary file 8 — Source data Fig. 7 [file 44318_2024_355_MOESM8_ESM.zip › Figure 7/7E/Image 16z12 (Lro1 vector) cortical.tif]

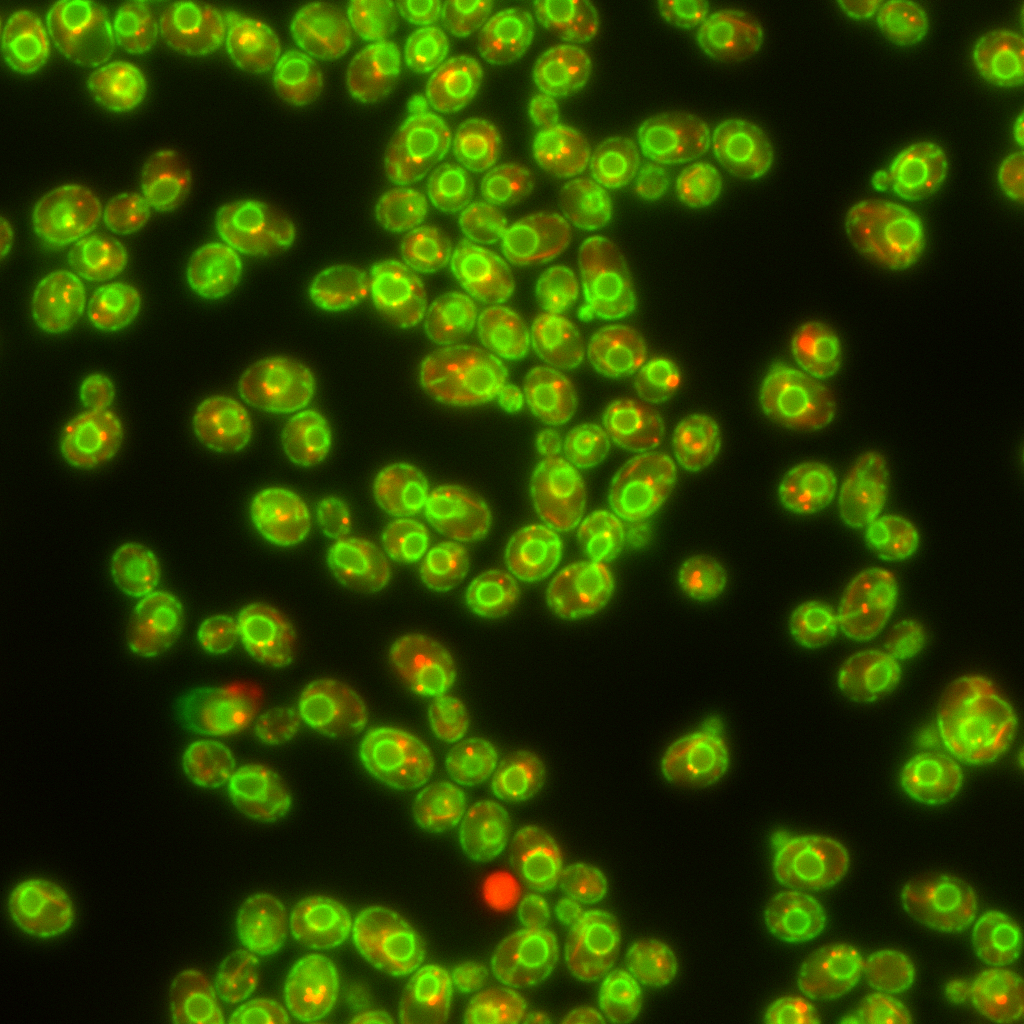

Supplement: Supplementary file 8 — Source data Fig. 7 [file 44318_2024_355_MOESM8_ESM.zip › Figure 7/7G/Image 1.12 (Lro1 vector).tif]

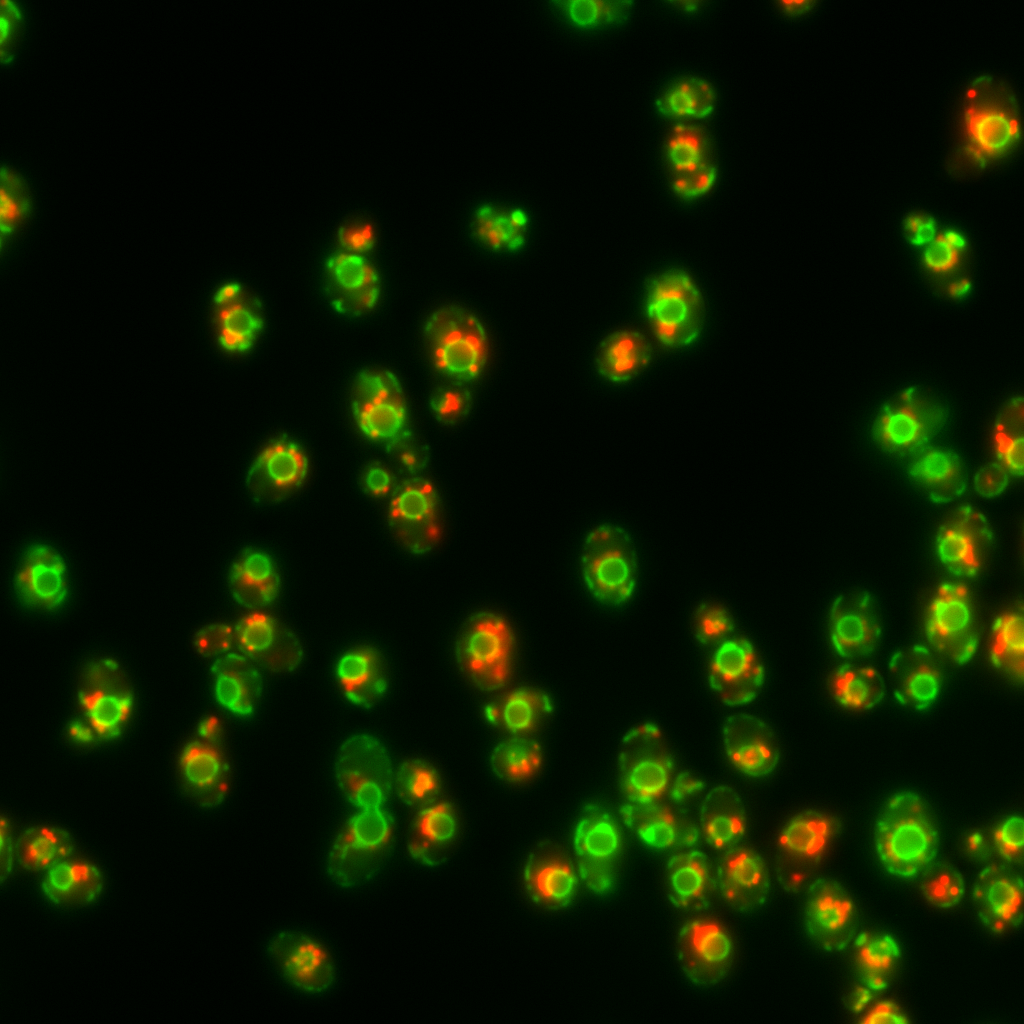

Supplement: Supplementary file 8 — Source data Fig. 7 [file 44318_2024_355_MOESM8_ESM.zip › Figure 7/7G/Image 4.38 (Lro1* Pah1-7A).tif]

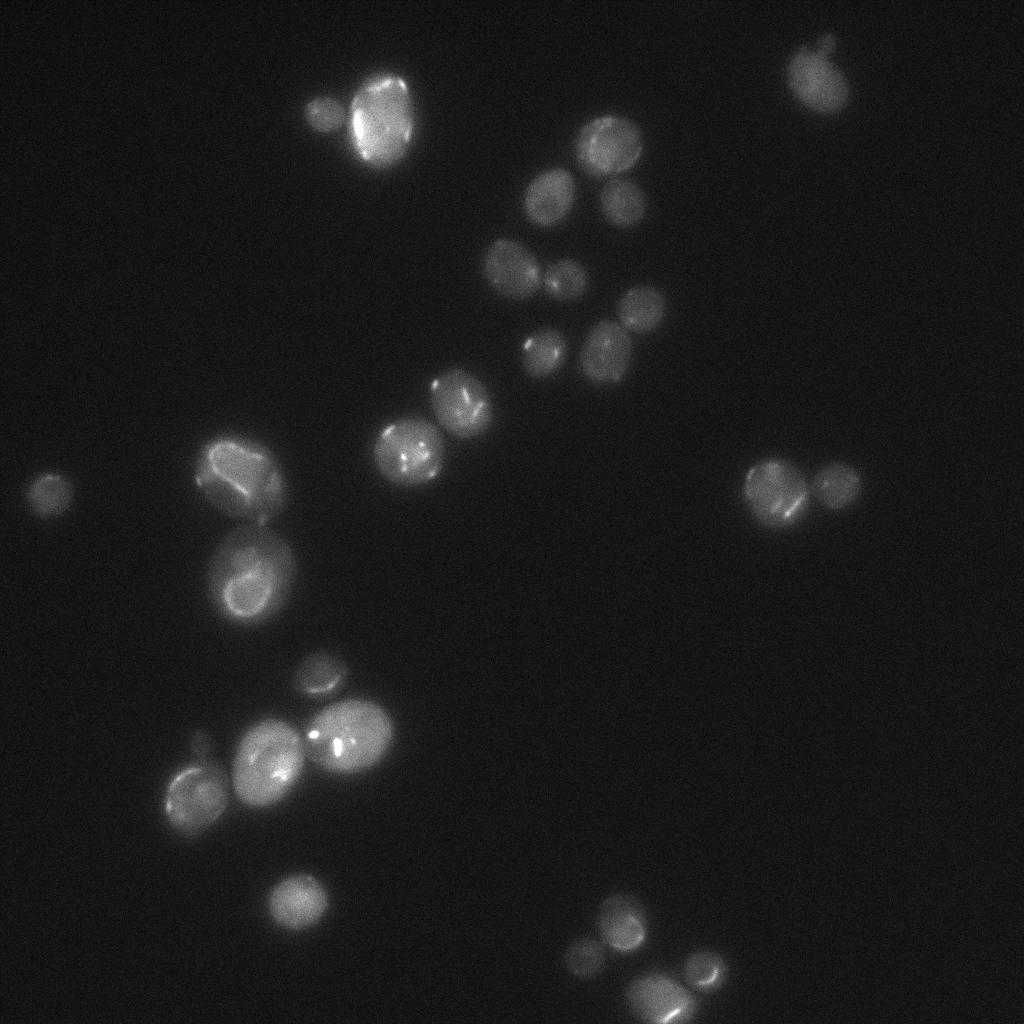

Supplement: Supplementary file 8 — Source data Fig. 7 [file 44318_2024_355_MOESM8_ESM.zip › Figure 7/7A/Galactose (Pah1-GFP).tif]

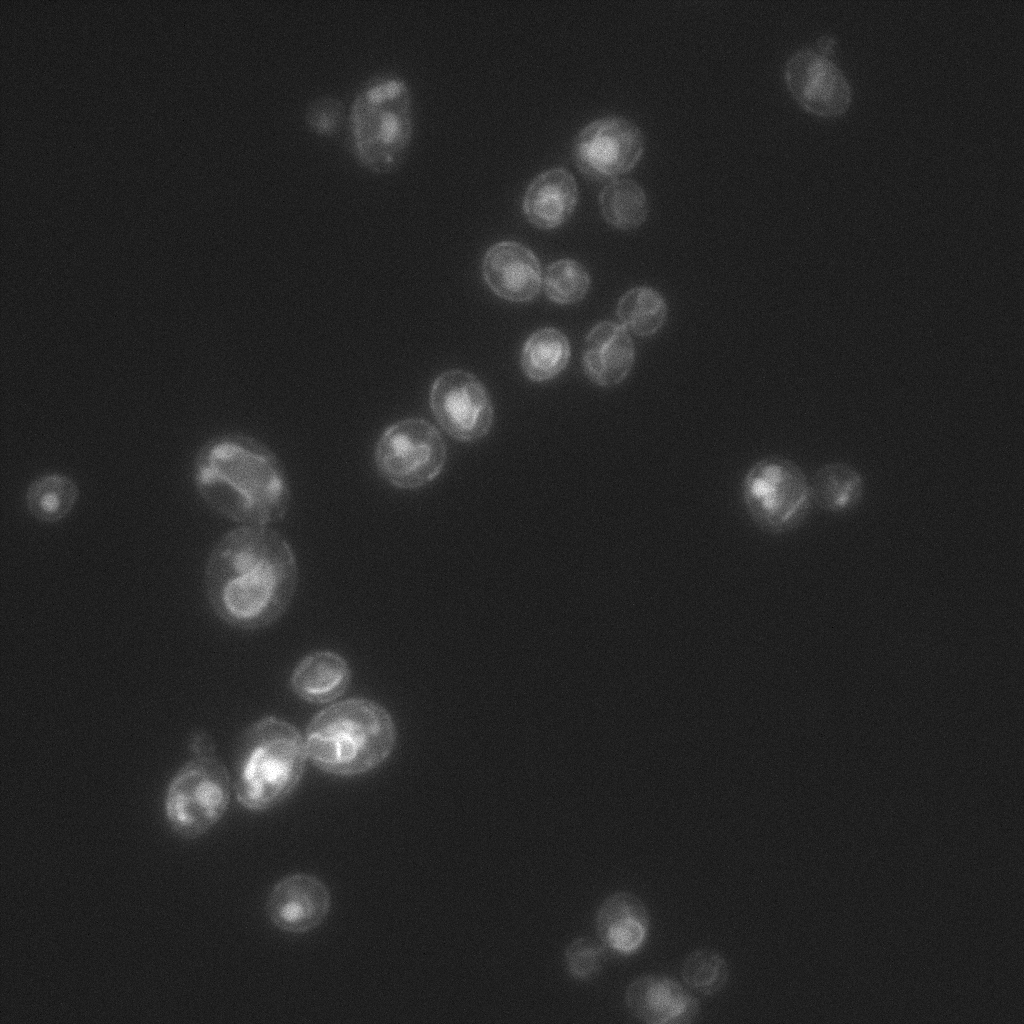

Supplement: Supplementary file 8 — Source data Fig. 7 [file 44318_2024_355_MOESM8_ESM.zip › Figure 7/7A/Galactose (Sec63-mCh).tif]

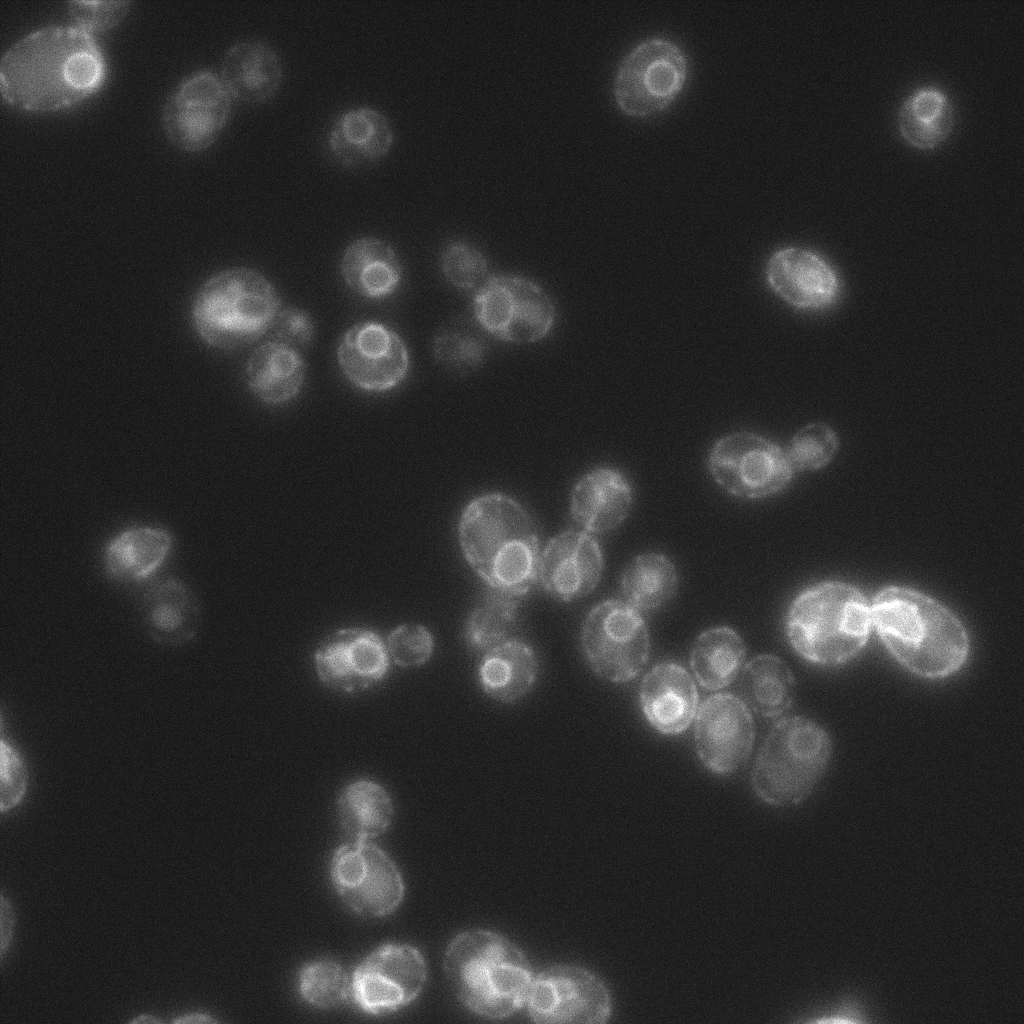

Supplement: Supplementary file 8 — Source data Fig. 7 [file 44318_2024_355_MOESM8_ESM.zip › Figure 7/7A/Glucose (Sec63-mCh).tif]

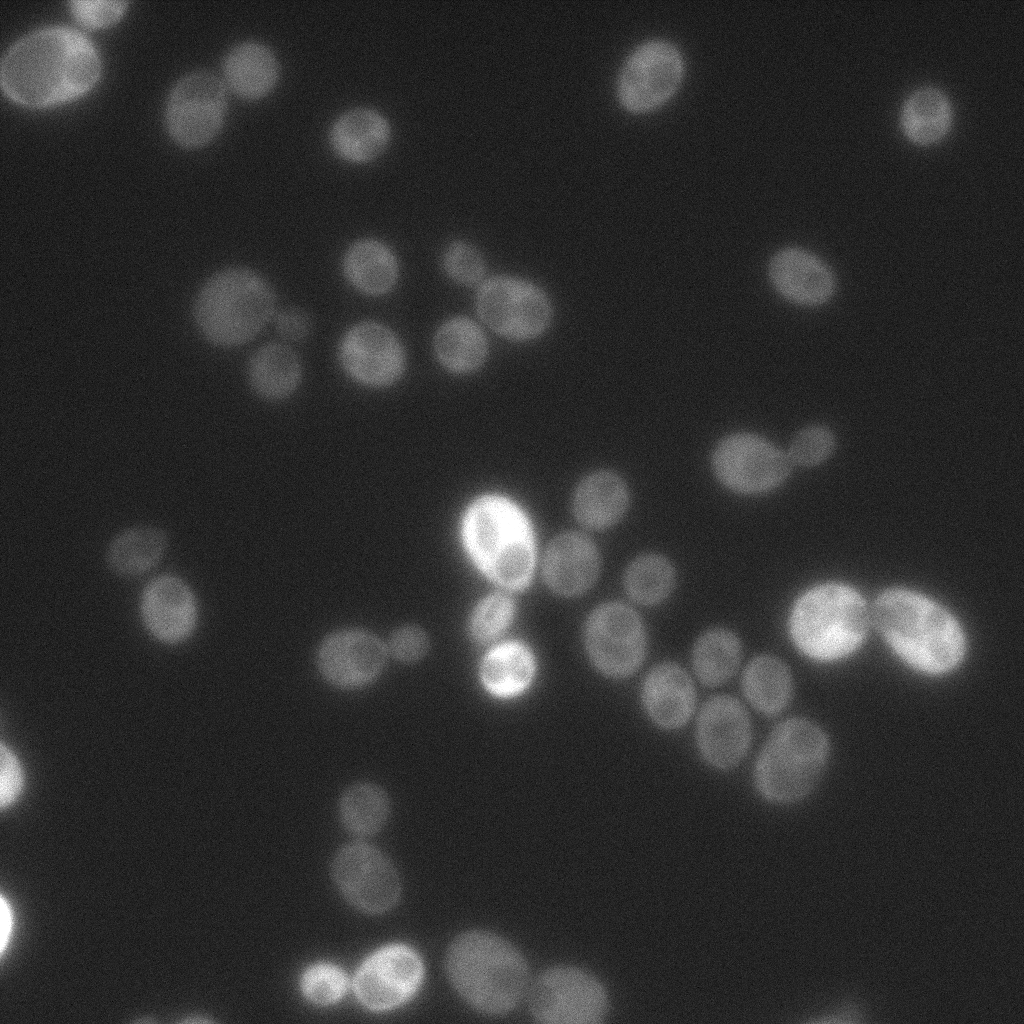

Supplement: Supplementary file 8 — Source data Fig. 7 [file 44318_2024_355_MOESM8_ESM.zip › Figure 7/7A/Glucose (Pah1-GFP).tif]

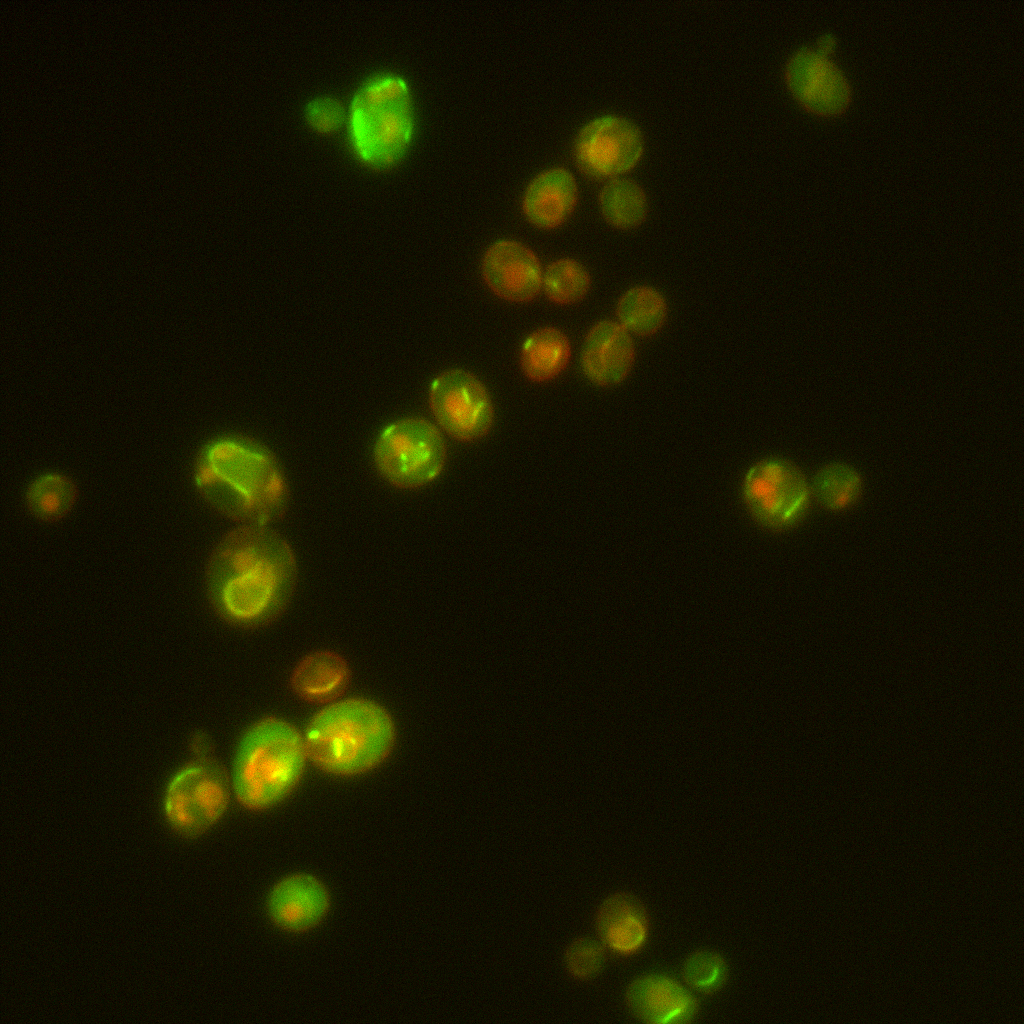

Supplement: Supplementary file 8 — Source data Fig. 7 [file 44318_2024_355_MOESM8_ESM.zip › Figure 7/7A/Galactose (Merge).tif]

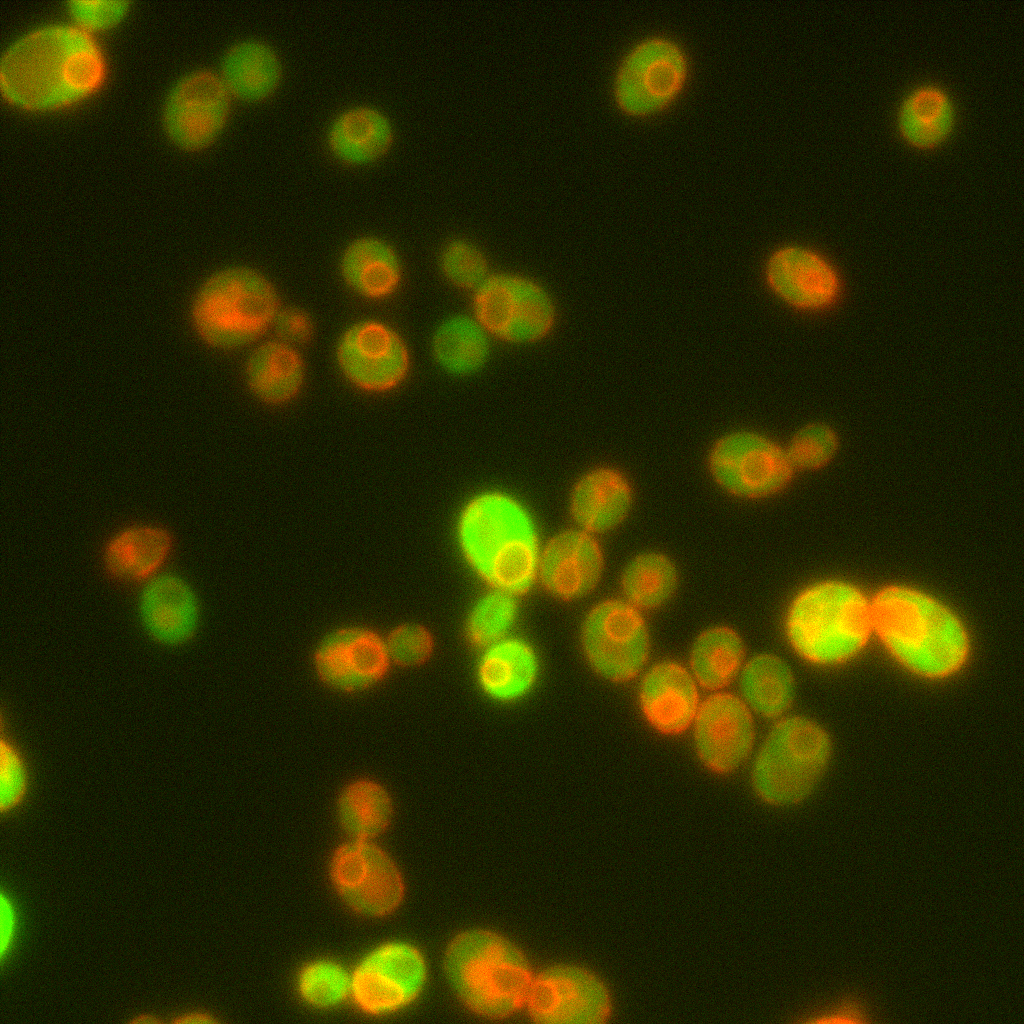

Supplement: Supplementary file 8 — Source data Fig. 7 [file 44318_2024_355_MOESM8_ESM.zip › Figure 7/7A/Glucose (Merge).tif]

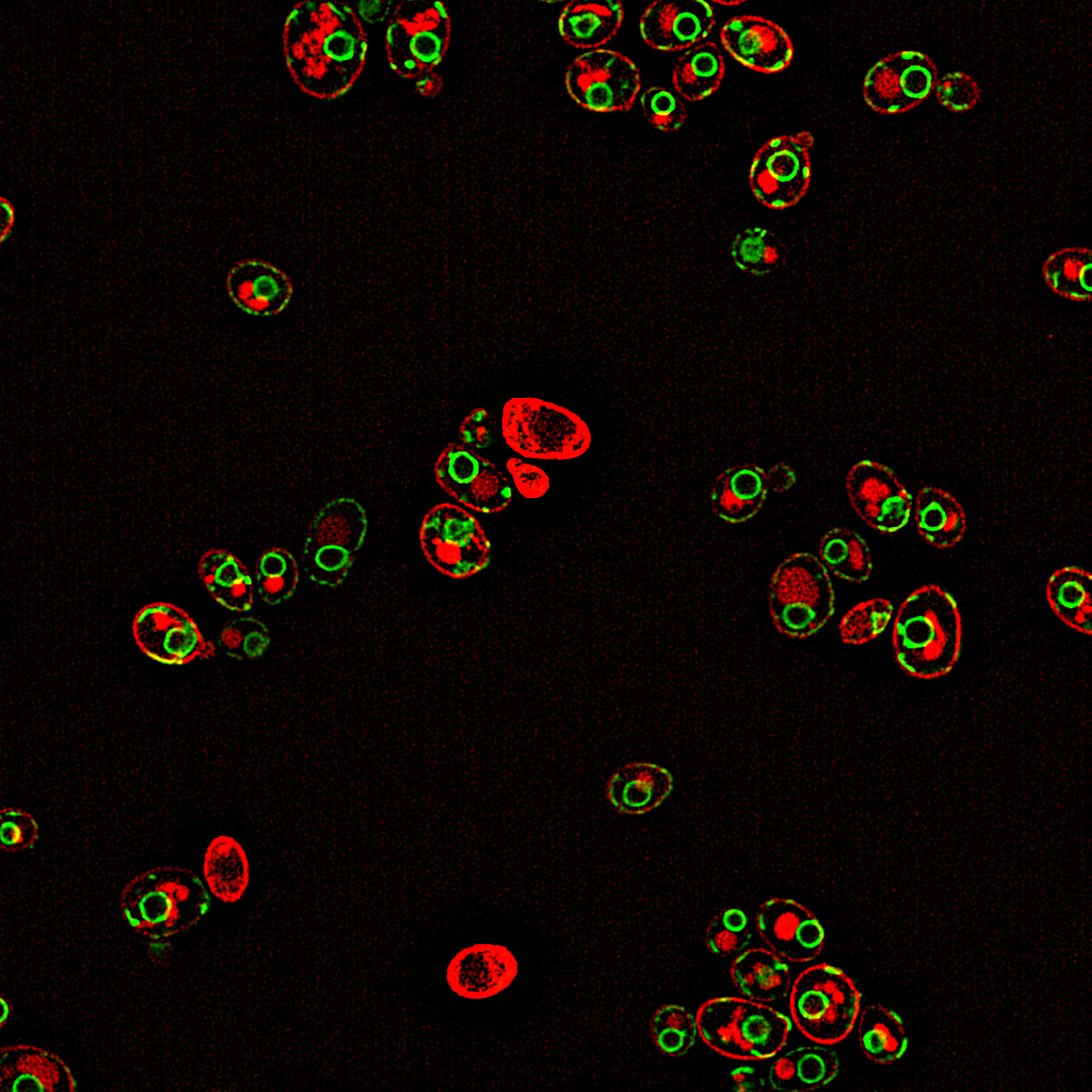

Supplement: Supplementary file 8 — Source data Fig. 7 [file 44318_2024_355_MOESM8_ESM.zip › Figure 7/7H/Merge Lro1* Pah1-7A.tif]

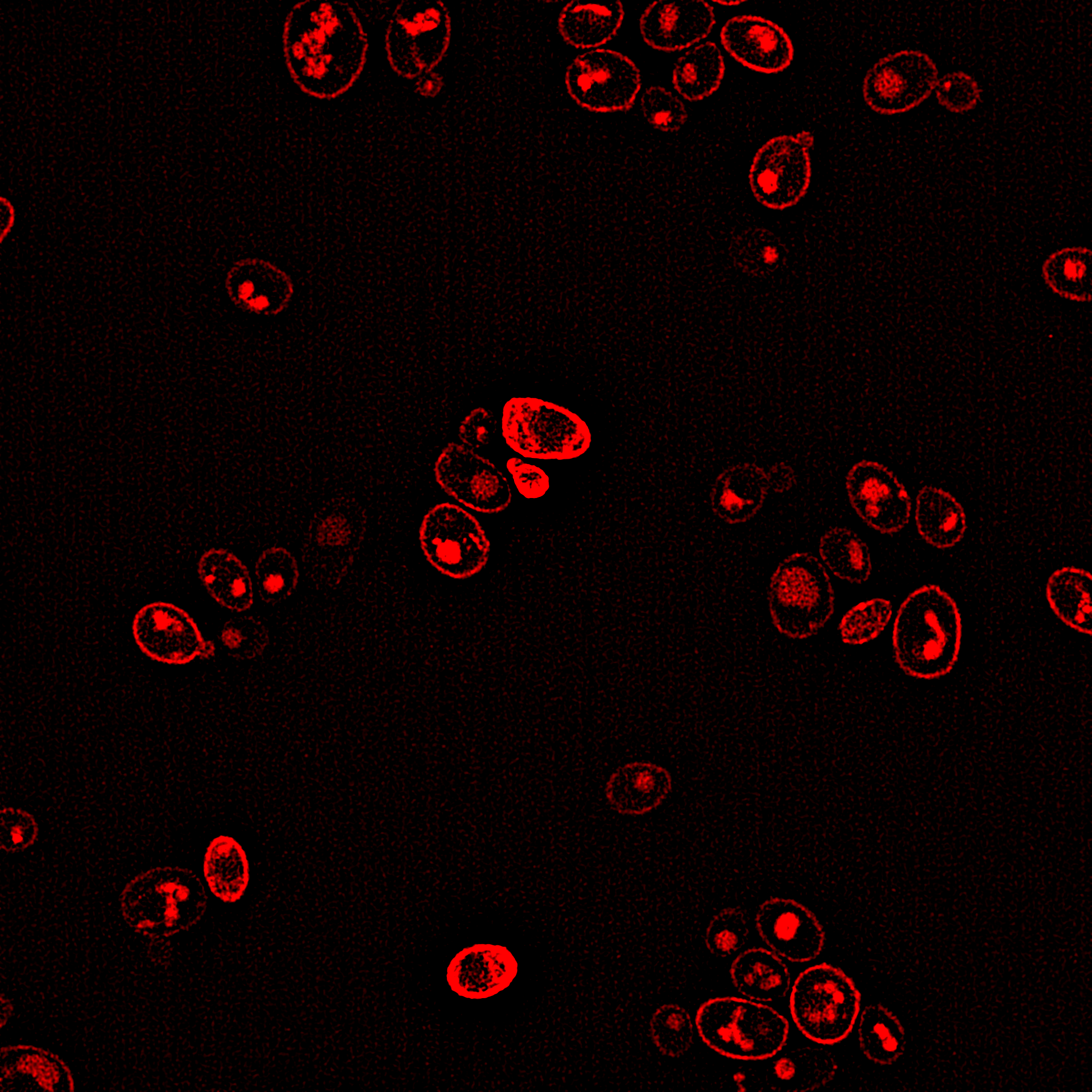

Supplement: Supplementary file 8 — Source data Fig. 7 [file 44318_2024_355_MOESM8_ESM.zip › Figure 7/7H/Image 8z22 Lro1* Pah1-7A (Psr1 [1-28]-mCh).tif]

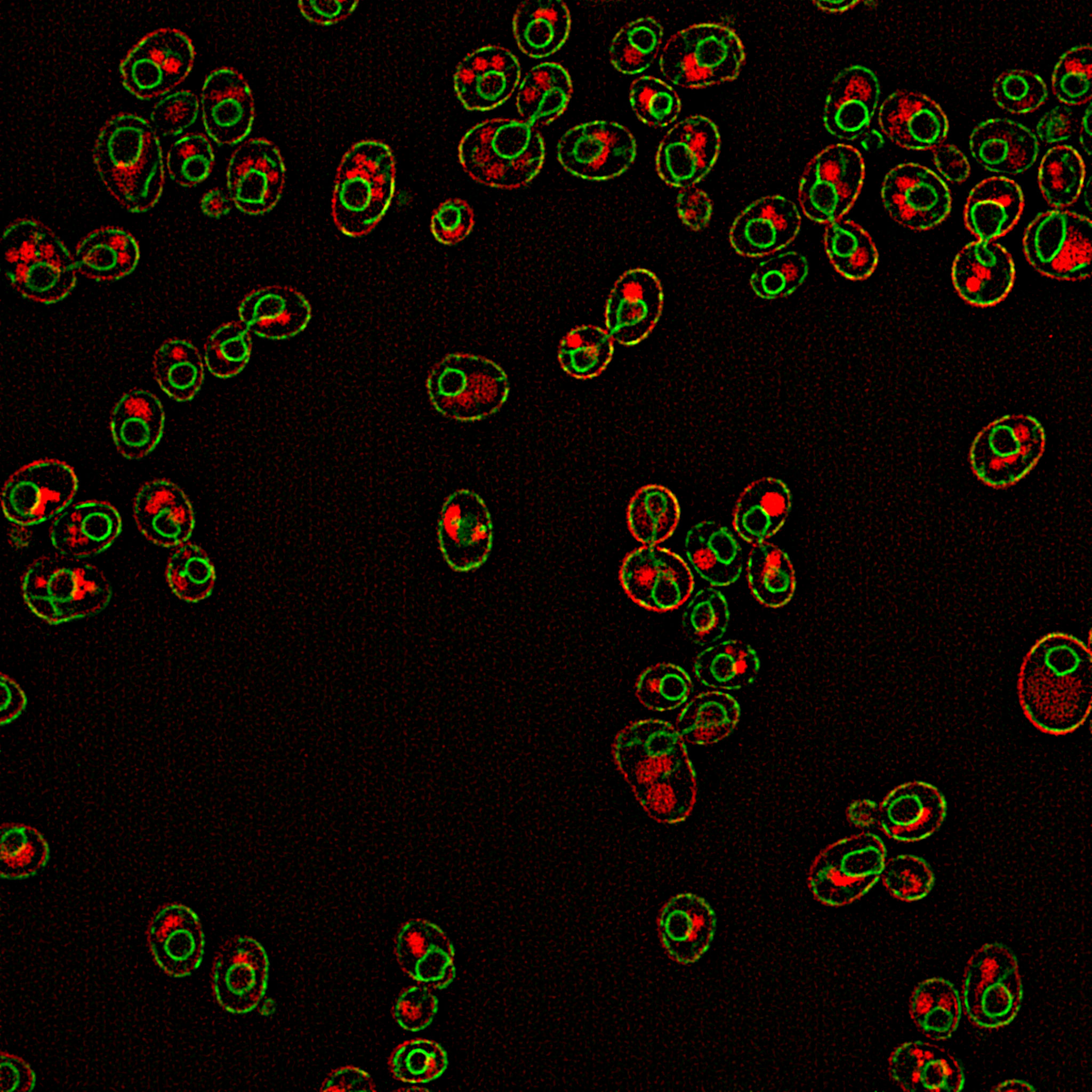

Supplement: Supplementary file 8 — Source data Fig. 7 [file 44318_2024_355_MOESM8_ESM.zip › Figure 7/7H/Merge Lro1 vector.tif]

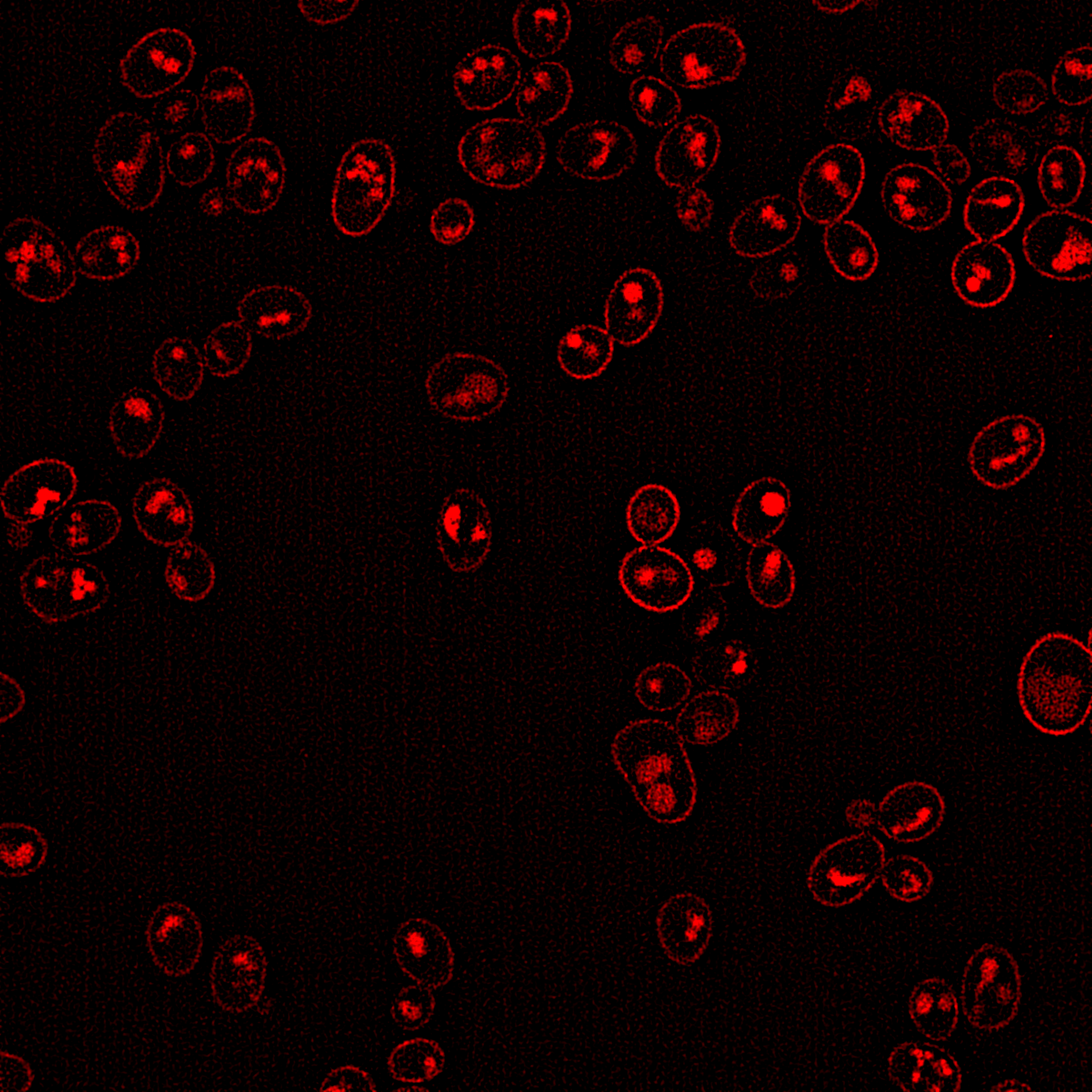

Supplement: Supplementary file 8 — Source data Fig. 7 [file 44318_2024_355_MOESM8_ESM.zip › Figure 7/7H/Image 3z21 Lro1 vector (Psr1 [1-28]-mCh).tif]

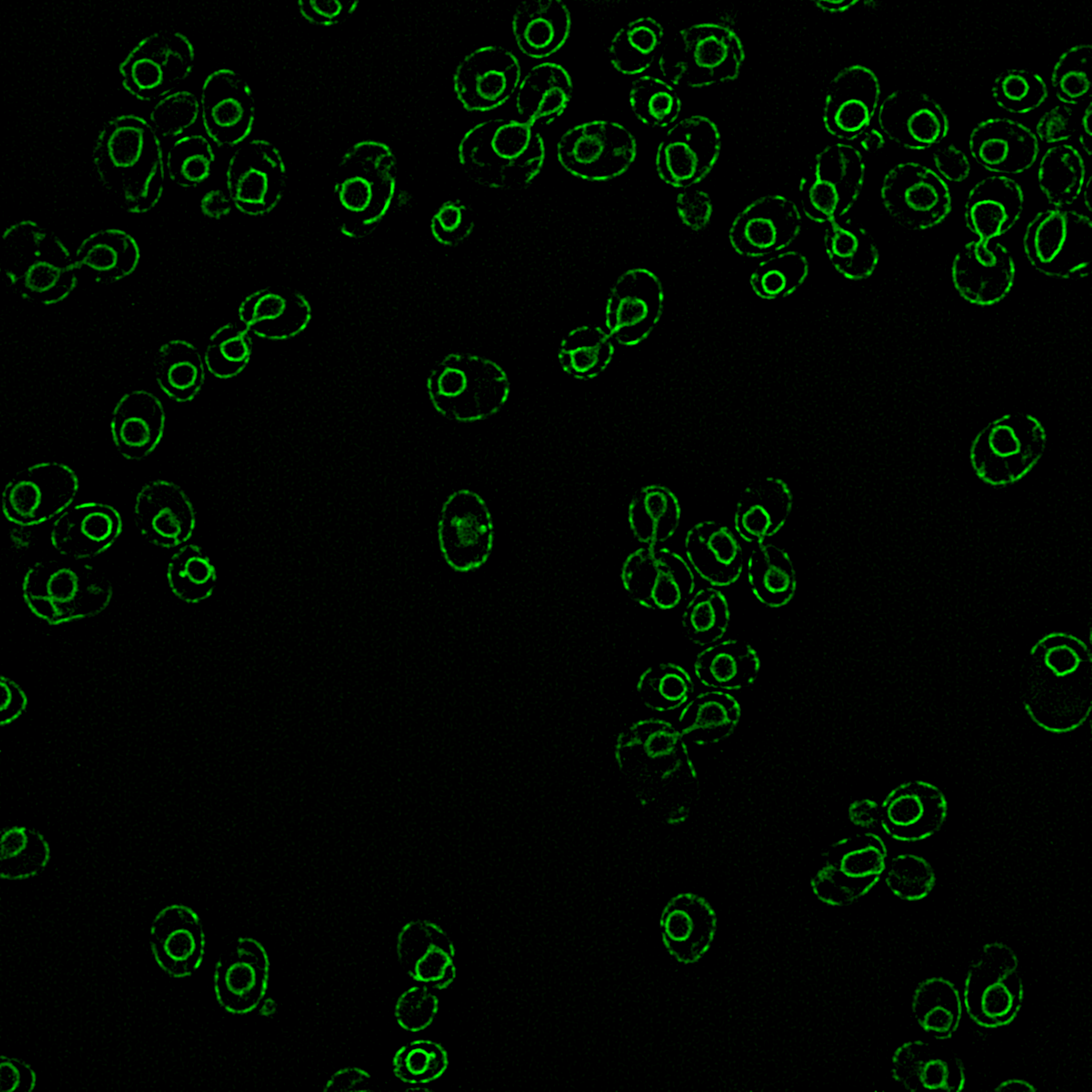

Supplement: Supplementary file 8 — Source data Fig. 7 [file 44318_2024_355_MOESM8_ESM.zip › Figure 7/7H/Image 3z21 Lro1 vector (Sec63-mCh).tif]

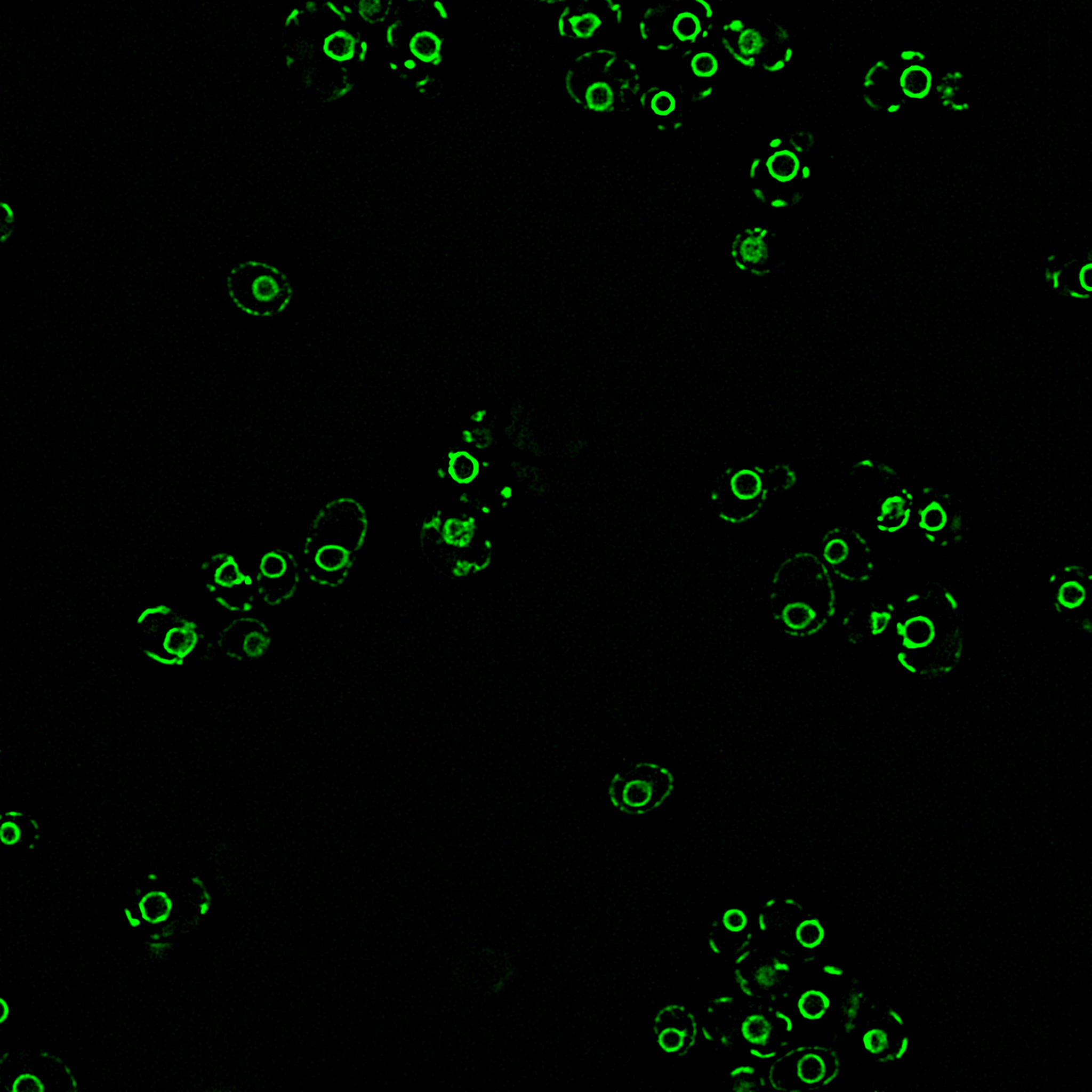

Supplement: Supplementary file 8 — Source data Fig. 7 [file 44318_2024_355_MOESM8_ESM.zip › Figure 7/7H/Image 8z22 Lro1* Pah1-7A (Sec63-mNG).tif]

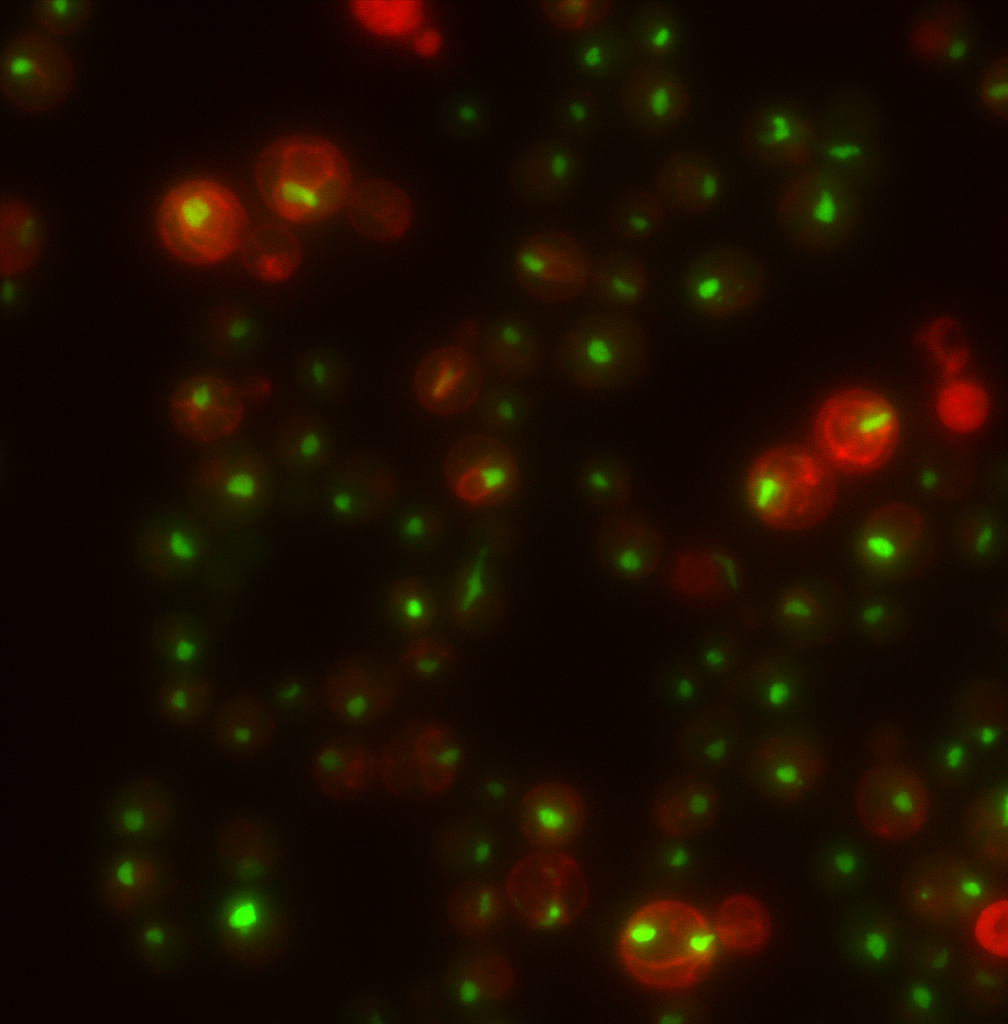

Supplement: Supplementary file 9 — Source data Fig. 8 [file 44318_2024_355_MOESM9_ESM.zip › Figure 8/8A/Merge pah1D.tif]

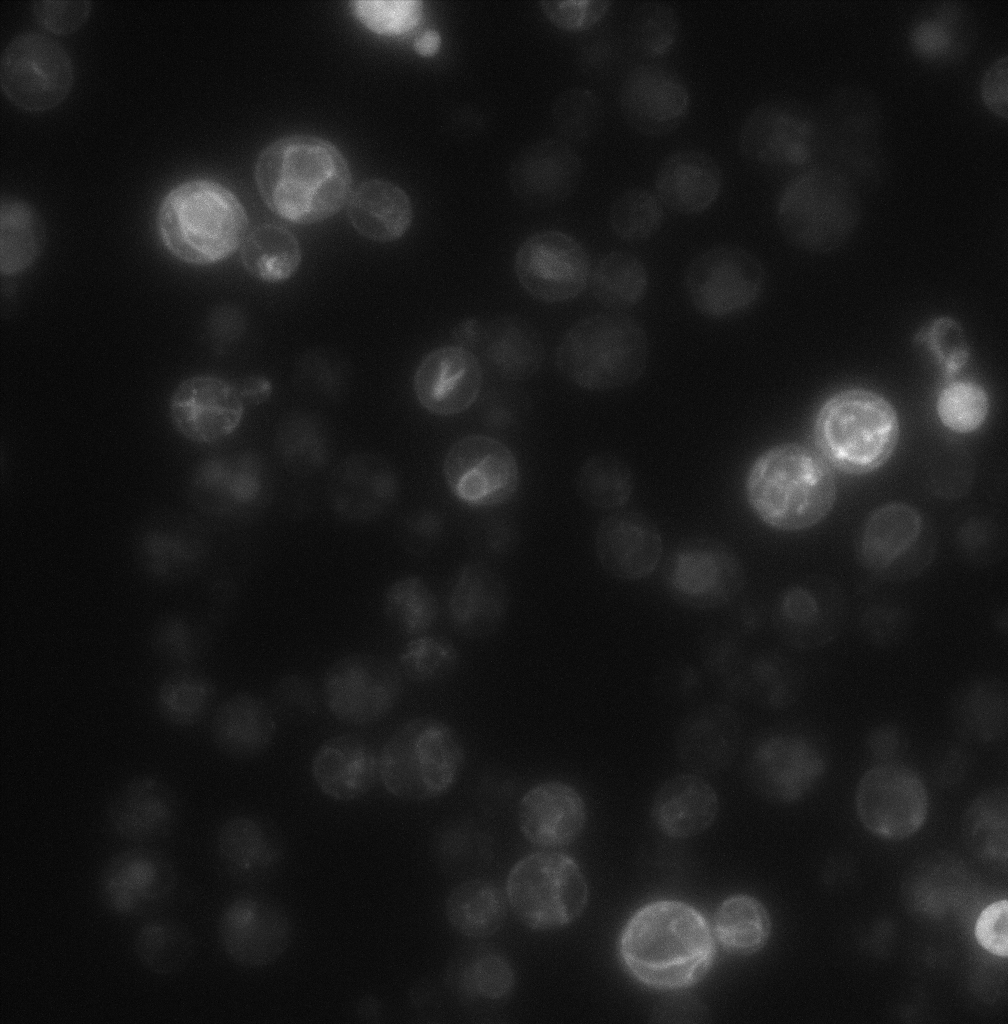

Supplement: Supplementary file 9 — Source data Fig. 8 [file 44318_2024_355_MOESM9_ESM.zip › Figure 8/8A/Image 1 pah1D (Lro1-mCh).tif]

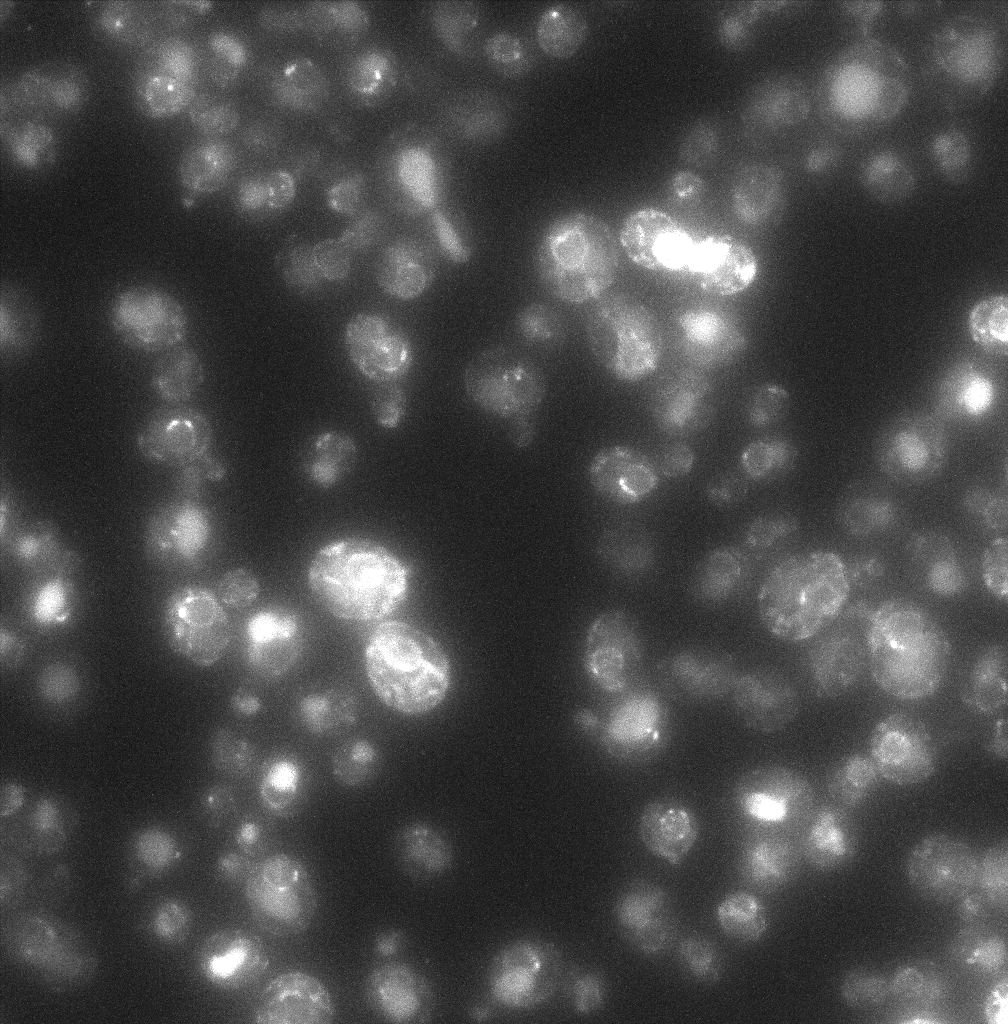

Supplement: Supplementary file 9 — Source data Fig. 8 [file 44318_2024_355_MOESM9_ESM.zip › Figure 8/8A/Image 14 PAH1 (Lro1-mCh).tif]

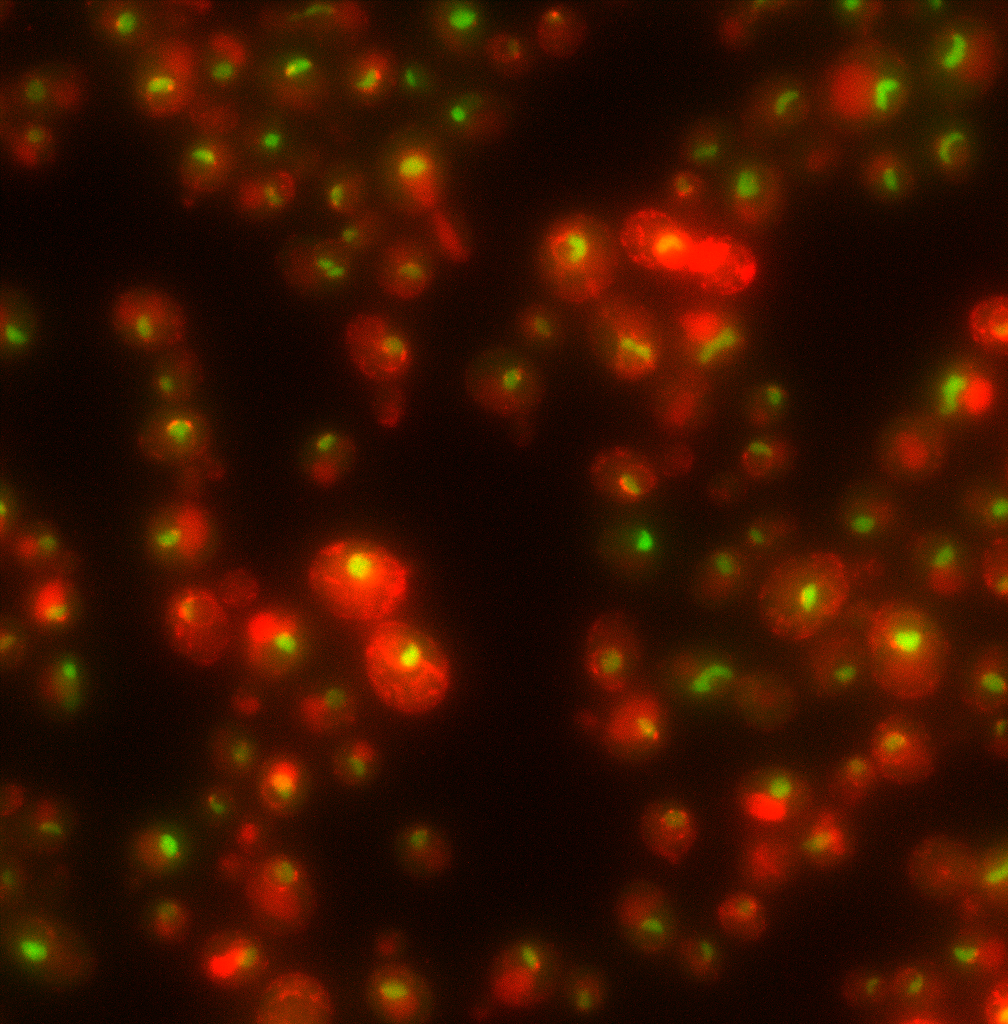

Supplement: Supplementary file 9 — Source data Fig. 8 [file 44318_2024_355_MOESM9_ESM.zip › Figure 8/8A/Merge PAH1.tif]

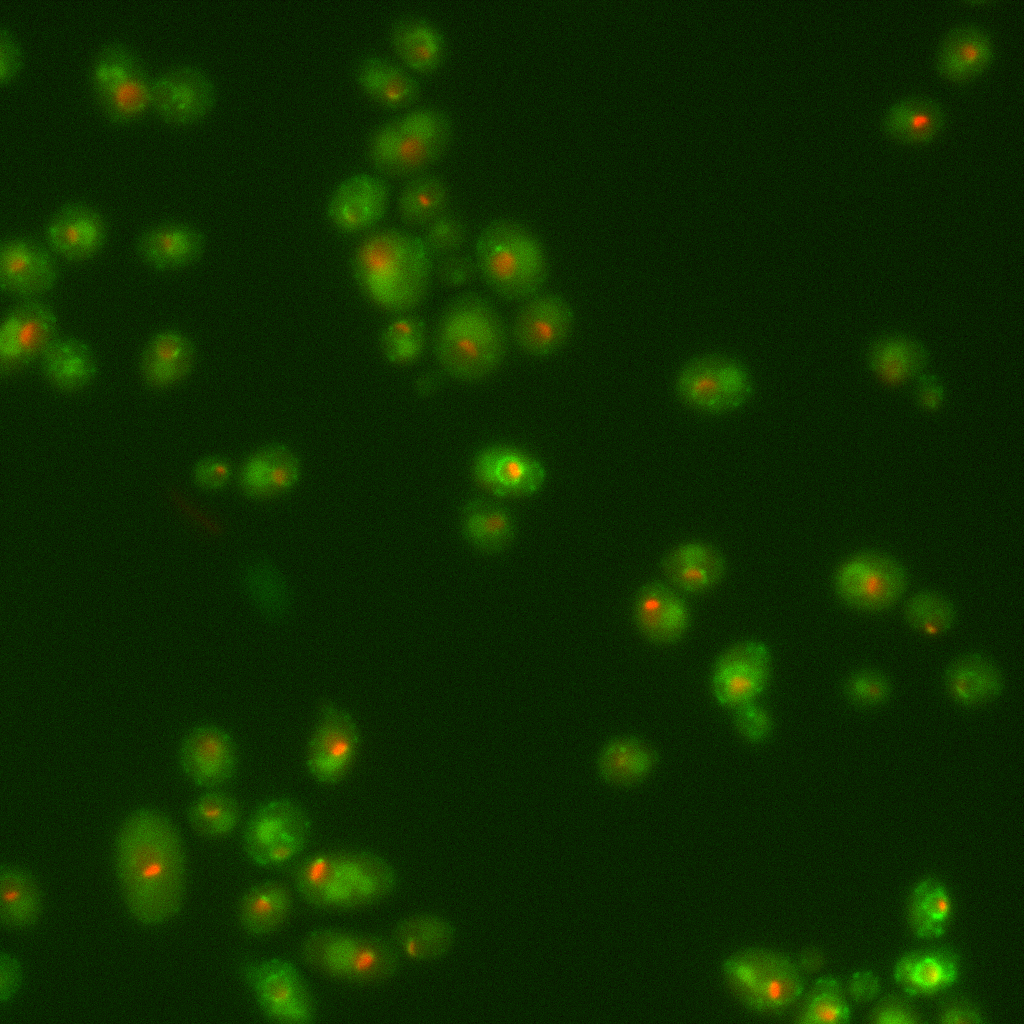

Supplement: Supplementary file 9 — Source data Fig. 8 [file 44318_2024_355_MOESM9_ESM.zip › Figure 8/8B/Merge Pah1-7A.tif]

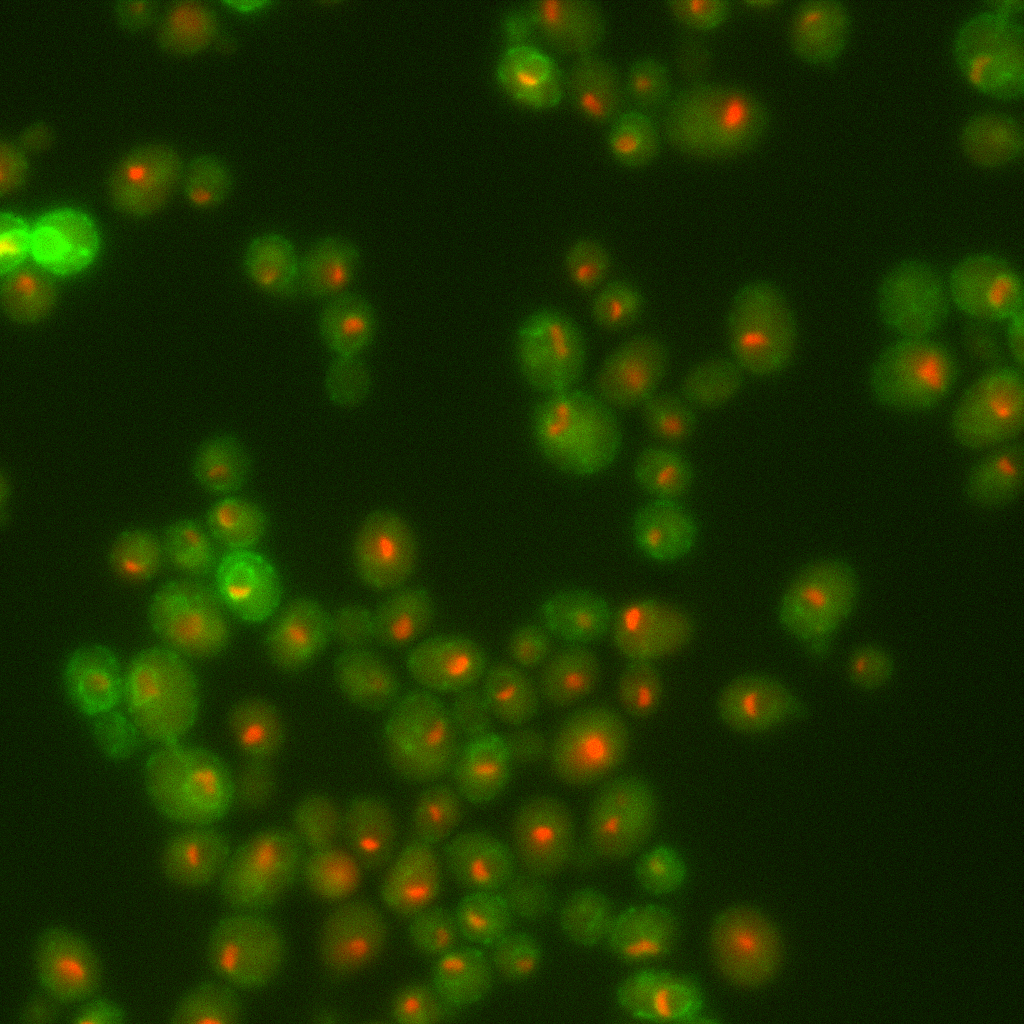

Supplement: Supplementary file 9 — Source data Fig. 8 [file 44318_2024_355_MOESM9_ESM.zip › Figure 8/8B/Merge Vector.tif]

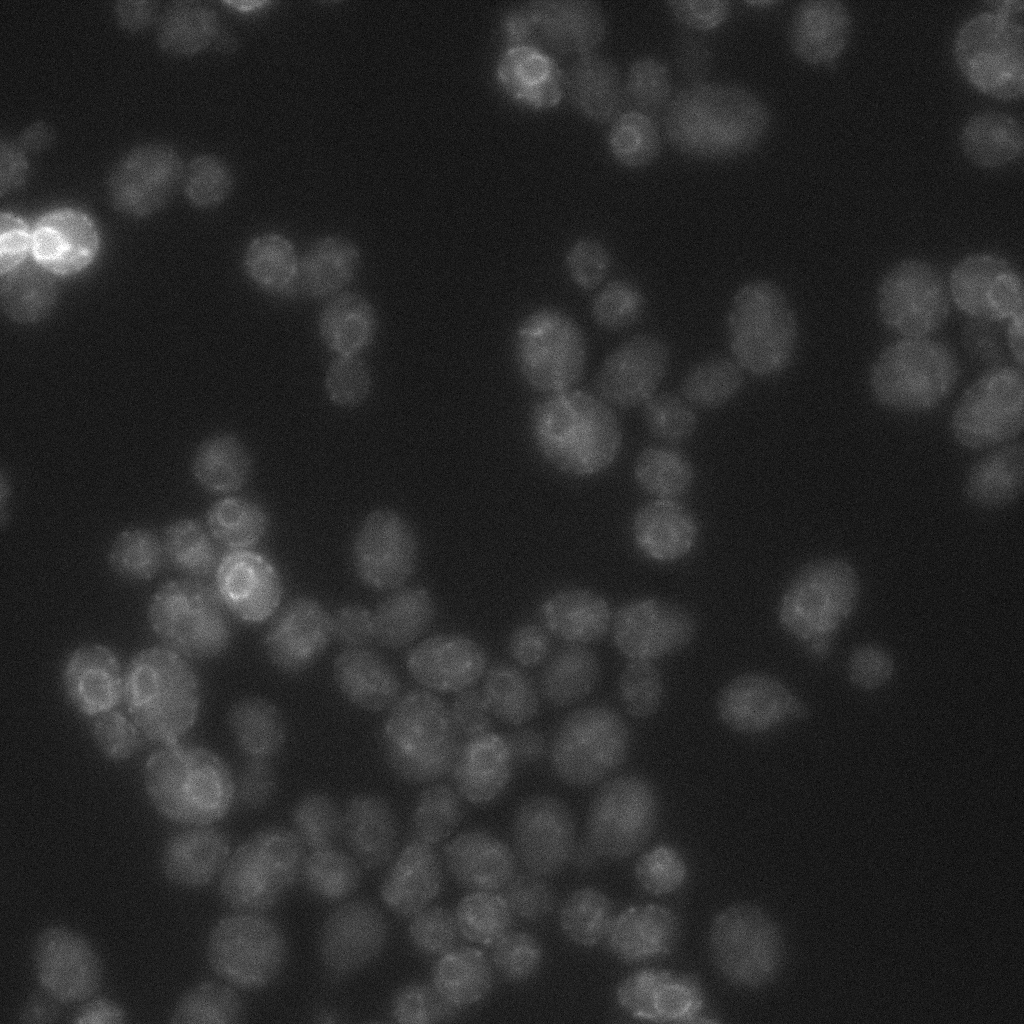

Supplement: Supplementary file 9 — Source data Fig. 8 [file 44318_2024_355_MOESM9_ESM.zip › Figure 8/8B/Image 24z10 Vector (Lro1*-mNG).tif]

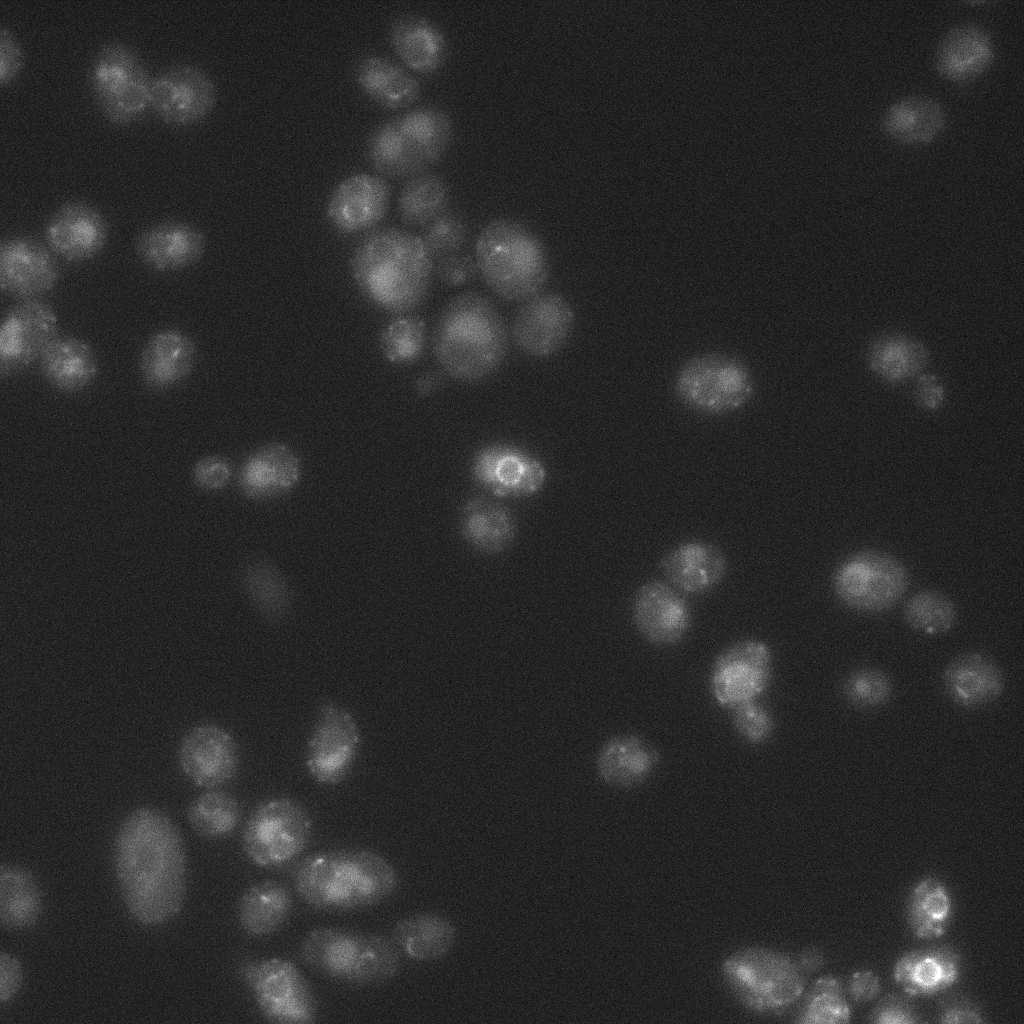

Supplement: Supplementary file 9 — Source data Fig. 8 [file 44318_2024_355_MOESM9_ESM.zip › Figure 8/8B/Image 33z11 Pah1-7A (Lro1*-mNG).tif]
