# Supplementary material for: Partitioning of fatty acids between membrane and storage lipids controls ER membrane expansion
Source: EMBO J. 2025 Jan 3;44(3):781–800. doi: 10.1038/s44318-024-00355-3 (PMC11790888; doi:10.1038/s44318-024-00355-3)
Supplement: Supplementary file 10 — Expanded View Figures [file 44318_2024_355_MOESM10_ESM.pdf]

Expanded View Figure

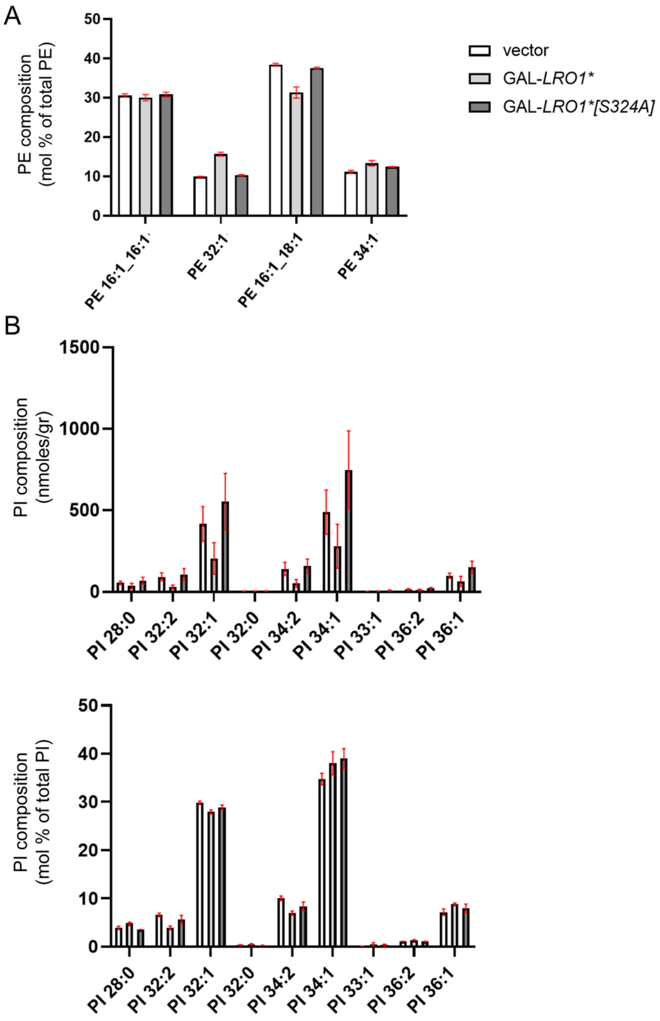

Figure EV1. Wild-type cells (BY4741) carrying the indicated constructs were grown in galactose as in Fig. 3A and processed for lipidomics analysis as described under Methods; data are means ± SD from three experiments.

(**A**) Analysis of the major PE species. (**B**) Analysis of PI species; PI data are shown both as nmoles/gr (top panel) and mol% of total PI (bottom panel).

 © The Author(s)
